# Effects of Grape Seed Proanthocyanidins on Growth Performance, Jejunal Antioxidant Capacity, Gut Microbial Diversity, and Metabolites in Kangle Chickens

**DOI:** 10.3390/ani15101481

**Published:** 2025-05-20

**Authors:** Qianqian Wang, Qingcan Fan, Xue Yang, Wei Hu, Lucheng Zheng, Lijun Zhou, Jinmeng Shi, Xingxu Zhao, Yong Zhang

**Affiliations:** 1 College of Veterinary Medicine, Gansu Agricultural University, Lanzhou 730070, China; 999360@jxycu.edu.cn; 2 College of Life Science and Resources and Environment, Yichun University, Yichun 336000, China; huoshan8fan@126.com (Q.F.);

**Keywords:** grape seed proanthocyanidins, intestinal morphology, oxidative stress, gut microbiota, metabolomic analysis

## Abstract

This study examined the effects of dietary supplementation with grape seed proanthocyanidins (GSPs) on the growth performance, serum biochemistry, jejunal antioxidant capacity, and jejunal microbiota and metabolites in Chinese indigenous Kangle chicken. In this experiment, 120 female Kangle chickens aged 30 days old were randomly allocated into three treatment groups: a control group (CON) fed a standard diet and two experimental groups fed diets supplemented with 200 mg/kg (LGSP) or 400 mg/kg (HGSP) of GSPs. The experiment consisted of a 7-day adaptation period followed by a 30-day feeding trial. The results demonstrated that GSP supplementation did not significantly improve their average daily gain or feed efficiency. However, the HGSP group showed significant improvements in their liver and jejunal indices, a reduced jejunal crypt depth, and increased villus-height-to-crypt-depth ratios compared to these values in the CON group. Furthermore, the HGSP group also exhibited elevated concentrations of cholesterol in their serum. Additionally, the oxidative stress levels were probably reduced in the jejuna of the HGSP group, as evidenced by reduced malondialdehyde (MDA) contents. Although jejunal microbial diversity remained unchanged, the metabolomic analysis identified significant upregulation of jejunal metabolites, particularly those associated with free radical scavenging, protein nutrition, and bile acid metabolism, which would be beneficial for maintaining intestinal health. These findings indicate that supplementing their diet with 400 mg/kg of GSPs could improve the health of Kangle chickens, underscoring their potential as a functional feed additive in the production of indigenous Chinese chickens.

## Simple Summary

This study examined the effects of dietary supplementation with grape seed proanthocyanidins (GSPs) on the growth performance, intestinal health, and metabolic functions of Chinese indigenous Kangle chicken (*Gallus gallus*). The results demonstrated that dietary supplementation with 400 mg/kg of the GSPs significantly increased their liver and jejunal indices while reducing the depth of the jejunal crypt. It might also reduce intestinal oxidative stress levels, as indicated by reduced malondialdehyde (MDA) contents. Furthermore, the GSPs modulated the metabolite composition in the jejunal content, resulting in the upregulation of physiological functions associated with protein nutrition, bile acid metabolism, and free radical scavenging, which would be beneficial for maintaining intestinal health. Supplementing feed with 400 mg/kg GSPs is beneficial for promoting health in Kangle chickens.

## 1. Introduction

Grape seed proanthocyanidins (GSPs) are polyphenolic compounds isolated from grape seeds, primarily composed of flavan-3-ol monomers (such as catechin and epicatechin) that are polymerized via C4–C8 or C4–C6 interflavan bonds to form oligomers and higher polymers. Monomers, dimers, and trimers are partially absorbed through passive diffusion or paracellular transport, whereas the other polymers mainly undergo intestinal microbial catabolism to generate phenolic acids prior to systemic absorption [1]. It has been reported that polyphenolic compounds engage in antioxidant defense through multifaceted mechanisms. Primarily, they directly scavenge reactive oxygen species (ROS) and reactive nitrogen species (RNS), thereby attenuating oxidative-stress-mediated cellular damage. Furthermore, these compounds enhance the systemic antioxidant capacity by activating the nuclear factor erythroid 2-related factor 2 (Nrf2) signaling cascade and modulating the transcriptional regulation of cytoprotective enzymes [2]. Additionally, their catechol moieties enable the chelation of free divalent iron ions, which suppresses iron absorption, disrupts Fenton reaction kinetics, and impedes hydroxyl radical formation [3]. Studies have shown that dietary supplementation with 400 mg/kg of GSPs significantly improved the feed efficiency in 21-day-old broilers while increasing the serum total antioxidant capacity (T-AOC) and glutathione peroxidase (GSH-Px) and catalase (CAT) activities and reducing malondialdehyde (MDA) levels [4]. Supplementation of their feed with 250 mg/kg of GSPs effectively reduced the serum levels of inflammatory cytokines induced by aflatoxin B1 in broilers and upregulated the expression of genes downstream of the Nrf2 pathway such as *heme oxygenase-1* (*HO-1*), *glutathione peroxidase 1* (*GPX1*), *NAD(P)H quinone dehydrogenase 1* (*NQO1*), and the *glutamate cysteine ligase catalytic subunit* (*GCLC*), thereby enhancing the antioxidant defense system [5]. Furthermore, in vitro experiments demonstrated that treatment with 5 μg/mL of GSPs for 72 h significantly reduced the ROS levels in D-galactose (D-GAL)-induced chicken ovarian tissues, increased the activities and gene expression of the antioxidant enzymes GSH-Px and superoxide dismutase (SOD), and decreased the MDA contents [6]. These findings indicate that GSPs exhibit a robust antioxidant capacity in both in vivo and in vitro systems.

Intestinal health is closely linked to animal production performance. The intestinal tract plays a crucial role in digesting feed and assimilating nutrients, which significantly impacts development and overall performance [7]. Additionally, it has been reported that GSPs regulate intestinal metabolism through multiple pathways, improving the gut’s physiological functions. Gong et al. [8] reported that GSP supplementation significantly increased the intestinal villus height and the villus-height-to-crypt-depth ratio in H_2_O_2_-stressed weaned rabbits, enhanced the activities of intestinal antioxidant enzymes, and reduced the MDA contents. Furthermore, dietary supplementation with grape seed extract (GSE) significantly increased the abundance of *Enterococcus* in the ileum and *Escherichia*, *Lactobacillus*, *Enterococcus*, and *Clostridium* in the cecum, thereby facilitating equilibrium between beneficial and pathogenic bacterial populations [9]. Additionally, dietary GSPs elevated cecal metabolites such as acetic acid, propionic acid, and butyric acid in geese, which are beneficial for maintaining intestinal health [10]. Collectively, these findings demonstrate that GSPs enhance intestinal functionality by improving intestinal morphology, reducing tissue stress levels, and modulating the gut’s microbial composition and metabolite profiles.

However, the effects of GSPs on indigenous Chinese chicken breeds have yet to be comprehensively elucidated, and their impacts on the jejunal microbiota and metabolome remain poorly characterized. This study elucidates the effects of GSPs on growth performance and intestinal health in Kangle chickens (*Gallus gallus*), a highly adaptable indigenous breed, thereby bridging this knowledge gap.

## 2. Materials and Methods

### 2.1. The Experimental Animals and Treatments

This experiment was ethically approved by the Animal Management and Use Committee of Yichun College (JXSTUDKY2022009). A total of 120 female Kangle chickens aged 30 days old and of similar body weights were randomly selected from a flock at the Yichun University breeding base. The chickens were housed in rectangular cages with a stocking density of 0.07 m^2^ per bird. Each cage was equipped with a nipple-type automatic drinking system. During the experimental period, the ambient temperature was rigorously controlled within a range of 20–26 °C, with dynamic adjustments implemented in response to the spatial distribution patterns of the flock, while the relative humidity was maintained at 50–60%, and a standardized 12 h photoperiod was consistently applied. Ad libitum access to feed and water was provided, with daily manure removal protocols enforced to uphold sanitary standards. The birds were allocated into three treatment groups, with each group consisting of eight replicates containing five chickens. The groups were treated as follows: (1) the control group (CON) was fed a basal diet; (2) the low-dose grape seed proanthocyanidin (LGSP) group was fed a basal diet supplemented with 200 mg/kg of GSPs; and (3) the high-dose grape seed proanthocyanidin (HGSP) group was fed a basal diet supplemented with 400 mg/kg of GSPs. The standard diet was designed in compliance with the Chicken Feeding Standard (NY/T 33-2004) [11]. The composition and nutritional profile of the experimental basal diet are detailed in Table 1. The dietary intervention trial lasted 37 days, consisting of a 7-day acclimation period followed by a 30-day experimental phase for systematic data acquisition. The dietary GSPs (with a purity > 98%) were procured from Shandong Jiade Biotechnology Co., Ltd. (Dezhou, China), with oligomeric proanthocyanidins constituting 75.4% of the total proanthocyanidin content, and the dosage selection was informed by established dose–response relationships in chicken models [4].

### 2.2. The Sample Collection and Processing

At the conclusion of the feeding trial, the experimental chickens were subjected to a 12 h fasting period, and their body weights were recorded. Whole blood samples were collected from the brachial vein and centrifuged at 1500× *g* for 10 min to isolate the serum, which was transferred into cryogenic vials and stored at −80 °C. One bird per replicate was euthanized via jugular venipuncture exsanguination under the ethical guidelines. The hepatic, jejunal, and splenic tissues were aseptically excised using sterile instruments, fat and connective tissue were carefully stripped from the surface, and they were weighed to determine the organ indices. A 4 cm segment of the proximal jejunum was dissected, debrided of extraneous adipose tissue, and longitudinally incised, and the luminal contents were flushed using three sequential saline lavages (0.9% NaCl). The anterior segment was fixed in 4% neutral-buffered paraformaldehyde (pH 7.4) for the histomorphometric analysis, whereas the posterior segment was snap-frozen in liquid nitrogen for antioxidant profiling. The residual jejunal tissue was cut longitudinally with scissors, and the contents were surgically harvested using dissection forceps, immediately flash-frozen, and archived at −80 °C.

### 2.3. Measurement of the Indicators and Methods

#### 2.3.1. Growth Performance

Their body weights were measured at the beginning (initial body weight, IBW) and conclusion (final body weight, FBW) of the study. Their feed consumption was recorded daily, and metrics such as their daily weight gain (ADG), average daily feed intake (ADFI), and feed conversion ratio (FCR) were calculated. The organ indices, including the liver index (LI), the jejunum index (JI), and the spleen index (SI), were determined based on the organ weights relative to body weight.

#### 2.3.2. Serum and Jejunal Tissue Indicators

The concentrations of total protein (TP), albumin (ALB), aspartate aminotransferase (AST), alanine aminotransferase (ALT), glucose (GLU), triglycerides (TGs), and total cholesterol (TC) in the serum were analyzed using the Hitachi 7180 Automatic Biochemical Analyzer (Hitachi, Tokyo, Japan). The jejunal antioxidant capacity, including T-AOC (A015-3-1) and SOD (A001-1), CAT (A007-1-1), GSH-Px (A005-1), and MDA (A003-1) contents, was assessed using commercial kits (Nanjing Jiancheng Bioengineering Institute, Nanjing, China) with the AF2200 Pro Microplate Reader (Eppendorf, Hamburg, Germany).

#### 2.3.3. Jejunal Histology

The jejunal tissues fixed in 4% paraformaldehyde were dehydrated, embedded into paraffin, sectioned, and stained using hematoxylin and eosin (HE). Villus height (VH), crypt depth (CD), and the ratio of villus height to crypt depth (V/C) were observed and measured using the BX43F upright microscope (Olympus, Tokyo, Japan).

### 2.4. Microbiota 16s rRNA Sequencing of the Jejunal Contents

Samples of the jejunal content from the CON and HGS groups were retrieved from the −80 °C cryopreservation unit and transferred to Shanghai Personal Biotechnology Co., Ltd. (Shanghai, China) for Illumina NovaSeq 6000 platform-based 16S rRNA gene sequencing. The experimental workflow comprised the following steps: the intestinal content specimens (0.2–0.5 g) underwent total genomic DNA extraction, with the DNA concentration and purity assessed using a NanoDrop NC2000 spectrophotometer (Thermo, Waltham, MA, USA). The hypervariable V3–V4 regions of bacterial 16S rRNA genes were amplified by employing universal primers (341F: 5′-ACTCCTACGGGAGGCAGCA-3; 806R: 5′-GGACTACHVGG GTWTCTAAT-3′), followed by 2% agarose gel electrophoresis and recycling of the target fragments via the AxyPrep DNA Gel Recovery Kit (Axygen, Corning, NY, USA). Purified amplicons were quantified via the Quant-iT PicoGreen dsDNA Assay Kit (Invitrogen, Carlsbad, CA, USA). Equimolar pools (30 ng/library) were prepared for Illumina-compatible library construction using the TruSeq Nano DNA LT Library Prep Kit (Illumina, San Diego, CA, USA). The final libraries were normalized to 2 nM, denatured with 0.1 N NaOH, and sequenced (2 × 250 bp paired-end) on the NovaSeq 6000 platform (Illumina, San Diego, CA, USA).

### 2.5. The Metabolomic Analysis of the Jejunal Contents

Untargeted metabolomic profiling was carried out utilizing liquid chromatography–mass spectrometry (LC-MS) by Shanghai Personal Biotechnology Co., Ltd. Eight samples were extracted and then separated using an Agilent 1290 Infinity LC UHPLC (Agilent, Santa Clara, CA, USA) and subsequently analyzed using a Triple TOF 6600 (Agilent, Santa Clara, CA, USA). Detection was performed in both electrospray ionization (ESI) positive and negative ion modes. The mass-to-charge ratio (m/z) range was set to 60–1000 Da for primary ion detection and 25–1000 Da for secondary fragment ion detection. Raw data were analyzed using ProteoWizard v3.0.2 and XCMS v3.7.1 software to align peaks, adjust the retention times, and extract the peak intensities. Metabolites were structurally identified by matching their molecular mass, collision energy, retention time, and secondary fragmentation spectra with these values in the databases. The identification level was confirmed to be Level 2 or higher, ensuring a high degree of confidence in the metabolite annotations. The metabolomics data were uploaded to MetaboLights with the study identifier MTBLS12181.

### 2.6. The Statistical Analysis

The data were preliminarily organized using WPS Office. The normality of the data was assessed using the Shapiro–Wilk test in SPSS v22.0 (SPSS Inc., Chicago, IL, USA). A one-way ANOVA with LSD post hoc testing was applied for a significance analysis, and the results were expressed as the mean and the error of the mean (SEM).

For 16S rRNA microbiome profiling, operational taxonomic unit (OTU) feature sequences were aligned against reference sequences from the Greengenes database v13.8 to assign taxonomic classifications. The microbial diversity analysis was performed in QIIME2 v2023.2, with alpha diversity assessed via the Kruskal–Wallis test and beta diversity visualized through non-metric multidimensional scaling (NMDS). Intergroup differences were evaluated using a permutational multivariate analysis of variance (PERMANOVA). Differentially abundant taxa were identified using MetagenomeSeq and visualized in Manhattan plots. Predictive functional profiling of the microbial communities was conducted using PICRUSt2 v2.5.2 and the MetaCyc database v2023.05.

For metabolomic sequencing, metabolite identification was conducted using a combination of a locally constructed database and public database searches including Metlin, the Human Metabolome Database (HMDB), mzCloud, and MassBank. The preprocessed data were subjected to a multivariate statistical analysis using the ropls package in R v4.4.2. An orthogonal partial least squares discriminant analysis (OPLS-DA) was employed to identify differential metabolites. An enrichment analysis of the metabolic pathways was performed based on the Kyoto Encyclopedia of Genes and Genomes (KEGG) database. The cluster analysis utilized a Euclidean distance matrix with complete linkage, and visualization was executed via the GenesCloud platform (https://www.genescloud.cn, (accessed on 10 January 2025)).

## 3. Results

### 3.1. The Effects of Dietary GSPs on the Growth Performance of Kangle Chickens

As shown in Table 2, compared to the CON group, both the LGSP and HGSP groups exhibited no statistical significance in their FBW, ADG, and FCRs (*p* > 0.05).

### 3.2. The Effects of Dietary GSPs on the Visceral Organ Indices of the Kangle Chickens

According to Table 3, the LIs and JIs were notably increased in the HGSP group compared to those in the CON group (*p* < 0.05), whereas the LGSP group showed no significant changes (*p* > 0.05). Additionally, compared to the CON group, the SI did not show significant changes in either the LGSP or HGSP group (*p* > 0.05).

### 3.3. The Effects of the Dietary GSPs on the Serum Biochemical Parameters of the Kangle Chickens

As shown in Table 4, compared to those in the CON group, the serum levels of ALB, AST, ALT, and TP did not show significant changes in the GSP-supplemented groups (*p* > 0.05). However, the serum GLU concentrations manifested a marked reduction in the LGSP group (*p* < 0.05), whereas no statistically significant alterations were observed in the HGSP group (*p* > 0.05). Additionally, TC levels exhibited a significant differential change in the HGSP group relative to those in the CON group (*p* < 0.05).

### 3.4. The Effects of the Dietary GSPs on the Jejunal Morphology of Kangle Chickens

As shown in Table 5, compared to that in the CON group, the jejunal VH did not differ significantly in the experimental groups (*p* > 0.05). However, the HGSP group exhibited a significant reduction in CD (*p* < 0.05) and a significant increase in the V/C (*p* < 0.05).

### 3.5. The Effects of the Dietary GSPs on the Jejunal Antioxidant Capacity of the Kangle Chickens

According to Table 6, no significant variations were observed in the T-AOC or SOD or GSH-Px activity in the jejunal tissues of the experimental groups compared to these values in the CON group (*p* > 0.05). However, the CAT activity was notably lower in both the LGSP and HGSP groups (*p* < 0.05). Furthermore, MDA exhibited a significant decrease in the HGSP group (*p* < 0.05).

### 3.6. The Effects of the GSPs on the Jejunal Microbiota Composition in the CON and HGSP Groups

#### 3.6.1. Analysis of the Jejunal Microbiota Composition

**Figure 1:**
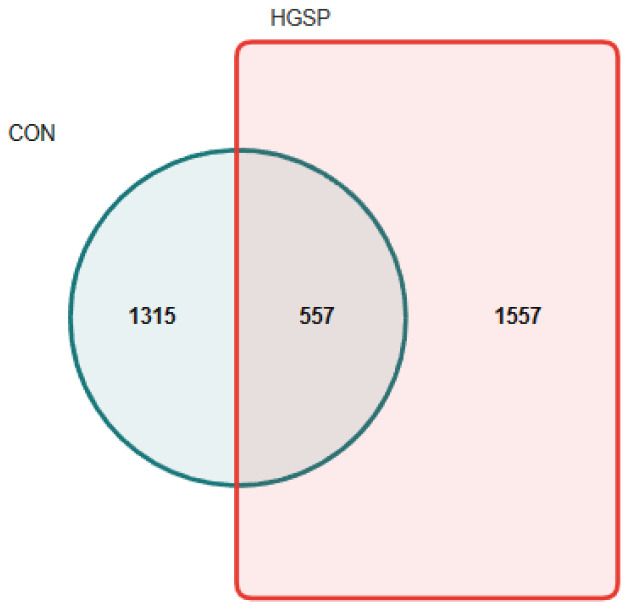
The common and unique OTUs between the CON and HGSP groups, where each colored block represents a distinct group, with overlapping regions between blocks indicating the shared OTUs among the corresponding groups. The numbers within each block denote the count of OTUs contained within that specific region; CON and HGSP were chickens fed the basal diet supplemented with 0 and 400 mg/kg of GSPs, respectively.

The analysis of the 16S rRNA sequencing data yielded an average of 76,653 high-quality bacterial gene sequences per sample, with an average read length of 430 base pairs. As illustrated in Figure 1, a combined total of 3429 OTUs were detected across all samples. Among these, the CON group contained 1315 unique OTUs (38.35% of the total OTUs), while the HGSP group contained 1557 unique OTUs (40.19% of the total OTUs). Both groups shared 557 OTUs, accounting for 45.41% of the total OTUs.

**Figure 2:**
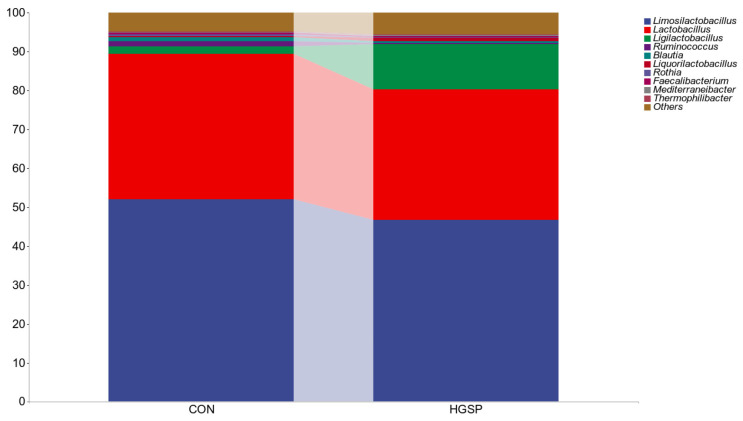
The composition of the jejunum microbiota of Kangle chicken at the genus level in the CON and HGSP groups, where the x-axis represents the names of the groups in the grouping scheme, the y-axis represents the relative abundance of each taxonomic unit at a specific taxonomic level, and the legend section lists the names of the top 10 most abundant genera. CON and HGSP were chickens fed the basal diet supplemented with 0 and 400 mg/kg of GSPs, respectively.

At the phylum level, no significant differences were observed between the CON and HGSP groups (*p* > 0.05), and the dominant phylum in the jejunum was Firmicutes, accounting for over 90% of the total microbiota. The top 10 genera in both groups included *Limosilactobacillus*, *Lactobacillus*, *Ligilactobacillus*, *Ruminococcus*, *Blautia*, *Liquorilactobacillus*, *Rothia*, *Faecalibacterium*, and *Thermophilibacter* (Figure 2). Among these, *Limosilactobacillus*, *Lactobacillus*, and *Ligilactobacillus* were the dominant genera. In the CON group, their relative abundances were 51.97%, 37.44%, and 1.86%, respectively, while in the HGSP group, they were 46.75%, 33.59%, and 11.59%, respectively.

#### 3.6.2. The Analysis of the Jejunal Microbiota Diversity Between the CON and HGSP Groups

**Figure 3:**
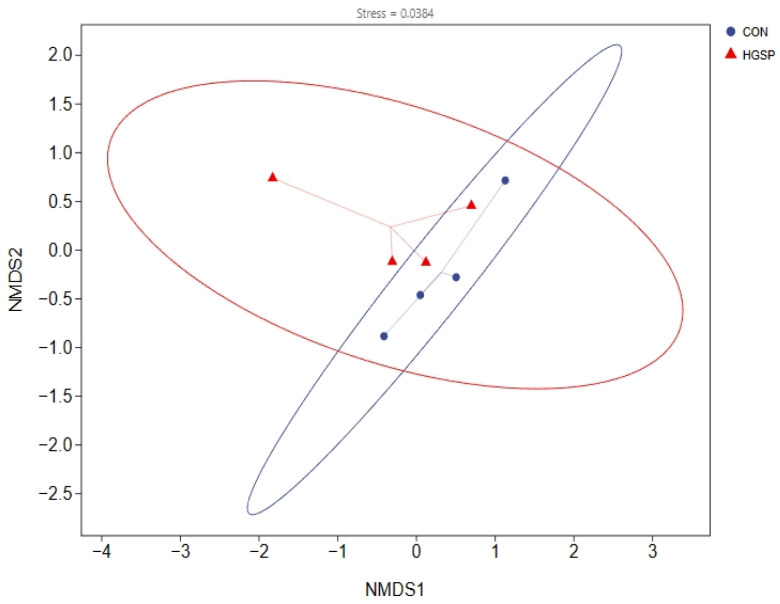
The outcomes of the NMDS analysis. Each point in the figure represents a sample, with distinct colors denoting different sample groups. The proximity of two points can be approximated to reflect smaller differences in microbial community composition between samples. CON and HGSP were chickens fed the basal diet supplemented with 0 and 400 mg/kg of the GSPs, respectively.

The alpha diversity indices of the jejunal microbiota were calculated, followed by an intergroup comparative analysis. Compared to those in the CON group, the HGSP group showed no significant differences in its alpha diversity indices, including the Simpson, Chao1, Shannon, and Pielou indices (*p* > 0.05), suggesting that the dietary GSPs did not significantly affect the alpha diversity of the jejunal microbiota in the Kangle chickens. The beta diversity analysis, visualized using NMDS (Figure 3), revealed a stress value of less than 0.05, indicating that the NMDS plot accurately reflected the similarity structure between samples. The overlapping confidence ellipses of the CON and HGSP groups further suggested minimal differences between the two groups. Permanova testing revealed that the variations observed between the groups were not statistically meaningful (*p* > 0.05).

#### 3.6.3. Analysis of the Differential Microbial Taxa Between the CON and HGSP Groups

**Figure 4:**
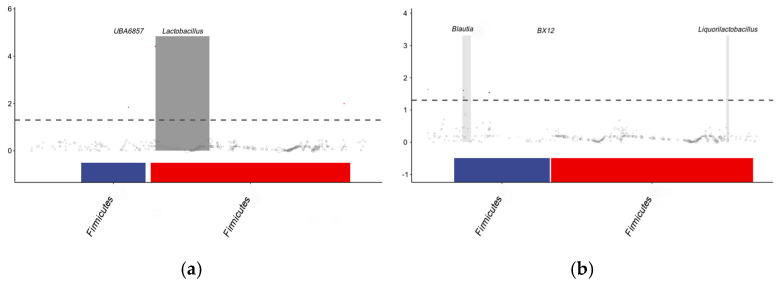
A Manhattan plot of the MetagenomeSeq analysis of the jejunal microbiota in the CON and HGSP groups. Figure (**a**) shows the upregulation in the HGSP group, while Figure (**b**) shows the upregulation in the CON group. CON and HGSP were chickens fed the basal diet supplemented with 0 and 400 mg/kg of the GSPs, respectively.

Differences in microbial composition between samples are often driven by changes in specific taxa rather than the entire community. As shown in Figure 4, the dietary GSPs significantly elevated the relative abundance of three bacterial taxa (*p* < 0.05) compared to that in the CON group, including an OTU classified under the genus *UBA6857* (*sp. 902792985*) and two uncharacterized lineages. Conversely, six taxa exhibited statistically significant depletion (*p* < 0.05), comprising *Aerococcaceae* (from the *Blautia* genus), *sp. 902363595* (from the *BX12* genus), and four taxonomically unclassified taxa.

### 3.7. The Metabolomic Analysis of the Jejunal Contents Between the CON and HGSP Groups

#### 3.7.1. The Chemical Composition of the Jejunal Metabolites

**Figure 5:**
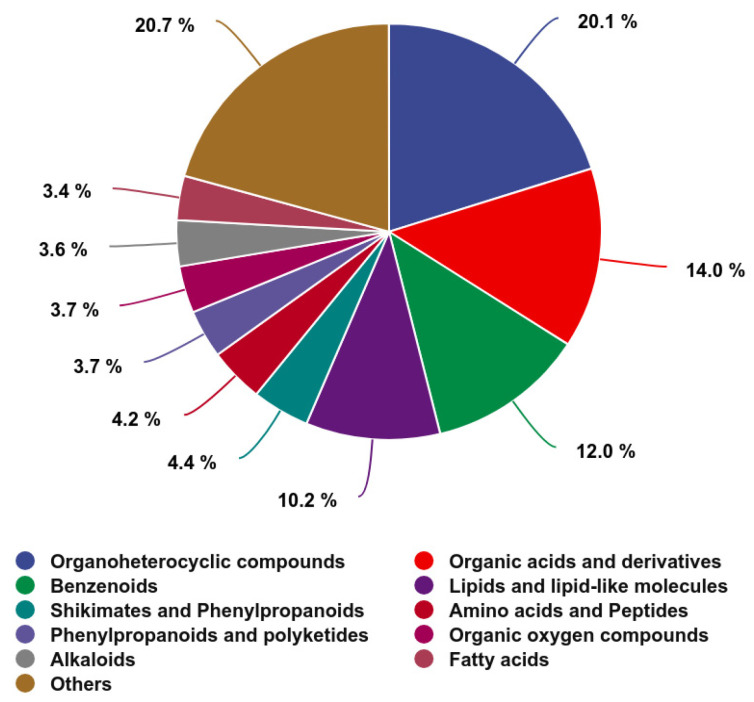
The chemical categories and proportions of all detected metabolites in the jejunal contents in both the CON and HGSP groups. The colored segments represent different chemical classification categories, and the percentages indicate the proportion of metabolites within each category relative to the total number of identified metabolites.

Metabolites in the jejunal contents were structurally identified using the established methods, with manual verification ensuring a confidence level of Level 2 or above. Overall, 2328 metabolites were detected, comprising 1359 in positive ionization mode and 969 in negative ionization mode. These metabolites were classified based on their chemical categories and visualized in a pie chart (Figure 5). The majority of the metabolites belonged to the following classes: Organoheterocyclic compounds (468 metabolites), Organic acids and derivatives (326 metabolites), Benzenoids (280 metabolites), Lipids and lipid-like molecules (238 metabolites), Shikimates and Phenylpropanoids (102 metabolites), Amino acids and Peptides (98 metabolites), Phenylpropanoids and polyketides (87 metabolites), Organic oxygen compounds (85 metabolites), Alkaloids (83 metabolites), and Fatty acids (80 metabolites). The remaining 481 metabolites were classified into other categories.

#### 3.7.2. The Statistical Analysis of the Differentially Abundant Metabolites Between the CON and HGSP Groups

**Figure 6:**
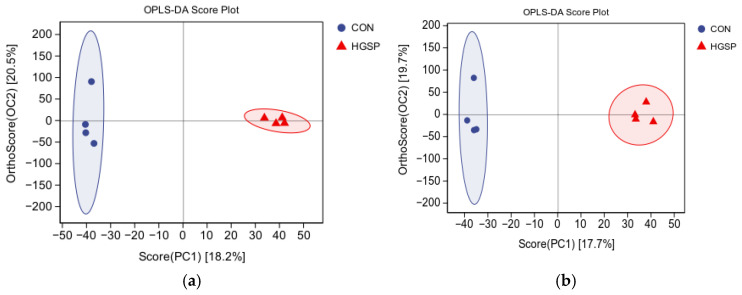
Score plots of the orthogonal partial least squares discriminant analysis (OPLS-DA) in the CON and HGSP groups. (**a**) Positive ion mode; (**b**) negative ion mode. CON and HGSP were chickens fed the basal diet supplemented with 0 and 400 mg/kg of the GSPs, respectively.

An orthogonal partial least squares discriminant analysis (OPLS-DA) was performed to qualitatively analyze the metabolites in the jejunal contents of the CON and HGSP groups. The Q2 values for both the positive and negative ion modes exceeded 0.5, indicating that the model was stable and reliable. As shown in Figure 6, distinct clustering of the CON and HGSP groups was evident, indicating notable variations in their metabolic patterns.

#### 3.7.3. Identification and Classification of Differentially Abundant Metabolites

**Figure 7:**
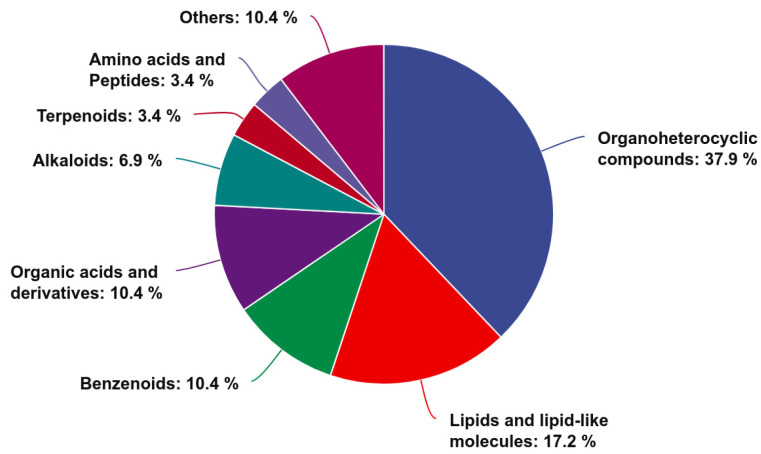
Chemical classification of the differentially abundant metabolites between the CON and HGSP groups.

**Figure 8:**
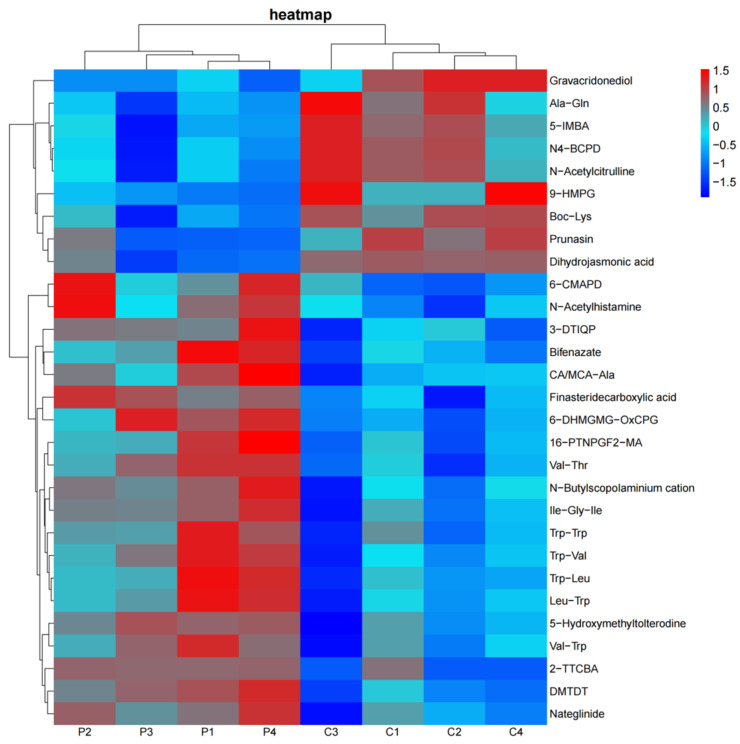
A heatmap of significantly different metabolites between the CON and HGSP groups. Red indicates upregulated metabolites; blue indicates downregulated metabolites. C1–C4 represent samples in the CON group, while P1–P4 represent samples in the HGSP group.

The results on the expression of all metabolites are shown in Supplementary Table S1. Using the criteria in the OPLS-DA of a VIP *>* 1, *p* < 0.05, and Log_2_FC > 2, a total of 29 differentially abundant metabolites were identified in both the positive and negative ion modes (*p* < 0.05). Among these, 20 metabolites were upregulated and 9 were downregulated in the HGSP group compared to the CON group. These metabolites were classified based on their chemical categories and visualized in a pie chart (Figure 7). The majority of the differentially abundant metabolites were classified into the following classes: Organoheterocyclic compounds (11 metabolites), Lipids and lipid-like molecules (5 metabolites), Benzenoids (3 metabolites), and Organic acids and derivatives (3 metabolites). A hierarchical clustering analysis was performed on the significantly different metabolites, and a heatmap was generated (Figure 8). The heatmap revealed clear clustering of the metabolites between the two groups. For clarity, some metabolite names with long descriptions were abbreviated, with the full names provided in Supplementary Table S1. Among the 20 upregulated metabolites in the HGSP group, 7 were oligopeptides (5 of which contained tryptophan), 5 were drugs or their derivatives, and 4 were heterocyclic compounds (including DMTDT, which contains a thioketone group, and 3-DTIQP, a phenolic compound), and the remaining 4 included a polyhydroxy glycoside (6-DHMGMG-OxCPG), a bile acid derivative (CA/MCA-Ala), N-Acetylhistamine, and a prostaglandin analog (16-PTNPGF2α-MA).

#### 3.7.4. The Enrichment Analysis of the Differentially Abundant Metabolites

**Figure 9:**
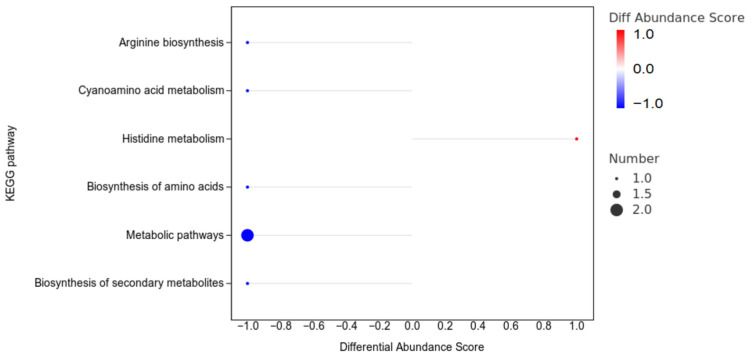
The enrichment analysis of the differentially abundant metabolites using KEGG pathways between the CON and HGSP groups. The Y-axis represents the names of the metabolic KEGG pathways, and the X−axis represents the differential abundance (DA) scores. The DA score reflects the overall change in the metabolites within a pathway, where a score of 1 indicates upregulation of all of the metabolites identified in the pathway, and a score of -1 indicates downregulation.

The enrichment analysis of the differentially abundant metabolites between the CON and HGSP groups is shown in Figure 9. Due to the predominance of metabolic derivatives among the identified metabolites, only a limited number of pathways were enriched. The histidine metabolism pathway was significantly upregulated in the HGSP group compared to the CON group (*p* < 0.05), while arginine biosynthesis, cyanoamino acid metabolism, amino acid biosynthesis, metabolic pathways, and secondary metabolite biosynthesis were downregulated (*p* < 0.05).

## 4. Discussion

It has been reported that GSPs reduce oxidative damage by neutralizing reactive oxygen species and stimulating the cellular pathways associated with antioxidant activity [13]. They also play a significant role in immune regulation and antibacterial activity, potentially improving animal health, maintaining organ function, and enhancing feed efficiency and productivity [14]. Nevertheless, this research found that supplementing their diet with GSPs did not lead to a notable enhancement in the growth metrics of Kangle chickens. This discrepancy in the results may be due to factors such as differences in the concentration of the GSPs and the experimental animals, which may have limited the growth-promoting effects of the GSPs. Farahat et al. [15] similarly reported that continuous supplementation of 145–2000 mg/kg of GSE into broilers’ diets did not significantly affect the growth traits of the broilers between 0 and 42 days, consistent with the findings of this study.

Organ indices, defined as the ratio of organ weights to live body weight, are indicative of organs’ functional capacity [16]. The intestinal tract plays a critical role in digesting feed, absorbing nutrients, and supporting immune responses, with its structural integrity being critical to maintaining selective barrier functions [17]. This study demonstrated that dietary supplementation with 400 mg/kg of the GSPs not only significantly increased the jejunal index but also enhanced the villus-height-to-crypt-depth ratio (V/C). These findings align with previous research by Erinle et al. [18], who observed substantial elevation in the V/C ratio in both the duodenum and jejunum of broilers fed grape-pomace-supplemented diets. These results indicate that the GSP intervention might have improved intestinal morphology and enhanced intestinal function.

Serum biochemical parameters serve as critical indicators of animal health and nutritional metabolism, reflecting the internal environment and physiological status of an organism [19,20]. This study demonstrated that the 400 mg/kg GSP supplementation significantly increased serum TC levels in the Kangle chickens, suggesting an improved nutritional status. This is consistent with the observed increases in their liver and jejunum indices, suggesting enhanced organ function leading to improved nutrient utilization. However, some studies have reported that procyanidine interferes with adipocyte differentiation and lipid metabolism in the liver, leading to reduced plasma TC and TG levels [21,22,23], which contrasts with the findings of this study. These discrepancies may be due to differences in the experimental conditions in their studies, such as high-fat diets or hyperlipidemic animal models. In this study, the chickens were in a growth phase with generally low nutritional levels, and the GSPs likely enhanced nutrient absorption, thereby promoted normal growth and development, indicating that GSPs modulate lipid metabolism in a context-dependent manner.

Accumulating evidence indicates that GSPs activate the Nrf2 signaling pathway, orchestrate the transcriptional regulation of antioxidant enzymes, and reduce the level of oxidative stress in the body [24,25]. The Nrf2-Keap1-ARE axis constitutes a pivotal defense mechanism against oxidative stress. Under oxidative challenge, Nrf2 dissociates from its cytoplasmic repressor Kelch-like ECH-associated protein 1 (Keap1), translocates into the nucleus, and binds to antioxidant response elements (AREs) to transactivate cytoprotective genes downstream [26]. Supporting this paradigm, Cao et al. [4] reported that broilers receiving 200 and 400 mg/kg of GSPs from hatching exhibited a significant increase in their serum GSH-Px activity and a reduction in their MDA levels by 21 days post-hatch. Wang et al. [27] demonstrated that 100 mg/kg GSP supplementation in oxidized oil-fed broilers significantly elevated their hepatic GSH-Px activity while suppressing MDA accumulation. In alignment with these studies, our results revealed that the 400 mg/kg GSP supplementation significantly reduced the jejunal MDA contents in Kangle chickens, while GSH-Px activity showed a numerical increase that did not reach statistical significance, which may be attributed to breed-specific variations. These findings suggest that GSPs probably enhance the intestinal antioxidant capacity in Kangle chickens and substantiate the antioxidative efficacy of GSPs in livestock.

The microbial community in the gut is essential for supporting animal health, development, and efficient nutrient utilization, with its composition being shaped by factors such as diet, age, and management practices [28]. The jejunum, a key site for nutrient digestion and absorption, has a lower microbial density compared to that of the cecum due to faster digesta passage [29]. This study identified fewer microbial species in the jejunum, which was consistent with previous findings. Dietary polyphenols can function as prebiotics, promoting beneficial bacteria and inhibiting harmful ones [30,31]. Although studies have shown that GSPs might increase the abundance of certain beneficial cecal bacteria, no significant alterations in the microbial diversity indices have been observed [4,18], which aligns with the results of this study.

Metabolomics refers to a comprehensive analysis of the metabolites or small molecules within a specific biological system. Metabolites can be classified into two main categories based on their origin and function: endogenous and exogenous. Endogenous metabolites are generated through intrinsic metabolic processes, including catabolism and anabolism, and are highly conserved across organisms. Exogenous metabolites, commonly referred to as xenobiotics, primarily originate from dietary intake or environmental exposure and display significant variability. Factors such as dietary habits, environmental exposure, and the composition and activity of the gut microbiota can profoundly influence the exogenous metabolome. In recent years, accumulating evidence has highlighted the significant impact of gut metabolites on host health [32,33,34].

Using LC-MS technology, this study identified 20 significantly upregulated metabolites in the jejunal contents of the Kangle chickens supplemented with 400 mg/kg of GSPs. Among these metabolites, six dipeptides and one tripeptide were detected. Dipeptides and tripeptides represent the primary forms of protein digestion and absorption, exhibiting a higher absorption efficiency compared to that for free amino acids [35]. They promote protein synthesis and cellular metabolism and contribute to antioxidant defense by donating hydrogen atoms to directly neutralize free radicals [36]. Among the six dipeptides detected, five contained tryptophan (Trp), an essential amino acid characterized by an indole ring structure [37]. Tryptophan is metabolized via the kynurenine, serotonin, and indole pathways, producing bioactive compounds such as kynurenine and serotonin, which play pivotal roles in regulating inflammation, immunity, and neural function [38,39]. Furthermore, tryptophan-containing dipeptides and tripeptides demonstrate significant antioxidant activity, primarily due to the indole ring structure of tryptophan [40].

Among the upregulated metabolites in the HGSP group, 6-DHMGMG-OxCPG is a polyhydroxy glycoside characterized by a cyclopentapyran ring, an epoxyethane ring, β-D-glucosyl, and α-L-mannosyl groups, with a feruloyl group esterified at the 3-position in α-L-mannosyl. The feruloyl moiety, which contains a phenolic hydroxyl group, can neutralize free radicals and chelate metal ions [41]. This compound exhibits structural and functional similarities to GSPs, indicating that it may represent an activated form of GSPs in the intestine. CA/MCA-Ala is a bile acid derivative formed through the conjugation of cholic acid with alanine. The conjugation with alanine may enhance the hydrophilicity of the molecule, thereby modulating its activity. Studies have shown that bile acids not only facilitate the solubilization and absorption of cholesterol and fat-soluble vitamins in the small intestine [42] but also exert a negative feedback effect to inhibit the rate-limiting enzyme of cholesterol synthesis (HMG-CoA reductase) in hepatocytes, thereby maintaining cholesterol homeostasis [43,44]. Furthermore, high concentrations of bile acids inhibit the growth of Gram-positive bacteria, such as *Clostridium* species, playing a crucial role in preserving the balance of the gut microbiota [45].

The enrichment analysis of the differentially abundant metabolites demonstrated that histidine metabolism was significantly upregulated in the high-dose GSP (HGSP) group. Histidine, an essential amino acid required for protein synthesis, is involved in numerous metabolic processes [46]. It acts as a precursor to the neurotransmitter histamine, which is synthesized by histidine decarboxylase and plays a crucial role in neural regulation [46]. Furthermore, histidine contributes to antioxidant defense through its imidazole side chain, which functions as a proton donor and acceptor, allowing it to neutralize free radicals and buffer cellular redox states [47]. Histidine metabolites, including urocanic acid, provide photoprotection and exhibit antioxidant effects in skin tissues [48]. Histidine also serves as the metabolic precursor to the antioxidant dipeptide carnosine. Carnosine exerts its cytoprotective effects by neutralizing free radicals, inhibiting lipid peroxidation, and suppressing the formation of advanced glycation end products (AGEs)—key molecular mediators implicated in oxidative stress and cellular aging [49].

## 5. Conclusions

In conclusion, dietary supplementation with 400 mg/kg of GSPs significantly enhanced intestinal function and reduced jejunal oxidative stress levels in Kangle chicken. Additionally, the dietary GSPs modulated the composition of jejunal metabolites, with the upregulated metabolites linked with protein and lipid digestion and absorption. Many of these metabolites exhibit free radical scavenging properties, consistent with the observed improvements in jejunal function and reduced oxidative stress. Nevertheless, these findings provide foundational insights into the application of GSPs as a functional feed additive in the production of indigenous Chinese chickens.

## Figures and Tables

**Table 1:** Composition and nutrient levels of basal diets (air-dry basis).

Ingredients	Content (%)	Nutrient Levels	Content (%)
Maize	66.70	Metabolizable energy (MJ/kg) ^2^	12.59
Soybean meal	27.35	Crude protein	17.68
Soybean oil	2.41	Calcium	0.75
Limestone	1.21	Phosphorus	0.56
Salt	0.21	Lysine	0.92
Calcium hydrogen phosphate	0.92	Methionine	0.42
L-lysine hydrochloride	0.11	Non-phytic acid phosphor	0.34
DL-Methionine	0.09		
Premix ^1^	1.00		
Total	100.00		

^1^ The premix provided the following per kg of the diets: Vitamin A: 9000 IU; vitamin B_1_: 5 mg; vitamin B_2_: 10 mg; vitamin B_6_: 12 mg; vitamin B_12_: 30 μg; vitamin D_3_: 2500 IU; vitamin E: 25 mg; vitamin K_3_: 3 mg; biotin: 0.10 mg; folic acid: 3 mg; D-pantothenic acid: 20 mg; nicotinic acid: 30 mg; Cu: 20 mg; Fe: 320 mg; Mn: 100 mg; Zn: 200 mg; I: 0.60 mg; Se: 0.30 mg. ^2^ The metabolizable energy (ME) of the experimental diet was calculated from the feed composition values acquired through near-infrared reflectance spectroscopy (NIRS), as described by Cozannet et al. [12]. Non-phytate phosphorus levels were quantified according to the Chicken Feeding Standard (NY/T 33-2004) [11].

**Table 2:** The effects of dietary grape seed proanthocyanidins on the growth performance of Kangle chickens.

Items ^1^	Treatments ^2^			SEM	*p*-Value
	CON	LGSP	HGSP		
IBW (g)	146.20	145.39	147.72	3.02	0.642
FBW (g)	471.74	489.64	483.14	8.15	0.488
ADG (g/d)	10.85	11.48	11.18	0.31	0.538
ADFI (g/d)	36.83	37.64	36.47	0.92	0.846
FCR	3.39	3.28	3.26	0.14	0.491

^1^ IBW, initial body weight; FBW, final body weight; ADG, average daily weight gain; ADFI, average feed intake; FCR, feed conversion ratio. ^2^ CON, LGSP, and HGSP were chickens fed the basal diet supplemented with 0, 200, and 400 mg/kg of grape seed proanthocyanidins, respectively.

**Table 3:** The effects of dietary grape seed proanthocyanidins on the visceral organs of the Kangle chickens.

Items	Treatments ^1^			SEM	*p*-Value
	CON	LGSP	HGSP		
Liver index (%)	2.99 ^b^	2.87 ^b^	3.39 ^a^	0.08	0.004
Spleen index (%)	0.24	0.24	0.23	0.01	0.871
Jejunum index (%)	3.06 ^b^	3.02 ^b^	3.76 ^a^	0.17	0.036

^1^ CON, LGSP, and HGSP were chickens fed the basal diet supplemented with 0, 200, and 400 mg/kg of grape seed proanthocyanidins, respectively. ^a,b^ Distinct superscripts within the same row indicate significant differences between groups.

**Table 4:** The effects of dietary grape seed proanthocyanidins on the serum biochemical parameters of the Kangle chickens.

Items ^1^	Treatments ^2^			SEM	*p*-Value
	CON	LGSP	HGSP		
TP (g/L)	33.28	33.42	33.32	0.59	0.728
ALB (g/L)	14.28	13.72	14.52	0.25	0.490
AST (U/L)	238.60	233.50	220.50	7.72	0.759
ALT (U/L)	3.20	3.67	4.17	0.26	0.296
GLU (mmol/L)	11.65 ^a^	10.43 ^b^	10.82 ^ab^	0.20	0.026
TG (mmol/L)	0.84	0.91	1.49	0.13	0.074
TC (mmol/L)	2.32 ^b^	2.53 ^ab^	2.87 ^a^	0.09	0.028

^1^ TP, total protein; ALB, albumin; AST, aspartate aminotransferase; ALT, alanine aminotransferase; GLU, glucose; TG, triglyceride; TC, total cholesterol; ^2^ CON, LGSP, and HGSP were chickens fed the basal diet supplemented with 0, 200, and 400 mg/kg of grape seed proanthocyanidins, respectively. ^a,b^ Distinct superscripts within the same row indicate significant differences between groups.

**Table 5:** The effects of dietary grape seed proanthocyanidins on the jejunal morphology of the Kangle chickens.

Items ^1^	Treatments ^2^			SEM	*p*-Value
	CON	LGSP	HGSP		
VH (μm)	1043.66	1143.96	1063.26	27.98	0.342
CD (μm)	212.72 ^a^	179.77 ^ab^	139.99 ^b^	14.05	0.046
V/C	5.20 ^b^	6.57 ^ab^	7.59 ^a^	0.44	0.022

^1^ VH, villus height; CD, crypt depth; V/C, the ratio of villus height to crypt depth; ^2^ CON, LGSP, and HGSP were chickens fed the basal diet supplemented with 0, 200, and 400 mg/kg of grape seed proanthocyanidins, respectively. ^a,b^ Distinct superscripts within the same row indicate significant differences between groups.

**Table 6:** The effects of diets supplemented with grape seed proanthocyanidins on the jejunal antioxidant parameters of Kangle chickens.

Items ^1^		Treatments ^2^			SEM	*p*-Value
		CON	LGSP	HGSP		
Jejunum	T-AOC (μmol/mg prot)	64.04	67.17	68.44	3.70	0.300
	SOD (U/mg prot)	148.45	153.09	149.88	5.42	0.640
	CAT (U/mg prot)	9.13 ^a^	6.30 ^b^	6.56 ^b^	0.49	0.021
	GSH-Px (U/mg prot)	33.13	37.23	44.20	1.99	0.073
	MDA (nmol/mg prot)	0.24 ^a^	0.22 ^a^	0.13 ^b^	0.02	0.002

^1^ T-AOC, total antioxidant capacity; SOD, superoxide dismutase; CAT, catalase; GSH-Px, glutathione peroxidase; MDA, malondialdehyde. ^2^ CON, LGSP, and HGSP were chickens fed the basal diet supplemented with 0, 200, and 400 mg/kg of grape seed proanthocyanidins, respectively. ^a,b^ Distinct superscripts within the same row indicate significant differences between groups.

## Data Availability

We have uploaded the sequencing raw data for this study to the MetaboLights database (https://www.ebi.ac.uk/metabolights/reviewer52a0a096-064f-4666-8396-1fe0fee917c9, (accessed on 16 January 2025)) under reference number MTBLS12181.

## Supplementary Materials

The following supporting information can be downloaded at https://www.mdpi.com/article/10.3390/ani15101481/s1. Table S1: The results on the expression of all metabolites.
