# Supplementary material for: Effects of Grape Seed Proanthocyanidins on Growth Performance, Jejunal Antioxidant Capacity, Gut Microbial Diversity, and Metabolites in Kangle Chickens
Source: Animals (Basel). 2025 May 20;15(10):1481. doi: 10.3390/ani15101481 (PMC12108165; doi:10.3390/ani15101481)
Supplement: Supplementary file 1 [file animals-15-01481-s001.zip › animals-3568718-supplementary.pdf]

Supplementary Materials

Table S1. The results of expression of all metabolites

ID	log2FC	pvalue	vip	Reg	Name	Abbreviation
M1016.pos	2.08	0.02	1.85	UP	Trp-Leu	Trp-Leu
M1041.neg	2.30	0.01	2.16	UP	2-(2,3-Dimethylphenyl)-7-methyl-1,2,4-triazaspiro[4.5]decane-3-thione	DMTDT
M1150.neg	2.31	0.02	1.89	UP	6-(3-Chloro-4-methylanilino)-2,4(1H,3H)-pyrimidinedione	6-CMAPD
M1208.pos	2.02	0.01	2.10	UP	Finasteridecarboxylic acid	Finasteridecarboxylic acid
M1259.pos	2.86	0.02	1.95	UP	N-Butylscopolaminium cation	N-Butylscopolaminium cation
M1285.neg	2.09	0.03	1.92	UP	Nateglinide	Nateglinide
M1411.neg	2.06	0.02	2.01	UP	3-(5,5-Dimethyl-5,6-dihydro[1,2,4]triazolo[3,4-a]isoquinolin-3-yl)phenol	3-DTIQP
M1449.pos	2.08	0.04	1.86	UP	5-Hydroxymethyltolterodine	5-Hydroxymethyltolterodine
M1959.pos	2.70	0.01	1.94	UP	Bifenazate	Bifenazate
M2020.neg	2.58	0.00	2.17	UP	(1aS,1bS,2S,5aR,6S,6aS)-6-((6-Deoxy-3-O-((2E)-3-(4-hydroxy-3-methoxyphenyl)prop-2-enoyl)-.alpha.-L-mannopyranosyl)oxy)-1a-(hydroxymethyl)-1a,1b,2,5a,6,6a-hexahydrooxireno[4,5]cyclopenta[1,2-c]pyran-2-yl .beta.-D-glucopyranoside	6-DHMGMG-OxCPG
M2055.pos	2.51	0.02	1.89	UP	16-Phenoxytetranorprostaglandin F2.alpha. methylamide	16-PTNPGF2α-MA
M2235.neg	2.39	0.05	1.89	UP	2-{[4-(Thiophen-2-yl)-1,3-thiazol-2-yl]carbamoyl}benzoic acid	2-TTCBA
M298.pos	2.11	0.02	1.85	UP	N-Acetylhistamine	N-Acetylhistamine
M518.neg	2.02	0.01	1.94	UP	Cholic acid/Muricholic acid, alanine-conjugated (2)	CA/MCA-Ala
M600.neg	2.14	0.01	2.04	UP	Val-Thr	Val-Thr
M610.neg	2.09	0.03	1.93	UP	Ile-Gly-Ile	Ile-Gly-Ile
M612.pos	2.41	0.01	1.99	UP	Trp-Val	Trp-Val
M670.neg	2.28	0.05	1.78	UP	Val-Trp	Val-Trp
M728.pos	2.52	0.04	1.78	UP	Trp-Trp	Trp-Trp
M729.neg	2.20	0.02	1.88	UP	Leu-Trp	Leu-Trp
M1306.pos	-2.61	0.01	2.01	Down	5-(3-Indolylmethylene)barbituric acid	5-IMBA
M1412.pos	-2.14	0.01	2.00	Down	N4-Butyl-6-chloro-4,5-pyrimidinediamine	N4-BCPD
M155.pos	-2.32	0.01	2.01	Down	N-Acetylcitrulline	N-Acetylcitrulline
M1652.pos	-2.04	0.02	2.04	Down	9-Hydroxy-4-methoxypsoralen_9-glucoside	9-HMPG
M1894.pos	-2.51	0.02	2.07	Down	N-.alpha.-(tert-Butoxycarbonyl)-L-lysine	Boc-Lys
M2068.pos	-2.00	0.02	2.01	Down	Gravacridonediol	Gravacridonediol
M2358.pos	-3.92	0.04	1.90	Down	Prunasin	Prunasin
M405.neg	-2.37	0.01	2.00	Down	Ala-Gln	Ala-Gln
M572.neg	-2.31	0.04	1.99	Down	Dihydrojasmonic acid	Dihydrojasmonic acid
M1.neg	1.03	0.37	0.82	Nodiff	Prolylalanine	
M10.neg	1.07	0.16	1.36	Nodiff	Leucylphenylalanine	
M100.neg	2.43	0.11	1.58	Nodiff	Phenylsulfate	
M1000.pos	0.11	0.43	0.76	Nodiff	2-Phenyl-N-(1-phenylethyl)quinoline-4-carboxamide	
M1001.neg	0.07	0.78	0.33	Nodiff	1-(7H-Purin-6-yl)-N-(3-(trifluoromethyl)phenyl)piperidine-4-carboxamide	
M1002.pos	0.12	0.53	0.64	Nodiff	Lys-Gln	

M1003.pos	-0.10	0.42	0.78	Nodiff	Gly-Pro-Arg
M1004.neg	0.65	0.19	1.36	Nodiff	Zanamivir
M1005.neg	0.53	0.22	1.29	Nodiff	.beta.-Alanylglycine
M1006.pos	-1.82	0.25	1.03	Nodiff	1-Acetyl-4-piperidinecarboxylic acid
M1007.neg	-0.90	0.03	1.74	Nodiff	Ala-Ser
M1008.pos	0.21	0.49	0.79	Nodiff	Salicylaldehyde
M1009.pos	0.13	0.47	0.69	Nodiff	Methyl [(5-methoxy-1H-benzimidazol-2-yl)sulfanyl]acetate
M101.neg	0.07	0.96	0.13	Nodiff	Cholesteryl sulfate
M1010.pos	0.35	0.33	1.04	Nodiff	Citrusinol
M1011.pos	0.33	0.92	0.15	Nodiff	Pro-Trp
M1012.neg	-0.45	0.99	0.01	Nodiff	Zidovudine
M1013.pos	-0.24	0.44	0.87	Nodiff	2-(4-Amino-1-piperidinyl)acetamide
M1014.neg	-0.67	0.69	0.45	Nodiff	Chenodeoxycholic acid 24-acyl-.beta.-D-glucuronide
M1015.pos	0.15	0.54	0.62	Nodiff	.alpha.-Hydroxyalprazolam
M1017.neg	0.04	0.62	0.46	Nodiff	6-Benzyl-3-butan-2-yl-9-(7-hydroxy-6-oxooctyl)-1,4,7,10-tetrazabicyclo[10.4.0]hexadecane-2,5,8,11-tetrone
M1018.pos	0.05	0.93	0.10	Nodiff	N-Methylpropionamide
M1019.neg	1.66	0.15	1.47	Nodiff	2-(1,3-Thiazol-4-yl)-1H-benzimidazol-5-amine
M102.neg	-1.41	0.06	1.82	Nodiff	2-Aminobutyric acid
M1020.pos	-0.13	0.69	0.36	Nodiff	Methylgingerol
M1021.pos	-0.09	0.47	0.69	Nodiff	4-Phenyl-4-piperidinecarbonitrile
M1022.neg	-0.01	0.76	0.31	Nodiff	5,7-Dichloro-2-methyl-1H-indole
M1023.pos	2.73	0.17	1.31	Nodiff	9Z,11E,13E-Octadecatrienoic acid methyl ester
M1024.neg	-0.44	0.40	1.00	Nodiff	Arachidonic sulfonic acid
M1025.neg	0.64	0.20	1.35	Nodiff	3-Hydroxyphenylaceitic acid sulfate
M1026.pos	-0.18	0.62	0.48	Nodiff	LPC(O-18:1)
M1027.pos	-0.41	0.53	0.61	Nodiff	His-Lys
M1028.pos	0.71	0.34	0.93	Nodiff	5,6,7,8-Tetrahydrothieno[2,3-b]quinolin-4-amine
M1029.neg	0.65	0.28	1.08	Nodiff	Met-Trp
M103.neg	-0.41	0.24	1.10	Nodiff	Mannitol
M1030.pos	-0.41	0.25	1.07	Nodiff	4,4,6-Trimethyl-1-(4-methylphenyl)-1,4-dihydro-2-pyrimidinethiol
M1031.pos	1.02	0.18	1.34	Nodiff	4,4-Dimethyl-4,5-dihydro-1,3-oxazol-2-amine
M1032.pos	-1.21	0.02	2.03	Nodiff	N-Methyl-1-deoxynojirimycin
M1033.pos	-0.02	0.55	0.67	Nodiff	Lotaustralin
M1034.neg	0.53	0.54	0.55	Nodiff	Thr-Asn
M1035.neg	-0.07	0.90	0.17	Nodiff	4-Hydroxy-4'-chlorobiphenyl
M1036.pos	-0.56	0.60	0.55	Nodiff	(Diethylamino)(oxo)acetic acid
M1037.neg	-0.47	0.94	0.10	Nodiff	(Methylsulfanyl)heptyl glucosinolate
M1038.pos	-1.19	0.03	1.84	Nodiff	Isovalerylsarcosine
M1039.neg	0.72	0.06	1.68	Nodiff	Egtazic acid
M104.neg	1.06	0.29	1.09	Nodiff	Isovaleric acid
M1040.pos	0.15	0.48	0.81	Nodiff	4-Methyl-3-(trifluoromethyl)-1H-pyrazol-5-amine
M1042.pos	0.00	0.46	0.83	Nodiff	Tanshinone I
M1043.pos	-1.31	0.33	1.03	Nodiff	Gln-Met
M1044.pos	-0.12	0.37	0.93	Nodiff	4-Fluoro-6-phenoxy-2-pyrimidinamine
M1045.pos	-0.69	0.27	1.05	Nodiff	Threoninyl-Hydroxyproline
M1046.pos	0.21	0.56	0.60	Nodiff	6-Benzylaminouracil
M1047.pos	1.45	0.08	1.68	Nodiff	6-(4-Methyl-1-piperazinyl)-N-(5-methyl-1H-pyrazol-3-yl)-2-[(1E)-2-phenylethenyl]-4-pyrimidinamine
M1048.pos	-1.23	0.53	0.62	Nodiff	1-O-Octadecyl-sn-glyceryl-3-phosphorylcholine
M1049.pos	0.50	0.25	1.12	Nodiff	LysoPC(0:0/18:0)
M105.neg	-0.38	0.44	0.85	Nodiff	Glucose 1-phosphate

M1050.neg	-0.74	0.30	0.94	Nodiff	6-Chloro-5-fluoro-1H-1,3-benzodiazol-2-amine
M1051.pos	0.07	0.98	0.07	Nodiff	9(10)-Epoxy-12Z-octadecenoic acid
M1052.pos	-0.08	0.85	0.29	Nodiff	Carnequinazoline A
M1053.neg	-0.01	0.82	0.32	Nodiff	3-Hydroxy-2-[(Z)-oct-2-enyl]pentanedioic acid
M1054.pos	2.33	0.14	1.40	Nodiff	Oleic acid methyl ester
M1055.neg	-0.49	0.40	1.00	Nodiff	Triptophenolide
M1056.neg	0.04	1.00	0.06	Nodiff	o-Nitrobenzoic acid
M1057.neg	-0.78	0.35	0.90	Nodiff	2-Amino-6-bromo-3-fluorobenzoic acid
M1058.pos	1.35	0.27	1.09	Nodiff	Pro-Lys
M1059.neg	-0.29	0.31	1.02	Nodiff	5,7,3',4',5'-Pentahydroxyflavone
M106.pos	0.89	0.08	1.63	Nodiff	Ala-Glu
M1060.pos	1.74	0.05	1.73	Nodiff	4-Isoxazolepropanoic acid, .alpha.-amino-2,3-dihydro-5-methyl-3-oxo-
M1061.neg	0.17	0.45	0.78	Nodiff	(1Z)-1-(3-Ethyl-5-hydroxy-1,3-benzothiazol-2-ylidene)acetone
M1062.pos	0.33	0.26	1.14	Nodiff	Biphenylindanone A
M1063.pos	0.90	0.33	0.96	Nodiff	Bindone
M1064.neg	0.33	0.31	1.08	Nodiff	(2E)-4-Hydroxybut-2-enoic acid
M1065.neg	0.58	0.59	0.67	Nodiff	Ile-Gln
M1066.pos	-0.54	0.64	0.48	Nodiff	Lonicerin
M1067.pos	0.08	0.47	0.78	Nodiff	Ruxolitinib
M1068.neg	0.80	0.10	1.55	Nodiff	Glu-Leu
M1069.pos	0.45	0.45	0.81	Nodiff	Leu-Tyr
M107.pos	0.12	0.65	0.46	Nodiff	Isomalt
M1070.pos	0.92	0.07	1.65	Nodiff	Polyoxyethylene_(600)_mono-_ricinoleate
M1071.pos	-1.49	0.32	0.88	Nodiff	1H-Pyrrole-2,5-dione, 1-[6-[[(17.beta.)-3-methoxyestra-1,3,5(10)-trien-17-yl]amino]hexyl]-
M1072.neg	-1.20	0.30	1.18	Nodiff	3-Sulfinoalanine
M1073.pos	-1.22	0.09	1.56	Nodiff	5-(3-Pyridinyl)-1,3,4-thiadiazol-2-ylamine
M1074.neg	0.72	0.36	0.79	Nodiff	5-(3,4'-Dihydroxy-4,4,7,8a-tetramethyl-6'-oxospiro[2,3,4a,5,6,7-hexahydro-1H-naphthalene-8,2'-3,8-dihydrofuro[2,3-e]isoindole]-7'-yl)pentanoic acid
M1075.pos	-0.90	0.17	1.37	Nodiff	Osmaronin
M1076.pos	0.08	0.71	0.34	Nodiff	1-[(Dimethylamino)carbonyl]-3-piperidinecarboxylic acid
M1077.pos	0.05	0.89	0.23	Nodiff	Tetraglyme
M1078.pos	-0.13	0.99	0.05	Nodiff	5beta-Pregnane-3alpha,17alpha,20alpha-triol-11-one
M1079.pos	1.07	0.32	0.99	Nodiff	(5E,9E)-Farnesylacetone
M108.pos	2.93	0.12	1.49	Nodiff	gamma-Glutamylmethionine
M1080.neg	-0.90	0.08	1.65	Nodiff	Quercetagetin
M1081.neg	0.46	0.55	0.54	Nodiff	4-Hydroxy-3-[(E)-7-hydroxy-3,7-dimethyl-4-oxooct-5-enyl]-5-[(E)-4-hydroxy-3-methylbut-2-enyl]benzoic acid
M1082.neg	0.38	0.46	0.72	Nodiff	6-(5-Bromo-2-hydroxyphenyl)-2-keto-4-phenyl-1H-pyridine-3-carbonitrile
M1083.pos	0.10	0.63	0.53	Nodiff	Gramine
M1084.pos	-0.13	0.73	0.38	Nodiff	2-[4-(Trifluoromethyl)phenyl]-1,3-thiazole-5-carboxylic acid
M1085.neg	0.55	0.38	0.92	Nodiff	4,4'-Sulfonylbisphenol
M1086.pos	0.60	0.49	0.67	Nodiff	4-[3-(Trifluoromethyl)benzyl]piperidine
M1087.neg	-0.58	0.29	0.99	Nodiff	Fluvoxamine_acid
M1088.neg	1.75	0.23	1.21	Nodiff	Ala-Asp
M1089.pos	0.50	0.25	1.22	Nodiff	Glu-Ser
M109.pos	-1.90	0.17	1.39	Nodiff	N1,N8-Diacetylspermidine
M1090.neg	-0.11	0.81	0.26	Nodiff	2,2'-(2-Hydroxyethylazanediyl)diacetic acid
M1091.pos	-0.11	0.44	0.87	Nodiff	Ciprofloxacin
M1092.pos	-1.10	0.04	1.85	Nodiff	Karanjin

M1093.pos	-1.25	0.01	2.12	Nodiff	6-(2-Thienyl)-2,4-hexadienoic acid isobutylamide
M1094.pos	1.06	0.32	1.05	Nodiff	3-Methylpentane-2,4-diyl dicarbamate
M1095.pos	0.09	0.34	0.99	Nodiff	Arginyl-Leucine
M1096.pos	-0.92	0.02	1.82	Nodiff	6-Chloro-N-cyclohexyl-1-methyl-1H-pyrazolo[3,4-d]pyrimidin-4-amine
M1097.pos	-0.28	0.89	0.10	Nodiff	2-Chloro-4-(dimethylamino)-5-fluoropyrimidine
M1098.pos	0.14	0.49	0.78	Nodiff	Metronidazole
M1099.neg	1.26	0.99	0.10	Nodiff	Benserazide
M11.neg	1.07	0.16	1.36	Nodiff	Phe-Leu
M110.neg	0.16	0.82	0.32	Nodiff	Alanylalanine (Ala-Ala)
M1100.pos	-0.13	0.80	0.31	Nodiff	Glu-His
M1101.pos	-0.35	0.14	1.35	Nodiff	N-Acetyl-D-galactosaminitol
M1102.pos	-0.63	0.17	1.30	Nodiff	4-[4-(4-Chlorophenyl)-1,3-thiazol-2-yl]morpholine
M1103.pos	1.74	0.26	1.14	Nodiff	2-[4-[4-(4-Amidinophenyl)piperazino]piperidino]acetic acid
M1104.pos	0.14	0.43	0.77	Nodiff	Mangiferin
M1105.neg	0.00	1.00	0.12	Nodiff	Benzamide, 4-[4-(1,3-benzodioxol-5-yl)-5-(2-pyridinyl)-1H-imidazol-2-yl]-
M1106.pos	-0.10	0.79	0.17	Nodiff	4-Amino-2-oxo-1,2-dihydropyrimidine-5-carboxylic acid
M1107.pos	0.24	0.47	0.72	Nodiff	4-Thiazolidinone, 5-[(4-ethylphenyl)methylene]-2-thioxo-
M1108.pos	-0.17	0.74	0.36	Nodiff	N-(2,5-Dimethylphenyl)-1-methyl-1H-pyrazolo[3,4-d]pyrimidin-4-amine
M1109.pos	-0.07	0.70	0.44	Nodiff	Phenylalanyl-Glycine
M111.neg	0.37	0.25	1.20	Nodiff	Pyroglutamic acid
M1110.pos	0.84	0.18	1.26	Nodiff	1-O-Hexadecyl-2-O-(2E-butenoyl)-sn-glyceryl-3-phosphocholine
M1111.neg	-0.41	0.48	0.65	Nodiff	6-(Ethylsulfonyl)-2H-1,4-benzoxazin-3(4H)-one
M1112.pos	-0.07	0.51	0.71	Nodiff	4-Methoxy-5-(3,7,11,15-tetramethyl-2,6,10,14-hexadecatetraenyl)-1,3-benzenediol
M1113.pos	-1.99	0.25	1.10	Nodiff	Betanidin
M1114.pos	-0.25	0.41	0.73	Nodiff	2-Amino-6-ethyl-4(3H)-pyrimidinone
M1115.neg	-1.18	0.15	1.41	Nodiff	Propanil
M1116.neg	0.15	0.45	0.83	Nodiff	1-Mercapto[1,2,4]triazolo[4,3-a]quinoxalin-4(5H)-one
M1117.pos	0.62	0.07	1.58	Nodiff	Anabsinthin
M1118.neg	-0.18	0.46	0.79	Nodiff	1-Hydroxy-2-naphthoic acid
M1119.pos	2.07	0.12	1.52	Nodiff	Gatifloxacin
M112.neg	0.84	0.12	1.47	Nodiff	Glucose
M1120.pos	0.00	0.46	0.73	Nodiff	N-Phenyl-4-(3-pyridinyl)-2-pyrimidinamine
M1121.pos	-0.46	0.52	0.71	Nodiff	3-{[4-(Trifluoromethyl)phenyl]amino}cyclohex-2-en-1-one
M1122.pos	-0.42	0.44	0.77	Nodiff	Pipemidic acid
M1123.pos	0.14	0.43	0.78	Nodiff	Valyl-Glycine
M1124.pos	0.28	0.59	0.59	Nodiff	Prolyl-Serine
M1125.neg	0.28	0.53	0.74	Nodiff	1,6-ANHYDRO-B-GLUCOSE
M1126.pos	-0.66	0.22	1.17	Nodiff	19(R)-hydroxy-PGE2
M1127.pos	0.72	0.20	1.31	Nodiff	2,3-Dihydro-2,3-dihydroxy-9-phenyl-1H-phenalen-1-one
M1128.pos	0.33	0.39	0.87	Nodiff	Thiamylal
M1129.neg	-0.08	0.37	0.90	Nodiff	3-Phosphonopropanoic acid
M113.pos	-0.07	0.99	0.03	Nodiff	Valine
M1130.neg	-0.43	0.97	0.03	Nodiff	9-(Methoxycarbonyl)dec-9-enoic acid
M1131.pos	-0.96	0.81	0.25	Nodiff	Flubendazole
M1132.neg	1.31	0.29	1.10	Nodiff	Ikarugamycin
M1133.neg	0.40	0.91	0.09	Nodiff	2,2'-[(Phosphonomethyl)imino]diacetic acid

M1134.pos	0.40	0.42	0.85	Nodiff	N.alpha.-Benzoyl-Asn-Gly-Thr-amide
M1135.neg	1.18	0.33	0.98	Nodiff	2-Hydroxy-N-(4-methylphenyl)-5-nitrobenzamide
M1136.pos	0.43	0.21	1.23	Nodiff	4-Methoxy-N-(3-pyridinylmethyl)benzenesulfonamide
M1137.pos	-0.18	0.64	0.52	Nodiff	3-Nitrophenylhydrazine
M1138.pos	-0.40	0.65	0.44	Nodiff	12,13-Dihydroxy-9Z-octadecenoic acid
M1139.neg	-0.29	0.65	0.48	Nodiff	3-(Hepta-1,3-dienyl)hexanedioic acid
M114.pos	0.39	0.27	1.08	Nodiff	Pyrraline
M1140.pos	-0.22	1.00	0.08	Nodiff	1-Monolinolenin
M1141.neg	1.30	0.11	1.53	Nodiff	2,4(1H,3H)-Quinazolinedithione
M1142.pos	0.22	0.96	0.02	Nodiff	Cellopentaose
M1143.pos	-0.62	0.05	1.67	Nodiff	Lysyl-Leucine
M1144.pos	0.41	0.39	0.92	Nodiff	4-(3-Pyridinyl)-2-pyrimidinamine
M1145.pos	0.26	0.40	0.90	Nodiff	9H-Carbazole, 2-[(2E)-2-(1-azabicyclo[2.2.2]oct-3-ylidene)-2-fluoroethoxy]-
M1146.neg	1.04	0.06	1.69	Nodiff	2-(1H-Pyrrol-1-yl)-5,6-dihydro-4H-cyclopenta[b]thiophene-3-carboxylic acid
M1147.neg	1.02	0.27	1.14	Nodiff	2-Hydroxy-N-{2-[(2-hydroxybenzoyl)amino]ethyl}benzamide
M1148.neg	-0.45	0.31	0.93	Nodiff	(1S,3R)-1-Aminocyclopentane-1,3-dicarboxylic acid
M1149.neg	0.11	0.58	0.62	Nodiff	3-Cyclohexyl-2-sulfanyl-4(3H)-quinazolinone
M115.pos	0.35	0.48	0.77	Nodiff	Trigonelline
M1151.neg	0.33	0.40	0.84	Nodiff	6-(4-Morpholinyl)-1H-purine
M1152.pos	0.17	0.40	0.81	Nodiff	2-Amino-4,5,6,7-tetrahydro-1-benzothiophene-3-carboxylic acid
M1153.pos	0.03	0.88	0.17	Nodiff	Toddalolactone
M1154.pos	0.26	0.34	0.94	Nodiff	2-Chloro-N-(4-methoxyphenyl)nicotinamide
M1155.pos	-0.45	0.52	0.69	Nodiff	2,4,6-Trimethoxypyrimidine
M1156.pos	-0.46	0.61	0.47	Nodiff	Methanone, [1-(5-fluoropentyl)-1H-indol-3-yl](2,2,3,3-tetramethylcyclopropyl)-
M1157.pos	0.29	0.41	0.81	Nodiff	(1-Methyl-1H-imidazol-2-yl)(2-thienyl)methanol
M1158.pos	-0.29	0.48	0.72	Nodiff	2-[(6-Ethoxy-4-methyl-2-quinazolinyl)amino]-5,6-dihydro-4-pyrimidinol
M1159.pos	-0.04	0.92	0.10	Nodiff	2-(Dimethylamino)-1,3-benzothiazol-6-ol
M116.pos	0.45	0.26	1.13	Nodiff	Methionine
M1160.pos	-0.89	0.71	0.34	Nodiff	2-Amino-1-(4-aminophenyl)-1H-pyrrolo[2,3-b]quinoxaline-3-carbonitrile
M1161.neg	-0.51	0.66	0.45	Nodiff	6-(1-Pyrrolidinyl)-1H-purine
M1162.pos	-0.59	0.09	1.61	Nodiff	4-[(Ethylamino)carbonylamino]benzoic acid
M1163.pos	0.33	0.38	0.95	Nodiff	1-Deoxy-D-glucitol
M1164.neg	0.55	0.81	0.32	Nodiff	2-(6-Ethenyl-9-hydroxy-2,6,9a-trimethyl-4-oxo-3,5,5a,7,8,9-hexahydro-2H-pyrano[2,3-b]chromen-7-yl)-2-methylpropanoic acid
M1165.neg	-0.40	0.75	0.33	Nodiff	Methyl 6-(acetyloxy)-7,11-dihydroxy-1-oxoabieta-8,11,13-trien-18-oate
M1166.neg	0.64	0.38	0.96	Nodiff	2,4-Dichloro-5-ethyl-3-methylphenol
M1167.pos	-1.05	0.02	1.82	Nodiff	N-(2-Furylmethyl)-N'-(2-methoxyphenyl)carbamimidothioic acid
M1168.pos	0.65	0.18	1.31	Nodiff	1,4,10-Trioxa-7,13-diazacyclopentadecane, 7,13-bis[(4-nitrophenyl)methyl]-
M1169.pos	0.37	0.39	0.92	Nodiff	2,2'-(4-(2-Hydroxyethylamino)-3-nitrophenylazanediyl)diethanol
M117.pos	0.06	0.95	0.13	Nodiff	Betaine
M1170.neg	0.87	0.34	0.98	Nodiff	2H-1-Benzopyran-2-one, 7-mercapto-4-methyl-

M1171.neg	-0.44	0.32	1.01	Nodiff	5-(9-Anthrylmethylene)-2,4,6(1H,3H,5H)-pyrimidinetrione
M1172.pos	-0.91	0.02	1.92	Nodiff	Mefloquine
M1173.pos	1.42	0.00	2.17	Nodiff	Clomipramine
M1174.pos	-0.64	0.94	0.01	Nodiff	L-alpha-glutamyl-L-hydroxyproline
M1175.neg	-0.41	0.18	1.40	Nodiff	Glu-Val
M1176.pos	-0.14	0.62	0.58	Nodiff	3-{2-[2-(2-Cyanoethoxy)ethoxy]ethoxy}propanenitrile
M1177.neg	-0.35	0.34	0.90	Nodiff	1H-Indole-3-carboxamide, 1-(5-fluoropentyl)-N-1-naphthalenyl-
M1178.neg	-0.09	0.74	0.30	Nodiff	Bisphenol AP
M1179.neg	-0.73	0.22	1.10	Nodiff	Norlichexanthone
M118.pos	0.15	0.58	0.61	Nodiff	Norleucine
M1180.pos	0.48	0.38	0.93	Nodiff	1-Ethyl-3-methylimidazolium cation
M1181.pos	-0.51	0.05	1.69	Nodiff	2-Bromo-4,6-diphenylnicotinonitrile
M1182.pos	0.34	0.55	0.58	Nodiff	Ganodosterone
M1183.neg	0.15	0.96	0.08	Nodiff	6-Bromo-4(1H)-quinazolinone
M1184.pos	0.12	0.45	0.82	Nodiff	Serpentine cation
M1185.neg	-0.01	0.47	0.74	Nodiff	Acacetin-7-O-rutinoside
M1186.pos	0.95	0.19	1.32	Nodiff	N6-Acetyl-5S-hydroxy-L-lysine
M1187.pos	0.62	0.67	0.48	Nodiff	5-[3-(2-Methoxyphenyl)-1H-pyrrolo[2,3-b]pyridin-5-yl]-N,N-dimethylnicotinamide
M1188.pos	0.33	0.51	0.73	Nodiff	3-[(5,6-Diphenylfuro[2,3-d]pyrimidin-4-yl)amino]-1-propanol
M1189.pos	-1.00	0.66	0.38	Nodiff	3-Methyl-.gamma.-butyrolactone
M119.pos	0.15	0.58	0.61	Nodiff	Leucine
M1190.pos	0.59	0.37	0.97	Nodiff	2,4-Quinazolinediamine, N2,N4-bis(phenylmethyl)-
M1191.neg	0.49	0.48	0.81	Nodiff	N-(Naphthalen-2-yl)-2-sulfanylacetamide
M1192.pos	-0.69	0.11	1.46	Nodiff	N2-Benzyl-1,3,5-triazine-2,4-diamine
M1193.pos	-0.13	0.99	0.03	Nodiff	D-Mannosamine
M1194.pos	-0.32	0.22	1.28	Nodiff	Octyl gallate
M1195.neg	0.15	0.45	0.81	Nodiff	Propachlor ESA
M1196.pos	-0.80	0.06	1.67	Nodiff	6,7-Dimethoxy-2-phenylquinoxaline
M1197.pos	0.23	0.50	0.66	Nodiff	2-(4-Methyl-5-thiazolyl)ethyl_isobutyrate
M1198.pos	0.36	0.40	0.91	Nodiff	Ethyl 2-amino-5-ethyl-3-thiophenecarboxylate
M1199.neg	0.00	0.86	0.08	Nodiff	L-Erythrulose
M12.neg	0.79	0.35	0.97	Nodiff	4-Methylquinolin-2-ol
M120.neg	1.90	0.08	1.62	Nodiff	Trp-Gly
M1200.neg	-0.56	0.25	1.16	Nodiff	3-Pyridinecarbonitrile, 1,2-dihydro-4-methyl-2-thioxo-6-(trifluoromethyl)-
M1201.neg	0.50	0.27	1.18	Nodiff	5-Chloro-N-(3-cyanophenyl)-2-hydroxybenzamide
M1202.neg	-1.06	0.75	0.27	Nodiff	Tricin methyl ether
M1203.neg	-0.35	0.46	0.72	Nodiff	Met-Asp
M1204.neg	-0.23	0.49	0.77	Nodiff	6-Amino-2,4-dichloro-3-ethylphenol
M1205.pos	-0.63	0.38	0.96	Nodiff	DG(18:4(6Z,9Z,12Z,15Z)/18:1(11Z)/0:0)
M1206.pos	0.50	0.33	0.95	Nodiff	5-(2-Furyl)-4H-1,2,4-triazole-3-thiol
M1207.pos	-0.04	0.88	0.19	Nodiff	.beta.-Alanine, N-[2-(2-pyridinyl)-6-(1,2,4,5-tetrahydro-3H-3-benzazepin-3-yl)-4-pyrimidinyl]-
M1209.pos	-0.26	0.54	0.69	Nodiff	2-(4-Methylpiperidin-1-yl)-5-(trifluoromethyl)aniline
M121.neg	3.13	0.09	1.64	Nodiff	Thr-Leu
M1210.pos	-0.66	0.29	0.97	Nodiff	Danofloxacin
M1211.neg	-0.56	0.07	1.61	Nodiff	Ser-His
M1212.pos	-0.28	0.29	1.09	Nodiff	N-Butyl-1,2-benzisothiazol-3-amine 1,1-dioxide
M1213.pos	-1.29	0.03	1.93	Nodiff	4-[(3,5-Dimethyl-1H-pyrazole-4-)sulfonyl]morpholine
M1214.neg	1.10	0.13	1.54	Nodiff	N-(1-Phenyl-3,6-diazatricyclo[4.3.1.1~3,8~]undec-9-yl)thiourea

M1215.pos	0.53	0.34	1.00	Nodiff	Pentostatin
M1216.neg	0.55	0.29	1.12	Nodiff	(-)-Catechin
M1217.neg	1.18	0.17	1.43	Nodiff	6-Methylmercaptopurine
M1218.pos	0.76	0.39	0.86	Nodiff	7-Methoxy-1-methyl-2-phenyl-4(1H)-quinolinone
M1219.pos	-0.72	0.12	1.39	Nodiff	4-Methoxy-3-geranylgeranyl-1,2-dihydroxybenzene
M122.neg	0.50	0.42	0.77	Nodiff	Allocholic acid
M1220.pos	0.71	0.32	1.04	Nodiff	Tiotropium
M1221.pos	-0.88	0.06	1.58	Nodiff	Isothipendyl
M1222.pos	0.06	0.88	0.20	Nodiff	6,7-dimethoxy-2,2-dimethyl-2h-1-benzopyran
M1223.pos	0.34	0.37	0.86	Nodiff	3-[Hexadecyl(dimethyl)ammonio]-1-propanesulfonate
M1224.pos	0.40	0.30	1.02	Nodiff	1-O-Hexadecyl-2-O-acetyl-sn-glyceryl-3-phosphorylcholine
M1225.neg	-0.17	0.38	0.86	Nodiff	N-Stearoyltaurine
M1226.pos	0.43	0.36	0.91	Nodiff	Quinaprilat
M1227.pos	1.31	0.16	1.40	Nodiff	PA(22:0/20:5(5Z,8Z,11Z,14Z,17Z))
M1228.pos	-0.08	0.79	0.28	Nodiff	Chrysin
M1229.neg	-0.11	0.73	0.22	Nodiff	2-Bromo-4,6-dichlorophenol
M123.neg	0.18	0.58	0.62	Nodiff	3-Hydroxybutyric acid
M1230.pos	-0.05	0.45	0.72	Nodiff	Lys-Val
M1231.pos	0.44	0.44	0.82	Nodiff	Adapalene
M1232.neg	-1.22	0.07	1.63	Nodiff	3-[(Methylsulfonyl)amino]benzoic acid
M1233.neg	0.31	0.53	0.55	Nodiff	1-(3,4,5-Trimethoxyphenyl)-1,2,3,4-tetrahydro-6,7-isoquinolinediol
M1234.pos	1.00	0.34	0.95	Nodiff	3-Cyclopropyl-1-methyl-1H-pyrazol-5-amine
M1235.pos	0.43	0.19	1.31	Nodiff	6-Ethyl-7-hydroxy-3-phenyl-2-(trifluoromethyl)-4H-chromen-4-one
M1236.neg	1.06	0.66	0.58	Nodiff	4-Hydroxy-3-[1-(5-hydroxy-2,6,6-trimethyltetrahydro-2H-pyran-2-yl)ethyl]-2(1H)-quinolinone
M1237.neg	0.39	0.61	0.65	Nodiff	Thr-Gly-Thr
M1238.pos	-0.40	0.65	0.50	Nodiff	3-Hydroxy-carbofuran
M1239.pos	0.23	0.22	1.17	Nodiff	6-Nitrocoumarin-3-carboxylic acid
M124.pos	0.25	0.33	1.02	Nodiff	1-O-Hexadecyl-sn-glycero-3-phosphocholine (LPC(O-16:0/0:0))
M1240.pos	-0.14	0.50	0.73	Nodiff	Pentoxifylline
M1241.neg	-0.22	0.62	0.39	Nodiff	4,4-Dimethyl-L-glutamic acid
M1242.pos	0.25	0.43	0.85	Nodiff	Phenol, 3-[[6-(3-aminophenyl)-7H-pyrrolo[2,3-d]pyrimidin-4-yl]oxy]-
M1243.neg	-0.16	0.73	0.34	Nodiff	(-)-Epigallocatechin
M1244.pos	-0.35	0.42	0.82	Nodiff	3-Hexaprenyl-4,5-Dihydroxybenzoic_acid
M1245.pos	-0.35	0.92	0.11	Nodiff	N-(o-Hydroxyphenyl)naphthalimide
M1246.neg	-0.35	0.96	0.17	Nodiff	(3E)-4-(1-Hydroxy-2,6,6-trimethyl-4-oxocyclohex-2-en-1-yl)but-3-en-2-yl 6-O-pentopyranosylhexopyranoside
M1247.pos	0.17	0.44	0.83	Nodiff	2,2'-(3-(Trifluoromethyl)phenylazanediyl)diethanol
M1248.pos	0.79	0.19	1.31	Nodiff	Geniposidic_acid
M1249.pos	0.00	0.99	0.03	Nodiff	6,7-Dihydro-5H-cyclopenta[d]pyrimidine-2,4-diamine
M125.pos	0.75	0.17	1.40	Nodiff	Leu-Ile
M1250.pos	-0.01	0.90	0.18	Nodiff	1-(Pyrimidin-2-yl)-1,4-diazepane
M1251.pos	-0.26	0.41	0.75	Nodiff	Schleicherastatin_6
M1252.neg	-0.19	0.76	0.38	Nodiff	Methyl 4-formyl-3-hydroxy-2-naphthoate
M1253.neg	-1.11	0.71	0.39	Nodiff	Cholic acid/Muricholic acid, tryptophan-conjugated
M1254.neg	0.43	0.25	1.13	Nodiff	4-Allyl-5-(2-methylphenyl)-4H-1,2,4-triazol-3-yl hydrosulfide
M1255.pos	0.98	0.20	1.31	Nodiff	Dulxanthone_C

M1256.neg	-0.86	0.18	1.22	Nodiff	Rhaponticin
M1257.neg	0.79	0.07	1.63	Nodiff	Gly-Pro-Glu
M1258.neg	0.75	0.66	0.46	Nodiff	.beta.-Peltatin
M126.pos	-0.64	0.77	0.28	Nodiff	Deoxyinosine
M1260.neg	-0.38	0.23	1.20	Nodiff	Belinostat
M1261.pos	0.27	0.64	0.48	Nodiff	Avenic_acid_B
M1262.neg	-0.46	0.55	0.71	Nodiff	2,4(1H,3H)-Pyrimidinedione, 6-[4-(3-chlorophenyl)-1-piperazinyl]-3-cyclohexyl-
M1263.pos	-0.86	0.33	1.05	Nodiff	5,6,16-Trihydroxygrayanotox-10-en-3-yl hexopyranoside
M1264.pos	-0.39	0.09	1.59	Nodiff	2-Mercaptomethylbenzimidazole
M1265.neg	0.98	0.03	1.85	Nodiff	2-(2-oxo-8,9-dihydrofuro[2,3-h]chromen-8-yl)propan-2-yl acetate
M1266.pos	0.91	0.34	0.95	Nodiff	1-(3-Nitrophenyl)-3-(4-pyridylmethyl)pyrido[2,3-d]pyrimidine-2,4-dione
M1267.pos	0.04	0.55	0.66	Nodiff	Methyl 4-(9H-purin-6-ylamino)benzoate
M1268.pos	0.18	0.46	0.74	Nodiff	Prostaglandin F2.alpha. 1,15-lactone
M1269.neg	0.35	0.70	0.35	Nodiff	(1.alpha.,2.alpha.,3.beta.,5.Xi.,9.Xi.,13.Xi.,14.Xi.,18.Xi.)-1,2,3,22,23,29-Hexahydroxy-13,27-cycloolean-11-en-28-oic acid
M127.neg	0.35	0.61	0.57	Nodiff	Tauro-beta-muricholic acid
M1270.neg	0.36	0.34	1.02	Nodiff	2-Chloro-1-(5-chloro-2-methyl-1H-indol-3-yl)ethanone
M1271.pos	0.32	0.59	0.44	Nodiff	(3S,3'R,5R,6R)-7',8'-Didehydro-3,6-epoxy-5,6-dihydro-beta,beta-carotene-3',5-diol
M1272.pos	-0.07	0.50	0.65	Nodiff	Santinamide cation
M1273.pos	-0.20	0.98	0.06	Nodiff	N-(2,6-Dimethylphenyl)-N'-phenylthiourea
M1274.pos	0.96	0.18	1.34	Nodiff	Isopropalin
M1275.pos	0.62	0.89	0.05	Nodiff	Orteronel
M1276.pos	1.11	0.27	1.08	Nodiff	Seneciphylline N-oxide
M1277.neg	-1.16	0.06	1.63	Nodiff	1,3,4,5-Tetrahydrothiopyrano[4,3-b]indole
M1278.neg	0.31	0.35	1.00	Nodiff	Farrerol
M1279.pos	0.89	0.04	1.84	Nodiff	1h-indole-3-butanoic acid
M128.neg	-0.36	0.50	0.77	Nodiff	2-Hydroxyethanesulfonic acid
M1280.pos	0.04	0.99	0.07	Nodiff	4-Acetamido-2-aminobutanoic_acid
M1281.pos	0.17	0.42	0.78	Nodiff	4-Amino-1-methyl-5H-chromeno[3,4-c]pyridin-5-one
M1282.pos	-0.10	0.45	0.85	Nodiff	Flunitrazepam
M1283.pos	-0.70	0.05	1.70	Nodiff	2-[(1E)-2-(4-Methoxyphenyl)ethenyl]-4(3H)-quinazolinone
M1284.pos	0.35	0.95	0.03	Nodiff	Lysine conjugated deoxycholic acid putative
M1286.neg	-0.87	0.86	0.11	Nodiff	2-Hydroxy-2',3'-dichlorobiphenyl
M1287.pos	-0.19	0.93	0.11	Nodiff	Alprazolam
M1288.neg	0.15	0.43	0.88	Nodiff	2,4-Bis(trifluoromethyl)pyrimidine-5-carboxylic acid
M1289.pos	0.39	0.39	0.92	Nodiff	1-(3-Chlorophenyl)-3-(4-sulfamoylphenyl)urea
M129.neg	0.36	0.44	0.74	Nodiff	Galactono-1,4-lactone
M1290.neg	-0.15	0.40	0.79	Nodiff	2-[(4S,5S,5aS,9aS)-4-Methoxy-6,6,9a-trimethyl-5-[(2E,4E,6E)-octa-2,4,6-trienoyl]oxy-1-oxo-4,5,5a,7,8,9-hexahydro-3H-benzo[e]isoindol-2-yl]pentanedioic acid
M1291.neg	-0.15	0.42	0.82	Nodiff	5-{[2-(4-Chlorophenyl)ethyl]amino}-5-oxopentanoic acid
M1292.neg	0.92	0.19	1.37	Nodiff	2-[(2-Oxo-2-phenylethyl)carbamoyl]benzoic acid
M1293.neg	-0.12	0.87	0.15	Nodiff	2-Naphthalenethiol
M1294.neg	-0.35	0.59	0.55	Nodiff	1-(2,4-Dihydroxyphenyl)-2-(4-fluorophenoxy)ethanone
M1295.pos	-0.54	0.11	1.54	Nodiff	4-Amino-6,8-difluoro-2-methylquinoline
M1296.pos	0.76	0.19	1.32	Nodiff	1H-Pyrano[3',4':6,7]indolizino[1,2-b]quinoline-3,14(4H,12H)-dione,

					4,11-diethyl-4,9-dihydroxy-, (4S)-
M1297.pos	-0.07	0.74	0.40	Nodiff	Tetraethylene glycol monomethyl ether
M1298.neg	-1.13	0.74	0.39	Nodiff	5-[(Chloroacetyl)amino]-2-hydroxybenzoic acid
M1299.pos	-0.10	0.44	0.86	Nodiff	Dextrorphan O-.beta.-D-glucuronide
M13.neg	0.21	0.50	0.62	Nodiff	Hyocholic acid
M130.neg	-0.03	0.98	0.09	Nodiff	12-Hydroxystearic acid
M1300.pos	0.32	0.41	0.87	Nodiff	6-Acetylcodeine
M1301.neg	-0.50	0.26	1.03	Nodiff	9-Nitro-3a,4,5,9b-tetrahydro-3H-cyclopenta [c]quinoline-4,6-dicarboxylic acid
M1302.neg	1.11	0.03	1.86	Nodiff	6.alpha./.beta.-Hydroxyoxymorphone
M1303.pos	-0.16	0.47	0.69	Nodiff	Propafenone
M1304.pos	-0.39	0.21	1.29	Nodiff	(3-Ethyl-2-imino-2,3-dihydro-1H-benzimid azol-1-yl)acetic acid
M1305.pos	0.67	0.35	0.95	Nodiff	Methylcodeine
M1307.neg	0.16	0.60	0.56	Nodiff	Midafotel
M1308.pos	1.74	0.05	1.71	Nodiff	1,3,5-Tris(4-hydroxyphenyl)benzene
M1309.pos	0.31	0.13	1.36	Nodiff	5-Chloro-2-(chloromethyl)-1,3-benzoxazole
M131.pos	-0.74	0.33	1.07	Nodiff	N-(3-Methylbut-2-EN-1-YL)-9H-purin-6-a mine
M1310.pos	1.04	0.32	1.05	Nodiff	L-Pyridosine
M1311.neg	0.02	0.88	0.11	Nodiff	1H-3-Benzazepine-7,8-diol, 6-chloro-2,3,4,5-tetrahydro-1-phenyl-
M1312.neg	0.48	0.30	1.07	Nodiff	[4]-Gingerdiol_3,5-diacetate
M1313.pos	-0.13	0.48	0.76	Nodiff	Setariol
M1314.pos	0.10	0.44	0.83	Nodiff	23S,25-dihydroxyvitamin_D3
M1315.neg	-0.14	0.50	0.77	Nodiff	3a,4,5,9b-Tetrahydro-3H-cyclopenta[c]quin oline-4,8-dicarboxylic acid
M1316.neg	0.26	0.47	0.68	Nodiff	(1R)-4-(3-Hydroxybutyl)-3,5,5-trimethylcyc lohex-3-en-1-yl 6-O-.beta.-D-glucopyranosyl-.beta.-D-gluco pyranoside
M1317.pos	-0.01	0.82	0.20	Nodiff	Lasmiditan
M1318.neg	0.24	0.40	0.95	Nodiff	tras-4-Hydroxycinnamic acid sulfate
M1319.pos	0.85	0.17	1.28	Nodiff	Desmethyldoxepin
M132.neg	0.51	0.35	0.88	Nodiff	Palmitic acid
M1320.neg	-0.65	0.82	0.18	Nodiff	2,3-Dimethylbenzene-1-sulfonic acid
M1321.pos	0.28	0.54	0.71	Nodiff	Flutamide
M1322.neg	-0.85	0.88	0.15	Nodiff	2,2'-(1H-1,2,4-Triazole-3,5-diyl)diphenol
M1323.neg	-0.22	0.47	0.81	Nodiff	Methyl 3-hydroxy-4-nitrobenzoate
M1324.neg	-1.05	0.06	1.66	Nodiff	2,2',4,4'-Tetrahydroxybenzophenone
M1325.pos	-0.53	0.10	1.54	Nodiff	5-(2-Chlorophenyl)-4-pyrimidinamine
M1326.neg	0.80	0.23	1.11	Nodiff	2-Deoxyribose 5-phosphate
M1327.neg	0.94	0.22	1.24	Nodiff	2-Chloro-4,5-dimethoxybenzoic acid
M1328.pos	0.41	0.39	0.91	Nodiff	N,N-Dimethylhistidine
M1329.neg	1.32	0.29	1.16	Nodiff	Methyl 3-(acetyloxy)-16,19-dihydroxy-4,4,8,12,16-p entamethyl-15,17-dioxoandrost-11-ene-14-c arboxylate
M133.neg	0.10	0.95	0.09	Nodiff	Stearic acid
M1330.pos	0.73	0.92	0.18	Nodiff	7-Amino-4-methylquinolin-2-ol
M1331.pos	0.41	0.33	0.89	Nodiff	2-Iminobiotin
M1332.pos	-0.09	0.66	0.44	Nodiff	Mianserin
M1333.pos	-1.50	0.19	1.19	Nodiff	11-[(3-Hydroxypropyl)amino]-2,3-dihydro-1H-cyclopenta[4,5]pyrido[1,2-a]benzimida zole-4-carbonitrile
M1334.neg	-0.01	0.88	0.19	Nodiff	4-Hydroxy-2-(trifluoromethyl)thiophene
M1335.pos	0.42	0.39	0.92	Nodiff	2-Cyclopropyl-1H-indole
M1336.pos	-0.02	0.91	0.17	Nodiff	Tetrahydrozoline
M1337.neg	0.25	0.24	1.15	Nodiff	trans-pterostilbene
M1338.pos	-1.00	0.10	1.55	Nodiff	2-Methyl-.alpha.-pyrrolidinobutiophenone
M1339.pos	0.59	0.37	0.95	Nodiff	N-Isopropyl-2-phenyl-4-quinolinecarboxa mide
M134.neg	-0.45	0.86	0.22	Nodiff	Docosahexaenoic acid (DHA)

M1340.pos	-0.35	0.75	0.32	Nodiff	Isonicoteine
M1341.pos	0.51	0.35	0.93	Nodiff	(6aR,12bR)-4,6,6a,7,8,12b-Hexahydro-7-methylindolo[4,3-ab]phenanthridine
M1342.pos	1.57	0.02	1.92	Nodiff	Prexanthoperol
M1343.pos	0.21	0.51	0.73	Nodiff	Cocaethylene
M1344.pos	-0.29	0.32	0.93	Nodiff	(3S,4S,4aR,6R,11bS,11cS)-11c-Ethenyl-2-methyl-1,2,3,4,4a,5,6,11c-octahydro-6,4-(epoxymethano)-3,11b-methanopyrido[4,3-c]carbazole
M1345.neg	1.46	0.20	1.31	Nodiff	[4-[(E)-2-(1H-Indazol-3-yl)vinyl]phenyl]piperazinomethanone
M1346.pos	-0.36	0.38	0.90	Nodiff	2-(4-tert-Butylphenyl)benzimidazole
M1347.pos	-1.72	0.74	0.36	Nodiff	Dolasetron
M1348.pos	-0.41	0.44	0.66	Nodiff	Rosmaricine
M1349.neg	0.14	0.61	0.53	Nodiff	1-Phenyl-1H-pyrazolo[3,4-d]pyrimidine-4-thiol
M135.pos	-0.36	0.94	0.03	Nodiff	Cer(d18:1/16:0)
M1350.pos	0.73	0.17	1.28	Nodiff	1,7'-Dimethyl-2'-propyl-1H,1'H-2,5'-bibenzo[d]imidazole
M1351.pos	-0.45	0.09	1.52	Nodiff	4-Amino-2,6-diphenyl-5-pyrimidinecarbonitrile
M1352.neg	0.69	0.35	0.94	Nodiff	3-[(4-Fluorobenzoyl)amino]benzoic acid
M1353.pos	-0.41	0.19	1.26	Nodiff	trans-4-[4-(Dimethylamino)styryl]-1-methylpyridinium cation
M1354.neg	-0.43	1.00	0.05	Nodiff	6-(4-Fluorophenyl)-3(2H)-pyridazinone
M1355.pos	-0.40	0.53	0.68	Nodiff	Dihydroberberine
M1356.neg	0.03	1.00	0.10	Nodiff	N-(4-Phenoxyphenyl)-2-pyrazinecarboxamide
M1357.pos	0.57	0.16	1.41	Nodiff	2-Amino-6-(4-methoxyphenyl)-4-phenylnicotinonitrile
M1358.pos	-0.71	0.44	0.77	Nodiff	Sinomenine
M1359.pos	-0.33	0.31	0.99	Nodiff	6-Methyl-2-phenyl-4-quinolinecarboxylic acid
M136.neg	0.54	0.22	1.29	Nodiff	N-Methyl-L-asparagine
M1360.pos	0.65	0.22	1.19	Nodiff	2-Hexyl-5-[2-(4-hydroxy-3-methoxyphenyl)ethyl]furan
M1361.pos	0.57	0.43	0.74	Nodiff	3'-Hydroxystanozolol
M1362.pos	-1.63	0.46	0.84	Nodiff	N,N-Diethylcathinone
M1363.pos	0.11	0.58	0.53	Nodiff	Codeine
M1364.neg	0.10	0.43	0.82	Nodiff	(Z)-4-(4-Hydroxybenzylidene)-2-phenyloxazol-5(4H)-one
M1365.pos	0.54	0.36	0.96	Nodiff	7-Methoxy-1,2,3,4-tetrahydroacridin-9-amine
M1366.neg	0.30	0.52	0.71	Nodiff	N-(4-Anilinophenyl)isonicotinamide
M1367.pos	-0.02	0.95	0.07	Nodiff	7-Methylnaphthalen-2-ol
M1368.pos	0.41	0.43	0.74	Nodiff	Morphine
M1369.neg	-0.36	0.13	1.38	Nodiff	7-Hydroxyflavanone
M137.neg	1.06	0.11	1.56	Nodiff	Leu-Val
M1370.pos	-1.09	0.01	1.94	Nodiff	2-Amino-1-(4-methoxyphenyl)-4-phenyl-1H-pyrrole-3-carbonitrile
M1371.pos	0.20	0.42	0.77	Nodiff	Avenalumin_II
M1372.neg	-1.94	0.14	1.47	Nodiff	4-(3-Bromopyrazolo[1,5-a]pyrimidin-6-yl)phenol
M1373.pos	-1.10	0.60	0.50	Nodiff	N-[(3R)-Quinuclidin-3-yl]furo[2,3-c]pyridine-5-carboxamide
M1374.pos	1.36	0.09	1.54	Nodiff	Seneciphyllin
M1375.pos	0.23	0.42	0.78	Nodiff	2-(Piperidin-1-yl)benzo[d]oxazole
M1376.pos	0.40	0.23	1.25	Nodiff	3-Amino-1,1,1-trifluoro-2-propanol
M1377.pos	0.21	0.49	0.70	Nodiff	2-Chloro-5,6,7,8-tetrahydroquinoxaline
M1378.neg	0.19	0.67	0.50	Nodiff	4-Hydroxy-2',4',6'-trimethoxychalcone
M1379.pos	-0.69	0.04	1.72	Nodiff	Etaqualone
M138.neg	-1.37	0.24	1.14	Nodiff	2-Hydroxyhexanoic acid
M1380.pos	-0.24	0.87	0.21	Nodiff	3-Acetyl-7-diethylaminocoumarin
M1381.pos	-0.74	0.41	0.83	Nodiff	4-(Trifluoromethyl)pyridine

M1382.pos	0.66	0.38	0.87	Nodiff	Phthalic anhydride
M1383.neg	0.18	0.42	0.84	Nodiff	(3E)-3-(4-Hydroxybenzylidene)-1,3-dihydro-2H-indol-2-one
M1384.pos	1.64	0.00	2.20	Nodiff	Nalbuphine
M1385.pos	-0.19	0.61	0.45	Nodiff	Furanogermenone
M1386.pos	3.77	0.08	1.56	Nodiff	Pyridine, 4-[5-(4-butylcyclohexyl)-1,2,4-oxadiazol-3-yl]-
M1387.pos	0.73	0.20	1.23	Nodiff	1-(3-Chlorophenyl)piperazine
M1388.neg	-0.64	0.85	0.15	Nodiff	2-Propenamide, 3-(4-chlorophenyl)-N-(3-methoxyphenyl)-
M1389.neg	-0.02	0.96	0.12	Nodiff	7-Hydroxy-5-methylflavone
M139.neg	0.13	0.69	0.40	Nodiff	Myristic acid
M1390.pos	-0.12	0.89	0.17	Nodiff	8-Chloroisoquinoline
M1391.neg	0.49	0.33	1.08	Nodiff	3,4'-Dimethoxy-2'-hydroxychalcone
M1392.neg	0.29	0.41	0.86	Nodiff	6-Ethoxy-3-(4-hydroxyphenyl)-4-methylcoumarin
M1393.pos	-0.01	0.92	0.16	Nodiff	imidazo[1,2-c]pyrimidin-5-ol
M1394.neg	0.09	0.57	0.47	Nodiff	2'-Chloro-2-hydroxy-5-methylbenzophenone
M1395.pos	-0.01	0.92	0.16	Nodiff	2-Fluoro-7H-purin-6-amine
M1396.pos	-0.12	0.80	0.30	Nodiff	7-Hydroxycoumarin-4-acetic acid
M1397.neg	0.01	0.66	0.51	Nodiff	2-[(4-Methylphenyl)amino]naphthoquinone
M1398.neg	1.54	0.03	1.93	Nodiff	2-[4-(4-Fluorophenyl)piperazin-1-yl]-6-methylpyrimidin-4(3H)-one
M1399.pos	-0.44	0.08	1.60	Nodiff	Methaqualone
M14.neg	-0.34	0.40	0.81	Nodiff	3alpha-Hydroxy-6-oxo-5alpha-cholan-24-oic acid
M140.neg	-0.38	0.44	0.85	Nodiff	Galactose 1-phosphate
M1400.pos	1.80	0.03	1.96	Nodiff	Obatoclax
M1401.pos	0.96	0.38	0.85	Nodiff	Pyroquilon
M1402.neg	-0.27	0.87	0.08	Nodiff	7-Hydroxy-3-[4-hydroxy-3-(3-methylbut-2-enyl)phenyl]-8-(3-methylbut-2-enyl)chromen-4-one
M1403.neg	-0.14	0.71	0.38	Nodiff	4'-Hydroxywarfarin
M1404.pos	0.16	0.49	0.71	Nodiff	2-Amino-1-(4-methylphenyl)-4-phenyl-1H-pyrrole-3-carbonitrile
M1405.pos	-0.43	0.13	1.42	Nodiff	4-Fluoro-.alpha.-pyrrolidinobutiophenone
M1406.neg	-1.42	0.67	0.36	Nodiff	2-Ethyl-1,4-dihydroquinolin-4-one
M1407.neg	-0.72	0.05	1.70	Nodiff	Thiopental
M1408.pos	0.16	0.45	0.82	Nodiff	1H-Pyrrolo[3,4-b]quinoline-1,9(4H)-dione, 2,3-dihydro-4-methyl-3-(2-methylpropyl)-
M1409.pos	0.33	0.41	0.80	Nodiff	N,N'-Diphenyl-p-phenylenediamine
M141.neg	0.35	0.61	0.57	Nodiff	Tauro-gamma-muricholic acid
M1410.pos	0.05	0.46	0.80	Nodiff	Mefenamic acid
M1413.pos	0.70	0.16	1.39	Nodiff	3-Caffeoyl-1,5-quinolactone
M1414.pos	0.82	0.21	1.28	Nodiff	Dienogest
M1415.pos	-0.01	0.95	0.14	Nodiff	5-(1-Methylpiperidin-2-yl)-2,3'-bipyridine
M1416.neg	0.18	0.49	0.74	Nodiff	2',4'-Dihydroxy-2-methoxychalcone
M1417.pos	-0.38	0.34	0.98	Nodiff	(3E)-3-[(4-Chlorophenyl)imino]-1,3-dihydro-2H-indol-2-one
M1418.pos	0.42	0.52	0.71	Nodiff	Methoprotryne
M1419.pos	-0.22	0.72	0.31	Nodiff	Safranine cation
M142.neg	0.07	0.51	0.71	Nodiff	Lysine
M1420.neg	-0.25	0.73	0.24	Nodiff	Flufenacet ethanesulfonic acid
M1421.pos	0.82	0.61	0.57	Nodiff	Leonurine
M1422.pos	1.40	0.11	1.56	Nodiff	Nylidrin
M1423.pos	0.83	0.61	0.59	Nodiff	6-tert-Butyl-2,3,4,9-tetrahydro-1H-carbazol-1-one
M1424.pos	-0.11	0.36	0.96	Nodiff	3,5,6-Trihydroxy-5-(hydroxymethyl)-2-methoxy-2-cyclohexen-1-one
M1425.pos	2.12	0.15	1.42	Nodiff	Methysergide
M1426.pos	-1.95	0.41	0.85	Nodiff	N-(3,4-Dihydro-3,3-dimethyl-1-isoquinolin

					yl)-L-phenylalanine
M1427.neg	0.45	0.22	1.26	Nodiff	Methyl 2-[(phenylsulfonyl)amino]benzoate
M1428.pos	-0.20	0.60	0.49	Nodiff	1-Amino-1-cyclopentanecarboxylic acid
M1429.pos	0.70	0.48	0.68	Nodiff	Ala-Met
M143.pos	0.00	0.45	0.84	Nodiff	Glu-Glu
M1430.pos	0.03	0.58	0.56	Nodiff	Soyasaponin_bg
M1431.pos	-0.40	0.72	0.37	Nodiff	Metribuzin
M1432.neg	-0.39	0.96	0.16	Nodiff	Alnusone
M1433.neg	-0.15	0.97	0.04	Nodiff	L-Thyronine
M1434.pos	0.29	0.46	0.75	Nodiff	7-Aminonimetazepam
M1435.pos	0.60	0.38	0.95	Nodiff	3-Amino-2-oxazolidinone
M1436.neg	1.98	0.11	1.46	Nodiff	Phe-Met-Arg-Phe-amide
M1437.pos	-0.50	0.75	0.43	Nodiff	Propyphenazone
M1438.pos	-0.48	0.16	1.32	Nodiff	Amobarbital
M1439.pos	-0.13	0.49	0.74	Nodiff	Triphenylamine
M144.pos	0.56	0.38	0.88	Nodiff	His-Asp
M1440.neg	0.57	0.08	1.59	Nodiff	Withaferin A
M1441.neg	-1.08	0.41	0.93	Nodiff	(E)-2-Phenylethenesulfonic acid
M1442.pos	2.91	0.09	1.65	Nodiff	4-[(2,2-Diphenylacetyl)oxy]-1,1-dimethylpiperidinium cation
M1443.neg	-0.35	0.65	0.38	Nodiff	2-Benzimidazolinone, 1-benzyl-
M1444.pos	-1.14	0.06	1.65	Nodiff	Calcipotriol
M1445.pos	0.55	0.19	1.38	Nodiff	1-(4-Methoxyphenyl)ethanone
M1446.neg	0.00	0.48	0.83	Nodiff	2,4-Dimethoxy-2'-hydroxy-5'-methylchalcone
M1447.pos	-0.69	0.28	0.99	Nodiff	7-Methoxycoumarin-4-acetic acid
M1448.pos	-0.53	0.21	1.25	Nodiff	delta-Tocotrienol
M145.neg	0.99	0.33	1.02	Nodiff	Malic acid
M1450.neg	-0.88	0.40	0.76	Nodiff	Bisphenol B
M1451.pos	-0.35	0.96	0.02	Nodiff	6,7,8-Trifluoroquinoline
M1452.pos	0.29	0.51	0.66	Nodiff	4-tert-Butyl-N-(3-quinolinyl)benzamide
M1453.pos	-0.44	0.92	0.03	Nodiff	4-Amino-5-propyl-4H-1,2,4-triazol-3-yl hydrosulfide
M1454.neg	1.30	0.17	1.35	Nodiff	2-Cyclohexyl-N-phenylacetamide
M1455.pos	0.00	0.44	0.82	Nodiff	4-Amino-5-tert-butyl-4H-1,2,4-triazole-3-thiol
M1456.neg	-1.62	0.07	1.77	Nodiff	4-Chloro-2-methylphenol
M1457.neg	-2.29	0.10	1.54	Nodiff	Phosphonic acid, P-[(3R)-3-amino-4-[(3-hexylphenyl)amino]-4-oxobutyl]-
M1458.pos	-0.06	0.83	0.22	Nodiff	7-Acetoxy-3-formylchromone
M146.neg	0.68	0.36	0.95	Nodiff	Aspartate
M1460.neg	-1.30	0.29	1.02	Nodiff	Podocarpic acid
M1461.neg	-0.03	0.83	0.21	Nodiff	2-(3-Pyridinyl)-1H-indole-3-carbaldehyde
M1462.pos	0.16	0.52	0.61	Nodiff	5-[3-(Trifluoromethyl)phenyl]furfural
M1463.neg	-0.86	0.86	0.18	Nodiff	2-(4-Methoxyphenyl)quinazolin-4-ol
M1464.neg	-0.31	0.55	0.58	Nodiff	3-PRENYL-4-HYDROXYACETOPHENONE
M1465.neg	-0.65	0.16	1.29	Nodiff	2-Chloroadenine
M1466.pos	0.25	0.47	0.69	Nodiff	4-Fluoro-7-azaindole
M1467.pos	-2.33	0.12	1.44	Nodiff	[2-[2-(3-Fluorophenyl)ethynyl]-4,6-dimethyl-3-pyridyl]amine
M1468.pos	-0.48	0.56	0.55	Nodiff	2-(2-Naphthyl)-1H-indole
M1469.pos	-0.48	0.11	1.54	Nodiff	4-Chloro-8-fluoro-5H-pyrimid[5,4-b]indole
M147.neg	0.19	0.82	0.22	Nodiff	Glycerophosphoethanolamine
M1470.pos	-0.52	0.12	1.49	Nodiff	2-Methoxy-3-methyl-9H-carbazole
M1471.pos	1.64	0.74	0.39	Nodiff	3-Methyl-4-phenyl-3-buten-2-one
M1472.neg	-3.62	0.55	0.54	Nodiff	Methyl 6-nitro-1H-indole-2-carboxylate
M1473.neg	-0.90	0.64	0.55	Nodiff	5-(4-Fluorophenyl)-1,3-oxazole-2(3H)-thione
M1474.pos	-0.25	0.52	0.70	Nodiff	11-(1-Piperazinyl)dibenzo[b,f][1,4]oxazepine
M1475.pos	0.24	0.47	0.78	Nodiff	4-Phenyl-2-naphthol
M1476.neg	0.52	0.40	0.84	Nodiff	7-Hydroxy-4-(trifluoromethyl)coumarin

M1477.neg	0.84	0.19	1.34	Nodiff	3-Nitro-6-(trifluoromethyl)pyridine-2,4-diol
M1478.neg	-0.85	0.65	0.53	Nodiff	N-(2-Cyanophenyl)methanesulfonamide
M1479.pos	1.04	0.48	0.67	Nodiff	4-[2-(1-Piperidinyl)ethoxy]benzoic acid
M148.neg	0.31	0.40	0.88	Nodiff	2-Hydroxyglutaric acid
M1480.neg	0.06	0.80	0.17	Nodiff	7-Hydroxy-3-phenoxy-2-(trifluoromethyl)-4H-chromen-4-one
M1481.pos	-0.46	0.24	1.28	Nodiff	5-Chloro-2-furoic acid
M1482.pos	0.80	0.34	1.00	Nodiff	1,9-Nonanedithiol
M1483.pos	0.59	0.34	1.01	Nodiff	Cinnzeylanol
M1484.neg	0.25	0.56	0.70	Nodiff	Maltohexaose
M1485.pos	1.04	0.18	1.34	Nodiff	Fencamine
M1486.pos	-0.68	0.11	1.48	Nodiff	Entecavir
M1487.pos	-0.13	0.43	0.88	Nodiff	3-beta-Glucosylcellotriose
M1488.neg	-0.66	0.12	1.42	Nodiff	Bindarit
M1489.pos	0.80	0.33	0.94	Nodiff	Americine
M149.neg	-0.20	0.32	0.90	Nodiff	myo-Inositol
M1490.pos	-0.70	0.91	0.21	Nodiff	1-Isoquinolinyl(phenyl)methanone
M1491.neg	0.04	0.74	0.41	Nodiff	PQQ
M1492.neg	0.29	0.40	0.88	Nodiff	7,4'-Dimethoxy-5-hydroxyisoflavone
M1493.neg	0.90	0.16	1.42	Nodiff	N-(4-Fluorobenzyl)-7H-purin-6-amine
M1494.neg	0.08	0.91	0.10	Nodiff	3-(3,4-Dimethoxyphenyl)-6-hydroxy-4-methylcoumarin
M1495.pos	-1.42	0.07	1.52	Nodiff	2-Anilinocyclohexanone
M1496.pos	1.01	0.72	0.44	Nodiff	1H-Indole-3-carboxylic acid, 1-(cyclohexylmethyl)-
M1497.pos	0.44	0.25	1.17	Nodiff	7-Diethylamino-3-(3,4-dimethoxyphenyl)coumarin
M1498.neg	-0.01	0.50	0.83	Nodiff	Neoechinulin B
M1499.pos	-0.39	0.15	1.33	Nodiff	DIMBOA-Glc
M15.neg	0.96	0.37	0.87	Nodiff	Succinyladenosine
M150.neg	-0.07	0.89	0.15	Nodiff	(2S)-4-amino-2-[(azaniumylacetyl)amino]-4-oxobutanoate
M1500.pos	1.26	0.76	0.36	Nodiff	7-(4-Hydroxy-3-methoxyphenyl)-5-methoxy-1-phenyl-3-heptanone
M1501.pos	0.41	0.31	1.00	Nodiff	Imazamethabenz-methyl
M1502.neg	0.13	0.43	0.79	Nodiff	2-Carboxybenzeneboronic acid
M1503.neg	0.23	0.55	0.70	Nodiff	2'-Hydroxy-2-methoxychalcone
M1504.neg	0.49	0.24	1.19	Nodiff	Glabridin
M1505.pos	1.36	0.07	1.74	Nodiff	Dihydroethidium
M1506.neg	-0.26	0.41	0.78	Nodiff	3,7,8-Trihydroxy-3-methyl-1,4-dihydropyrano[4,3-b]chromen-10-one
M1507.neg	-0.02	0.97	0.06	Nodiff	1-(4-Chlorophenyl)-2-[(5-methyl-4H-1,2,4-triazol-3-yl)sulfanyl]ethanone
M1508.pos	-0.54	0.17	1.26	Nodiff	6-Ketoestriol
M1509.pos	-0.92	0.36	0.99	Nodiff	3,5-Di-tert-butyl-2-hydroxybenzaldehyde
M151.pos	-0.71	0.46	0.78	Nodiff	4-Aminobutyric acid (GABA)
M1510.neg	-1.61	0.21	1.35	Nodiff	N-(Trifluoroacetyl)glycine
M1511.pos	0.25	0.48	0.71	Nodiff	3-Fluoro-9H-carbazole
M1512.pos	1.33	0.09	1.64	Nodiff	Methanone, [1-(2-hydroxypentyl)-1H-indol-3-yl](2,2,3,3-tetramethylcyclopropyl)-
M1513.pos	-0.32	0.36	0.89	Nodiff	Bisoprolol
M1514.pos	0.28	0.44	0.74	Nodiff	5-[4-(Diethylamino)phenyl]-4-ethyl-4H-1,2,4-triazol-3-yl hydrosulfide
M1515.pos	-0.02	0.46	0.83	Nodiff	Cytochalasin B
M1516.neg	1.39	0.07	1.57	Nodiff	2,3-Dioxo-5-indolinecarboxylic acid
M1517.pos	-0.88	0.02	2.00	Nodiff	6-Fluoronicotinic acid
M1518.neg	0.41	0.38	0.90	Nodiff	2-Hydroxy-6-methyl-4-(trifluoromethyl)nicotinonitrile
M1519.pos	-0.05	0.43	0.89	Nodiff	Pirbuterol
M152.neg	0.19	0.48	0.77	Nodiff	Sucrose
M1520.pos	0.60	0.20	1.21	Nodiff	4-Isopropyl-4'-methylchalcone
M1521.neg	-1.55	0.79	0.34	Nodiff	4'-Chloro-2-hydroxy-4-methoxybenzophen

					one
M1522.neg	-1.18	0.83	0.18	Nodiff	Leonubiastrin
M1523.pos	0.15	0.55	0.54	Nodiff	2-Methoxynaphthalene
M1524.pos	-0.04	0.98	0.04	Nodiff	N-(3-Fluorophenyl)-2-(1-piperazinyl)acetamide
M1525.pos	0.05	0.99	0.09	Nodiff	Indole-3-acetamide
M1526.neg	-0.21	0.65	0.45	Nodiff	1-Methyl-3-(trifluoromethyl)-1H-pyrazole-4-carboxylic acid
M1528.neg	-0.51	0.48	0.81	Nodiff	Phloroglucinolcarboxylic acid
M1529.pos	-0.08	0.43	0.70	Nodiff	DG(14:0/20:5(5Z,8Z,11Z,14Z,17Z)/0:0)
M153.pos	0.10	0.74	0.38	Nodiff	4-(Methylamino)butanoic acid
M1530.pos	-0.57	0.57	0.53	Nodiff	Desisopropyldisopyramide
M1531.neg	0.06	0.67	0.47	Nodiff	Derenofylline
M1532.pos	-0.55	0.31	0.98	Nodiff	Magnolin
M1533.pos	-0.22	0.98	0.12	Nodiff	5-Bromo-4-hydrazino-6-methylpyrimidine
M1534.neg	-0.14	0.92	0.05	Nodiff	Aegineoside
M1535.neg	0.54	0.38	0.92	Nodiff	2,4-Bis(4-hydroxyphenyl)cyclobutane-1,3-dicarboxylic acid
M1536.pos	-0.01	0.45	0.73	Nodiff	.alpha.-Pyrrolidinononanophenone
M1537.pos	-1.79	0.04	1.84	Nodiff	rac-5-Carboxy-desisopropyltolterodine methyl ester
M1538.neg	0.93	0.14	1.40	Nodiff	6.beta.-Naltrexol
M1539.pos	1.37	0.08	1.67	Nodiff	7.alpha.-Thiospironolactone
M154.pos	0.13	0.65	0.49	Nodiff	Threonine
M1540.neg	-0.69	0.36	0.87	Nodiff	4-Bromophenol
M1541.neg	2.23	0.17	1.38	Nodiff	Arg-Met
M1542.pos	-0.54	0.37	1.00	Nodiff	Cyclobenzaprine
M1543.pos	-0.02	0.48	0.76	Nodiff	(3aS,5S,11bR,E)-12-Ethylidene-1,2,3a,4,5,7-hexahydro-3,5-ethanopyrrolo[2,3-d]carbazole-6-carbaldehyde
M1545.neg	0.15	0.69	0.41	Nodiff	2-(2-Thienyl)-1H-benzimidazole
M1546.pos	-0.56	0.84	0.21	Nodiff	15-Lipoxygenase Inhibitor I
M1547.pos	-0.30	0.62	0.57	Nodiff	(3b,4b,11b,14b)-11-Ethoxy-3,4-epoxy-14-hydroxy-12-cyathen-15-al_14-xyloside
M1548.pos	0.17	0.54	0.61	Nodiff	Lanthionine_ketimine
M1549.neg	-0.45	0.50	0.65	Nodiff	2-Hydroxy-3-(trifluoromethoxy)benzoic acid
M1550.pos	2.55	0.13	1.46	Nodiff	Loratadine
M1551.pos	-1.01	0.38	0.77	Nodiff	Thiomiltefosine
M1552.pos	0.66	0.35	0.91	Nodiff	Mollicellin I
M1553.neg	0.90	0.05	1.79	Nodiff	2'-Hydroxy-4-methoxychalcone
M1554.pos	-0.11	0.78	0.32	Nodiff	Benzenemethanol, p-fluoro-.alpha.-[1-(methylamino)ethyl]-, erythro-(.+/-.)-
M1555.pos	-0.50	0.96	0.06	Nodiff	N-(3-Chloro-4-methylphenyl)-4-nitrobenzamide
M1556.neg	-0.38	0.80	0.29	Nodiff	Phe(Benzoyl)-Leu-Arg
M1557.neg	0.66	0.26	1.18	Nodiff	Tetrafluorosuccinic acid
M1558.pos	0.42	0.30	0.98	Nodiff	Naphtho[2,3-b]furan-2(4H)-one, 4a,5,6,7,8,8a,9,9a-octahydro-3,8a-dimethyl-5-methylene-
M1559.pos	0.30	0.41	0.79	Nodiff	Pirenperone
M156.neg	0.71	0.29	1.16	Nodiff	N-Acetylcarnosine
M1560.neg	-0.31	0.35	0.86	Nodiff	6,6'-Dihydroxy-5,5'-dimethoxybiphenyl-3,3'-dicarboxylic acid
M1561.pos	-0.20	0.31	1.07	Nodiff	1,3,5-Triazine-2-methanol, 4-amino-6-(phenylamino)-, 2-(4-nitrobenzoate)
M1562.pos	-0.75	0.19	1.41	Nodiff	Amoxicillin
M1563.neg	-0.77	0.09	1.48	Nodiff	4-tert-Butyl-N'-[(E)-(2-hydroxynaphthalen-1-yl)methylidene]benzohydrazide
M1564.neg	0.13	0.54	0.59	Nodiff	3-[4-(4-Hydroxy-2-quinazolinyl)phenyl]-1,3-oxazolidin-2-one
M1565.pos	0.86	0.34	0.95	Nodiff	Ethyl

					5-(2-chloroacetyl)-2,4-dimethyl-1H-pyrrole-3-carboxylate
M1566.neg	-0.24	0.52	0.58	Nodiff	5-(2-Hydroxybenzylidene)-2-thioxodihydro-4,6(1H,5H)-pyrimidinedione
M1567.pos	0.10	0.42	0.79	Nodiff	2-tert-Pentylanthra-9,10-quinone
M1568.pos	-0.87	0.08	1.61	Nodiff	Aureonitol
M1569.pos	0.08	0.78	0.35	Nodiff	2-Chloro-N-(prop-2-en-1-yl)acetamide
M157.neg	0.84	0.34	0.98	Nodiff	Lysine, N2,N6-diacetyl-
M1570.pos	-0.55	0.49	0.76	Nodiff	Luotonin A
M1571.pos	-0.25	0.43	0.82	Nodiff	Xanthophyll
M1572.pos	-0.44	0.39	0.96	Nodiff	Trimethylaminoacetone
M1573.pos	0.53	0.20	1.30	Nodiff	3-[3-(2-Piperidinoethoxy)phenyl]-5-(1H-1,2,4-triazol-5-yl)-1H-indazole
M1574.neg	0.29	0.53	0.78	Nodiff	Benzamide, 3-[[(2E)-3-(1H-indol-3-yl)-1-oxo-2-propen-1-yl]amino]-
M1575.neg	-0.61	0.12	1.48	Nodiff	Gossypetin 3-methylether
M1576.pos	-0.43	0.39	0.97	Nodiff	1-(6-Chloropyridin-2-yl)piperazine
M1577.neg	0.64	0.35	0.93	Nodiff	3-(3-Hydroxy-5-methylphenoxy)-5-methylbenzene-1,2-diol
M1578.neg	0.22	0.42	0.83	Nodiff	1-{[3-(Trifluoromethyl)phenyl]sulfonyl}-4-piperidinecarboxylic acid
M1579.pos	-0.74	0.40	0.91	Nodiff	Artonol_B
M158.pos	0.39	0.38	0.89	Nodiff	Tryptophan
M1580.pos	1.55	0.08	1.48	Nodiff	N'-(Adamantane-1-carbonyl)isonicotinohydrazide
M1581.pos	0.63	0.57	0.65	Nodiff	Dihydroferuperine
M1582.neg	-0.39	0.61	0.48	Nodiff	1-Dehydroperuvinine
M1583.pos	0.46	0.26	1.12	Nodiff	5-(2-Chloroethyl)-5H-tetraazole
M1584.pos	0.28	0.42	0.81	Nodiff	4-Chloro-N-(3-hydroxyphenyl)benzamide
M1585.pos	0.52	0.34	0.94	Nodiff	6-Methoxy-3-nitro-2-pyridinamine
M1586.pos	-0.51	0.26	1.08	Nodiff	18-Hydroxycortisol
M1587.pos	0.29	0.41	0.87	Nodiff	Heptanedioic acid, 1-[2-[(2-carboxyphenyl)methylene]hydrazide]
M1588.neg	-0.86	0.51	0.67	Nodiff	Eriodictyol
M1589.neg	-0.09	0.84	0.31	Nodiff	Methyl (4aR,4bR,10aR,12aS)-6,10-dihydroxy-3,4b,7,7,10a,12a-hexamethyl-12-methylidene-1,4,5,8-tetraoxo-9,10,10b,11-tetrahydronaphtho[2,1-f]isochromene-4a-carboxylate
M159.neg	-0.37	0.49	0.67	Nodiff	Lactate
M1590.pos	-0.73	0.62	0.50	Nodiff	3,4-DIHYDROXYPHENYLGLYCOL
M1591.neg	0.63	0.48	0.73	Nodiff	Amastatin
M1592.neg	-1.69	0.29	1.09	Nodiff	7-Chloro-8-methyl-4-hydroxy-3-quinolinecarboxylic acid
M1593.pos	-0.58	0.17	1.31	Nodiff	Methyl_(9Z)-8'-oxo-6,8'-diapo-6-carotenoate
M1594.neg	0.13	0.45	0.79	Nodiff	9-Oxo-9H-fluorene-2-carboxylic acid
M1595.pos	0.94	0.04	1.93	Nodiff	N-(4-Butylphenyl)-4-methoxybenzamide
M1596.pos	-0.38	0.15	1.40	Nodiff	4-(4-Phenyl-1H-pyrazol-1-yl)piperidine
M1597.pos	-0.24	0.47	0.73	Nodiff	7-Diethylamino-3-phenylcoumarin
M1598.pos	0.51	0.38	0.88	Nodiff	Mukoline
M1599.pos	0.99	0.14	1.37	Nodiff	5-Chloro-2-(4-propionyl-1-piperazinyl)aniline
M16.neg	0.68	0.18	1.41	Nodiff	Citrulline
M160.neg	-0.08	0.84	0.15	Nodiff	Nicotinate
M1600.pos	0.36	0.31	1.07	Nodiff	5,10-Dihydro-10-phenophosphazinol 10-oxide
M1601.pos	0.32	0.40	0.88	Nodiff	hexadecanedioic_acid_mono-L-carnitine_ester
M1602.pos	-0.14	0.94	0.11	Nodiff	N1,N10-Diferuloylspermidine
M1603.neg	-0.07	0.86	0.16	Nodiff	Maclurin
M1604.neg	0.98	0.30	1.07	Nodiff	N-[1-(1H-Benzimidazol-2-yl)-3-methyl-1H-

					pyrazol-5-yl]acetamide
M1605.pos	-0.63	0.06	1.66	Nodiff	N-(2-Fluoro-5-methylphenyl)benzamide
M1606.pos	0.13	0.76	0.24	Nodiff	4-(2-Thienyl)-2-pyrimidinylamine
M1607.pos	0.27	0.67	0.34	Nodiff	DG(20:4(8Z,11Z,14Z,17Z)/14:0/0:0)
M1608.pos	-0.52	0.37	0.83	Nodiff	3-[(4-Fluoroanilino)carbonyl]-1,2,2-trimethylcyclopentanecarboxylic acid
M1609.neg	1.25	0.03	1.85	Nodiff	Hexamethylquercetagetin
M161.pos	0.31	0.41	0.89	Nodiff	Pipecolamide
M1610.pos	-0.33	0.22	1.15	Nodiff	Tolfenpyrad
M1611.pos	1.03	0.32	0.97	Nodiff	Pro-Gly-Pro
M1612.pos	1.66	0.03	1.87	Nodiff	1H-Pyrrole-2,5-dione, 1-methyl-3-(1-methyl-1H-indol-3-yl)-4-(pentylamino)-
M1613.pos	0.70	0.35	0.99	Nodiff	Pipernonaline
M1614.neg	-0.09	0.96	0.02	Nodiff	3-Oxo-2-[3-(trifluoromethyl)phenyl]-2,3-dihydro-1H-isoindole-4-carboxylic acid
M1615.neg	-0.41	0.17	1.29	Nodiff	2-Methyl-3-[(tetrahydro-2-furanylcarbonyl)amino]benzoic acid
M1616.neg	-0.08	0.56	0.62	Nodiff	Saluamine
M1617.pos	0.88	0.33	0.95	Nodiff	N2-Methyl-L-arginine
M1618.pos	-1.63	0.14	1.44	Nodiff	METHYLPREDNISOLONE
M1619.pos	0.34	0.46	0.71	Nodiff	Lysyl-Glycine
M162.pos	-0.26	0.91	0.12	Nodiff	Phe-Ala
M1620.pos	-0.85	0.45	0.78	Nodiff	5-tert-Butyl-2-hydroxybenzophenone
M1621.pos	-0.13	0.98	0.04	Nodiff	Androsta-1,4-dien-3-one, 4-chloro-6,17-dihydroxy-17-methyl-, (6.beta.,17.beta.)-
M1622.pos	0.90	0.18	1.36	Nodiff	Triptonide
M1623.pos	-0.31	0.34	0.89	Nodiff	5-(2-Oxohexahydro-1H-thieno[3,4-d]imidazol-4-yl)pentanohydrazide
M1624.pos	1.21	0.20	1.28	Nodiff	Oxynarcotine
M1625.neg	1.22	0.18	1.25	Nodiff	Rhapontigenin
M1626.neg	0.86	0.17	1.40	Nodiff	4-(Prop-2-en-1-yl)phenyl 6-O-hexopyranosylhexopyranoside
M1627.neg	0.01	0.82	0.31	Nodiff	3,4-Difluoro-L-phenylalanine
M1628.pos	-0.21	0.49	0.67	Nodiff	N-Benzyl-N'-(3-methoxyphenyl)thiourea
M1629.pos	-0.41	0.54	0.67	Nodiff	Methanone, (4-hydroxyphenyl)(1-pentyl-1H-indol-3-yl)-
M163.neg	0.26	0.36	0.89	Nodiff	N-Acetylmethionine
M1630.neg	0.21	0.51	0.68	Nodiff	2-Hydroxy-3-(1H-indol-3-yl)butanoic acid
M1631.pos	1.23	0.12	1.55	Nodiff	Pipertipine
M1632.pos	0.13	0.68	0.37	Nodiff	N1,N10-Dicoumaroylspermidine
M1633.pos	0.08	0.64	0.42	Nodiff	L-alpha-Amino-1H-pyrrole-1-hexanoic_acid
M1634.pos	1.91	0.79	0.19	Nodiff	N-(4-Acetylphenyl)benzenesulfonamide
M1635.neg	0.63	0.19	1.37	Nodiff	3.alpha.,4.beta.,3.alpha.-Galactotetraose
M1636.pos	0.56	0.34	1.02	Nodiff	Butalbital
M1637.pos	-0.08	0.78	0.31	Nodiff	1-[(4,8-Dimethoxyfuro[2,3-b]quinolin-7-yl)oxy]-3-methylbutane-2,3-diol
M1638.pos	-0.51	0.49	0.70	Nodiff	Cocaine
M1639.pos	-0.25	0.30	1.06	Nodiff	5-(4-Methylpiperazin-1-yl)-5-oxopentanoic acid
M164.neg	0.69	0.71	0.48	Nodiff	Taurocholic acid
M1640.neg	0.45	0.90	0.08	Nodiff	3-(4-Bromophenyl)-.beta.-alanine
M1641.neg	0.33	0.38	0.91	Nodiff	N-(6-Amino-1-butyl-2,4-dioxo-1,2,3,4-tetrahydro-5-pyrimidinyl)-N-methylbenzamide
M1642.neg	-1.08	0.17	1.33	Nodiff	2',6'-dihydroxy-4'-methoxydihydrochalcone
M1643.pos	-0.16	0.71	0.44	Nodiff	Prunetin
M1644.pos	0.82	0.17	1.38	Nodiff	(E)-Piperolein_A
M1645.pos	0.14	0.52	0.70	Nodiff	1-Butanone, 1-(1,3-benzodioxol-5-yl)-2-(dimethylamino)-

M1646.pos	0.04	0.89	0.08	Nodiff	N-[(Ethoxycarbonyl)methyl)-p-menthane-3-carboxamide
M1647.pos	0.83	0.59	0.64	Nodiff	2,3-epoxyphylloquinone
M1648.pos	0.08	0.79	0.32	Nodiff	1-(2-Thienyl)-1-heptanone
M1649.pos	0.98	0.43	0.67	Nodiff	(5,6-Diphenylfuro[2,3-d]pyrimidin-4-yl)glycine
M165.pos	0.92	0.31	0.99	Nodiff	5-methyl-2'-deoxycytidine
M1650.pos	0.16	0.44	0.75	Nodiff	Gamma-glutamyl-Glutamine
M1651.pos	-0.97	0.09	1.57	Nodiff	Veratraldehyde, azine
M1653.pos	-0.65	0.04	1.74	Nodiff	Heptanedioic acid, 1-(2-cyclopentylidenehydrazide)
M1654.pos	0.16	0.44	0.77	Nodiff	Preglabridin
M1655.neg	1.37	0.00	2.25	Nodiff	Etodolac
M1656.pos	0.10	0.68	0.45	Nodiff	2-{[2-(3,4-Dihydroxyphenyl)-2-oxoethyl]sulfanyl}-6-methyl-4(3H)-pyrimidinone
M1657.pos	-0.85	0.19	1.42	Nodiff	Dehydrogriseofulvin
M1658.pos	0.08	0.44	0.74	Nodiff	Dehydrocostus lactone
M1659.pos	0.68	0.19	1.29	Nodiff	.beta.-Alanylleucine
M166.pos	-0.23	0.95	0.02	Nodiff	3-AMINO-2-PIPERIDONE
M1660.neg	0.15	0.42	0.90	Nodiff	3-(2H-Tetraazol-5-yl)benzoic acid
M1661.pos	-1.56	0.01	2.02	Nodiff	Isovalerylglutamic_acid
M1662.neg	-0.28	0.27	1.17	Nodiff	Sophoraside A
M1663.pos	0.51	0.26	1.08	Nodiff	THA
M1664.pos	0.78	0.35	0.98	Nodiff	2-(Acetylamino)-N-[2-(1-piperidinyl)phenyl]-1,3-thiazole-4-carboxamide
M1665.pos	0.25	0.38	0.90	Nodiff	8R-HpODE
M1666.pos	8.00	0.19	1.43	Nodiff	Ethyl 6-chloronicotinate
M1667.pos	0.59	0.27	1.06	Nodiff	7-Benzyloxy-2-(2,2-diphenyl-1,3-benzodioxol-5-yl)-5-hydroxy-H-1-benzopyran-4-one
M1668.pos	1.26	0.07	1.64	Nodiff	Isopropylcocaine
M1669.pos	0.09	0.93	0.13	Nodiff	5-[(4-Chlorobenzyl)sulfanyl]-1,3,4-thiadiazol-2-ylamine
M167.pos	-0.30	0.34	0.98	Nodiff	FAPy-adenine
M1670.neg	0.16	0.50	0.75	Nodiff	3-(Trifluoromethyl)cinnamic acid
M1671.pos	0.19	0.42	0.77	Nodiff	N-(4-Hydroxyphenyl)arachidonoyl amide
M1672.pos	3.55	0.07	1.66	Nodiff	PC(P-18:1(9Z)/0:0)
M1673.pos	0.23	0.46	0.70	Nodiff	DG(18:4(6Z,9Z,12Z,15Z)/15:0/0:0)
M1674.pos	-0.20	0.46	0.67	Nodiff	(4-Methyl-1-piperazinyl)acetic acid
M1675.pos	-0.86	0.33	1.08	Nodiff	N-(1,3-Dioxo-2,3-dihydro-1H-isoindol-5-yl)propanamide
M1676.neg	-0.69	0.22	1.25	Nodiff	(S)-3,5-Dihydroxyphenylglycine
M1677.neg	-0.92	0.09	1.62	Nodiff	5-(4-Chlorobenzylidene)-2,4,6(1H,3H,5H)-pyrimidinetrione
M1678.pos	-0.71	0.96	0.02	Nodiff	TG(18:2(9Z,12Z)/14:0/18:3(9Z,12Z,15Z))[iso6]
M1679.neg	-0.33	0.22	1.14	Nodiff	L-Glutamic acid .gamma.-(.beta.-naphthylamide)
M168.neg	0.05	0.49	0.73	Nodiff	3-Hydroxycinnamic acid
M1680.pos	1.31	0.10	1.55	Nodiff	N-[2-(5,6-Dimethyl-1H-benzimidazol-2-yl)ethyl]-4-(4H-1,2,4-triazol-4-yl)benzamide
M1681.pos	0.23	0.40	0.81	Nodiff	N-Acetylsulfamethoxazole
M1682.pos	0.51	0.38	0.90	Nodiff	N-Acetylretigabine
M1683.neg	-0.08	0.78	0.31	Nodiff	4-Acetyloxy-6-hydroxy-2-(2-hydroxypropan-2-yl)-4a,6-dimethyl-3,4,5,7,8,8a-hexahydro-2H-chromene-5-carboxylic acid
M1684.pos	0.43	0.23	1.23	Nodiff	8-Imino-2',5',5',8a'-tetramethyl-3,3',4',4a',5',6,6',7,7',8,8',8a'-dodecahydro-2'H-spiro[furo[2,3-e]isoindole-2,1'-naphthalene]-4,6'-diol
M1685.pos	0.97	0.13	1.49	Nodiff	Formyldienolone
M1686.pos	0.42	0.10	1.46	Nodiff	Chitin
M1687.pos	0.66	0.08	1.60	Nodiff	putative tryptophane conjugated chenodeoxycholic acid (clustered spectrum)
M1688.neg	0.34	0.21	1.26	Nodiff	2,2'-Dihydroxy-4-methoxybenzophenone

M1689.neg	-0.85	0.56	0.61	Nodiff	HMBOA + O-Hex
M169.pos	0.17	0.42	0.86	Nodiff	Creatinine
M1690.pos	-1.35	0.30	1.14	Nodiff	Distichonic_acid_A
M1691.pos	0.09	0.72	0.42	Nodiff	L-Homoserine lactone
M1692.pos	-0.12	0.67	0.50	Nodiff	.beta.-Amyrenonol
M1693.pos	0.73	0.33	0.97	Nodiff	Trospium
M1694.pos	0.10	0.59	0.50	Nodiff	Malonyldaidzin
M1695.pos	-0.39	0.13	1.46	Nodiff	N-Benzyl-L-isoleucine methyl ester
M1696.pos	-0.11	0.73	0.37	Nodiff	5-Megastigmen-7-yne-3,9-diol_3-glucoside
M1697.neg	-1.18	0.17	1.51	Nodiff	Sinensetin
M1698.pos	1.23	0.10	1.59	Nodiff	Pesticide4_Tebufenozide_C22H28N2O2_Benzoic acid, 3,5-dimethyl-, 1-(1,1-dimethylethyl)-2-(4-ethylbenzoyl)hydrazide
M1699.neg	-0.43	0.22	1.12	Nodiff	2-[(2-Fluorophenyl)amino]acetohydrazide
M17.neg	0.04	0.67	0.49	Nodiff	Serine
M170.neg	-0.30	0.53	0.65	Nodiff	1-Methylpseudouridine
M1700.pos	0.31	0.40	0.88	Nodiff	22-Hydroxymifepristone
M1701.pos	-0.05	0.79	0.21	Nodiff	6''-Malonylcosmosiin
M1702.neg	-0.11	0.94	0.04	Nodiff	Loganic acid
M1703.neg	-1.44	0.05	1.74	Nodiff	Platanionoside C
M1704.pos	-0.39	0.29	1.04	Nodiff	Trp-Ser
M1705.neg	-0.18	0.98	0.01	Nodiff	Niflumic acid
M1706.pos	-1.09	0.11	1.58	Nodiff	AS_1-5
M1707.pos	1.16	0.26	1.03	Nodiff	R-Palmitoyl-(2-methyl) ethanolamide
M1708.neg	-0.63	0.53	0.71	Nodiff	5-Aminoorotic acid
M1709.pos	1.50	0.80	0.32	Nodiff	3-Methyl-5-pentyl-2-furannonanoic_acid
M171.neg	3.14	0.10	1.65	Nodiff	2'-O-Methylinosine
M1710.pos	-0.26	0.69	0.46	Nodiff	(Z,Z)-2,9,16-Heptadecatriene-4,6-diyn-8-ol
M1711.pos	0.23	0.23	1.20	Nodiff	.beta.-Glutamic acid
M1712.neg	1.77	0.05	1.75	Nodiff	Nuezhenide
M1713.pos	0.42	0.27	1.17	Nodiff	16.beta.-Hydroxyfurazabol
M1714.neg	2.92	0.08	1.71	Nodiff	2-Amino-4,5-difluorobenzoic acid
M1715.pos	-0.74	0.12	1.39	Nodiff	6-(1-Naphthyl)-5-nitro-2-piperidinone
M1716.pos	-0.14	0.84	0.16	Nodiff	6'-Malonyltrifolirhizin
M1717.pos	-0.94	0.73	0.37	Nodiff	Ganoderic_acid_Md
M1718.pos	1.92	0.45	0.67	Nodiff	1-(2-Fluorophenyl)-5-oxo-3-pyrrolidinecarboxylic acid
M1719.neg	0.72	0.20	1.29	Nodiff	Benzoic acid, 2-[3-(3,7-dimethyl-2,6-octadien-1-yl)-2,6-dihydroxybenzoyl]-5-formyl-3-hydroxy-
M172.neg	-0.65	0.31	1.01	Nodiff	Indolelactic acid
M1720.pos	1.59	0.42	0.71	Nodiff	Methyl arachidonyl fluorophosphonate
M1721.pos	-0.06	0.45	0.81	Nodiff	Trihexyphenidyl
M1722.neg	0.08	0.67	0.47	Nodiff	Benz[a]anthracen-1-ol, 8-methoxy-3-methyl-
M1723.pos	1.13	0.32	1.06	Nodiff	Lasiodiplodin
M1724.pos	-0.18	0.62	0.55	Nodiff	Lewis_a_trisaccharide
M1725.pos	0.36	0.66	0.40	Nodiff	(1-Methyl-1H-pyrazol-5-yl)methanol
M1726.pos	0.22	0.47	0.65	Nodiff	Ifenprodil
M1727.pos	2.17	0.20	1.25	Nodiff	Lactodifucotetraose
M1728.pos	0.52	0.38	0.93	Nodiff	Leptomycin B
M1729.pos	2.10	0.10	1.64	Nodiff	2-Carboxy-1-[5-(2-carboxy-1-pyrrolidinyl)-2-hydroxy-2,4-pentadienylidene]pyrrolidinium
M173.neg	-0.57	0.36	0.98	Nodiff	Taurolithocholic acid
M1730.pos	-0.18	0.58	0.66	Nodiff	Soyasaponin_IV
M1731.neg	-0.51	0.31	1.06	Nodiff	5-Methoxypsoralen
M1732.pos	1.15	0.06	1.67	Nodiff	SM(d18:0/16:0)
M1733.pos	-0.40	0.45	0.80	Nodiff	N.alpha.-Benzoyl-DL-arginine-4-nitroanilide
M1734.pos	-0.42	0.89	0.11	Nodiff	(3aS,8aR)-1,3a,8-trimethyl-1,2,3,3a,8,8a-hexahydropyrrolo[2,3-b]indol-5-yl methylcarbamate

M1735.pos	-0.89	0.42	0.85	Nodiff	24-epi-brassinolide
M1736.neg	0.07	0.45	0.72	Nodiff	1-(10Z-Heptadecenoyl)-sn-glycero-3-phospho-(1'-rac-glycerol)
M1737.neg	0.13	0.62	0.54	Nodiff	gardenoside
M1738.neg	0.93	0.34	0.99	Nodiff	Dicaffeoyl quinic acid
M1739.pos	-0.80	0.05	1.77	Nodiff	N-(3-Methylphenyl)-4-quinazolinamine
M174.neg	1.11	0.31	1.04	Nodiff	13(S)-HODE
M1740.pos	-0.36	0.70	0.41	Nodiff	Hexaconazole
M1741.pos	0.01	0.88	0.18	Nodiff	Lysyl-Aspartate
M1742.neg	-0.12	0.74	0.33	Nodiff	13-(Dodecan-2-yl)-6-(1-hydroxyethyl)-3-(hydroxymethyl)-12-methyl-9-(propan-2-yl)-1-oxa-4,7,10-triazacyclotridecane-2,5,8,11-tetrone
M1743.neg	-0.50	0.17	1.25	Nodiff	2-Phenylethyl 6-O-pentopyranosylhexopyranoside
M1744.pos	0.09	0.65	0.43	Nodiff	2-Hydroxy-5-[(3,4,5-trimethoxybenzoyl)amino]benzoic acid
M1745.neg	-0.34	0.41	0.77	Nodiff	2,4,5,6(1H,3H)-Pyrimidinetetrone, 5-[2-(2-chlorophenyl)hydrazone]
M1746.pos	-0.22	0.83	0.19	Nodiff	Ethyl 4-amino-1-piperidinecarboxylate
M1747.pos	0.39	0.34	0.93	Nodiff	Agaritinal
M1748.pos	0.01	0.46	0.80	Nodiff	5-Methoxy-8,8-dimethyl-2-phenyl-2,3,8,8a-tetrahydro-4H,7aH-cyclopropa[4,5]furo[2,3-h]chromen-4-one
M1749.neg	1.50	0.13	1.50	Nodiff	Lisinopril (8R,S)-diketopiperazine
M175.neg	0.21	0.50	0.62	Nodiff	beta-Muricholic acid
M1750.pos	0.00	0.58	0.53	Nodiff	Desmethylofloxacin
M1751.pos	-0.22	0.44	0.86	Nodiff	4,5-Dihydro-2-methylthiazole
M1752.pos	0.58	0.57	0.58	Nodiff	Ganoderic_acid_H
M1753.pos	-0.59	0.52	0.62	Nodiff	Ethyl 6-methyl-4-(4-methylphenyl)-2-oxo-1,2,3,4-tetrahydro-5-pyrimidinecarboxylate
M1754.neg	-2.21	0.46	0.71	Nodiff	Harman
M1755.pos	0.27	0.55	0.54	Nodiff	(4R)-4-((2S,3S,5R,7R,9S,10S,13R,14S,17R)-2,3,7-trihydroxy-10,13-dimethylhexadecahydro-1H-cyclopenta[a]phenanthren-17-yl)pentanoic acid
M1756.pos	-0.62	0.36	0.87	Nodiff	Imazethapyr
M1757.pos	0.84	0.33	0.99	Nodiff	1-(4-Methoxybenzyl)-3-acetoxynorcotinine
M1758.neg	-0.69	0.06	1.62	Nodiff	2,3-Quinoxalinedione, 6-chloro-1,4-dihydro-
M1759.pos	0.57	0.32	0.89	Nodiff	8,11-Tridecadienoic acid, 13-(3-pentyl-2-oxiranyl)-, methyl ester, (8Z,11Z)-
M176.neg	0.11	0.66	0.52	Nodiff	beta-Alanine
M1760.pos	-0.13	0.49	0.76	Nodiff	N-Acetylleucylalanylserine
M1761.pos	-1.50	0.71	0.33	Nodiff	Panaxydiol
M1762.pos	0.25	0.76	0.32	Nodiff	N-Butyl-2,4-dinitroaniline
M1763.pos	-0.37	0.09	1.53	Nodiff	Ala-Val
M1764.pos	-0.12	0.65	0.56	Nodiff	Soyasaponin_II
M1765.neg	-0.16	0.88	0.07	Nodiff	Thymidine_3,5-cyclic_monophosphate
M1766.pos	0.07	0.69	0.41	Nodiff	Candoxatril
M1767.pos	-0.07	0.75	0.38	Nodiff	Deferoxamine
M1768.neg	-0.39	0.92	0.07	Nodiff	3-Nitro-1H-pyrazole-5-carboxylic acid
M1769.pos	-0.43	0.45	0.64	Nodiff	2-Oleoyl-1-stearoyl-sn-glycero-3-phosphoserine
M177.neg	0.99	0.67	0.51	Nodiff	Taurohyodeoxycholic acid
M1770.pos	0.15	0.49	0.64	Nodiff	Ricinoleic acid methyl ester
M1771.neg	-0.28	0.95	0.04	Nodiff	2-Chloro-N-(2-hydroxyphenyl)nicotinamide
M1772.pos	-0.40	0.94	0.09	Nodiff	3-cis-Hydroxy-b,e-Caroten-3'-one
M1773.pos	-0.97	0.59	0.50	Nodiff	N-(1-Phenylethyl)-N-(2-phenyl-4-quinazolinyl)amine
M1774.pos	0.90	0.20	1.27	Nodiff	6a_Methylprednisolone

M1775.pos	1.32	0.00	2.19	Nodiff	Feruperine
M1776.neg	1.22	0.07	1.70	Nodiff	3-O-Feruloylquinic acid
M1777.neg	0.87	0.33	1.07	Nodiff	2-Hydroxy-2-(2-methoxy-2-oxoethyl)butanedioic acid
M1778.pos	-1.10	0.26	1.23	Nodiff	N-(2,5-Dichlorophenyl)-2-hydroxybenzamide
M1779.pos	0.37	0.52	0.57	Nodiff	(4R)-4-((1R,3S,5S,7R,9S,10S,13R,14S,17R)-1,3,7-trihydroxy-10,13-dimethylhexadecahydro-1H-cyclopenta[a]phenanthren-17-yl)pentanoic acid
M178.neg	1.09	0.09	1.70	Nodiff	Methyl beta-D-glucopyranoside
M1780.pos	0.46	0.30	1.02	Nodiff	LysoPC(20:4(5Z,8Z,11Z,14Z))
M1781.pos	-0.87	0.40	0.91	Nodiff	Demethylcalabaxanthone
M1782.pos	0.49	0.27	1.04	Nodiff	Ginkgolic acid II
M1783.neg	0.80	0.31	1.10	Nodiff	4-[(Phenylsulfanyl)methyl]benzoic acid
M1784.neg	-0.69	0.25	1.16	Nodiff	2-((3-Bromophenyl)amino)benzoic acid
M1785.pos	1.42	0.11	1.51	Nodiff	Paxilline
M1786.pos	-0.05	0.46	0.73	Nodiff	Epiafzelechin (2R,3R)(-)
M1787.pos	0.40	0.33	0.90	Nodiff	Pirinixic acid aminothiazole
M1788.pos	0.86	0.23	1.20	Nodiff	2-(2H-Benzotriazol-2-yl)-4,6-bis(1-methyl-1-phenylethyl)phenol
M1789.pos	-0.03	0.47	0.71	Nodiff	O-t-Butyl-L-threonine methyl ester
M179.neg	0.20	0.49	0.71	Nodiff	p-Toluquinone
M1790.neg	-0.75	0.16	1.30	Nodiff	8-Cinnamoyl-5,7-dihydroxy-2,2,6-trimethylchromene
M1791.neg	0.78	0.00	2.22	Nodiff	(S)-Nitroblebbistatin
M1792.pos	0.39	0.40	0.90	Nodiff	2-(2-Methoxyethoxy)ethanamine
M1793.neg	0.01	0.46	0.77	Nodiff	3-(.alpha.-L-Arabinofuranosyloxy)pimara-8(14),15-dien-12-yl 6-deoxy-.beta.-D-galactopyranoside
M1794.pos	-0.45	0.94	0.10	Nodiff	TXB2
M1795.pos	0.80	0.18	1.34	Nodiff	LysoPC(18:3(6Z,9Z,12Z))
M1796.pos	-0.32	0.26	1.12	Nodiff	3-(1H-Indol-4-yl)-N-(3-methoxypropyl)-1,2,4-oxadiazole-5-carboxamide
M1797.neg	1.63	0.07	1.76	Nodiff	4',5,7-Trihydroxy 3,3',6,8-tetramethoxyflavone
M1798.pos	-0.73	0.45	0.82	Nodiff	PC(20:3(5Z,8Z,11Z)/14:0)
M1799.pos	0.52	0.38	0.93	Nodiff	(E)-3-(3-(2,4-Dimethoxybenzylidene)-3,4,5,6-tetrahydropyridin-2-yl)pyridine
M18.neg	0.65	0.38	0.92	Nodiff	Methionine sulfoxide
M180.neg	0.84	0.12	1.47	Nodiff	Galactose
M1800.pos	0.23	0.43	0.81	Nodiff	3-(4-Methyl-3-pentenyl)thiophene
M1801.pos	0.45	0.36	0.85	Nodiff	Ganoderal_A
M1802.pos	-2.33	0.74	0.32	Nodiff	Biphenyl-4-yl-p-tolylmethanone
M1803.pos	-0.42	0.22	1.19	Nodiff	Thyrotropin-Releasing Hormone
M1804.neg	0.02	0.47	0.74	Nodiff	2-Cyano-N-[4-(1,3-thiazol-2-ylsulfamoyl)phenyl]acetamide
M1805.pos	0.86	0.12	1.60	Nodiff	1,8-Diethyl-1,3,4,9-tetrahydro-4-oxo-pyrano[3,4-b]indole-1-acetic acid methyl ester
M1806.pos	-0.06	0.33	0.95	Nodiff	Asticolorin_B
M1807.pos	-0.63	0.36	0.83	Nodiff	N-[2-(1-Piperidinyl)-5-(trifluoromethyl)phenyl]-4-pyridinecarboxamide
M1808.neg	0.61	0.18	1.36	Nodiff	.beta.-Hydroxyphenylalanine
M1809.pos	1.60	0.15	1.41	Nodiff	LysoPC(22:4(7Z,10Z,13Z,16Z))
M181.neg	0.50	0.42	0.77	Nodiff	Ursocholic acid
M1810.neg	-0.39	0.56	0.60	Nodiff	1-Naphthoic acid
M1811.neg	-0.36	0.56	0.67	Nodiff	Benzamide, 2-chloro-5-nitro-N-phenyl-
M1812.pos	-0.23	0.96	0.14	Nodiff	(22E,24R)-Stigmasta-4,22-diene-3,6-dione
M1813.pos	-0.10	0.50	0.69	Nodiff	3,3',5,5'-Tetraisopropylbiphenyl-4,4'-diol
M1814.pos	0.32	0.41	0.79	Nodiff	Ethyl 4-(4-methylpiperazin-1-yl)butanoate
M1815.pos	1.42	0.13	1.30	Nodiff	Cavipetin_C
M1816.neg	-1.18	0.17	1.37	Nodiff	(1R,4S,5S,6S)-4-Amino-2-thiabicyclo[3.1.0]hexane-4,6-dicarboxylic acid 2,2-dioxide
M1817.pos	0.15	0.53	0.56	Nodiff	Ofloxacin N-oxide

M1818.pos	1.14	0.76	0.37	Nodiff	Hecogenin
M1819.pos	0.24	0.68	0.37	Nodiff	(3beta,5alpha,6beta,22E,24R)-23-Methylergosta-7,22-diene-3,5,6-triol
M182.neg	3.14	0.10	1.65	Nodiff	3'-O-Methylinosine
M1820.pos	-0.32	0.39	0.96	Nodiff	1H-Pyrazole-1-carboximidamide
M1821.pos	-0.59	0.14	1.31	Nodiff	N-cis-Tetradec-9-enoyl-L-homoserine lactone
M1822.pos	1.05	0.53	0.73	Nodiff	4-(1,3-Thiazol-2-yl)piperazine-1-carbothioamide
M1823.pos	-0.16	0.50	0.66	Nodiff	Egenine
M1824.pos	0.13	0.46	0.69	Nodiff	Aspochalasin D
M1825.neg	-0.45	0.26	1.09	Nodiff	2-Methoxy-N-(1H-tetraazol-5-yl)benzamide
M1826.pos	0.25	0.25	1.15	Nodiff	Neoquassin
M1827.pos	0.27	0.41	0.79	Nodiff	4-Cholesten-3-one
M1828.neg	0.46	0.33	0.98	Nodiff	3-(Carboxymethyl)-1-.beta.-D-glucopyranosyl-1H-indole
M1829.neg	0.17	0.47	0.69	Nodiff	SUDCA - Sulfoursodeoxycholic acid
M183.neg	0.00	0.73	0.39	Nodiff	Arginine
M1830.neg	-2.81	0.14	1.59	Nodiff	4-Methanesulfonyl-2-nitrobenzoic acid
M1831.pos	0.99	0.12	1.34	Nodiff	Acetildenafil
M1832.neg	-0.79	0.10	1.49	Nodiff	(p-Tolylsulfonyl)acetic acid
M1833.pos	0.57	0.17	1.36	Nodiff	LysoPC(18:1(11Z))
M1834.pos	-0.58	0.24	1.23	Nodiff	1H-Indole-3-carboxamide, 1-(5-fluoropentyl)-N-2-naphthalenyl-
M1835.pos	-0.33	0.27	1.16	Nodiff	1-(sn-Glycero-3-phospho)-1D-myo-inositol
M1836.pos	1.05	0.11	1.53	Nodiff	L-Alaninamide, N-[2-[2-(hydroxyamino)-2-oxoethyl]-4-methyl-1-oxopentyl]-3-methyl-L-valyl-N-(2-aminoethyl)-
M1837.neg	0.36	0.34	1.00	Nodiff	1H-Pyrazole-4-carboxylic acid, 3-(aminosulfonyl)-, ethyl ester
M1838.pos	0.65	0.55	0.51	Nodiff	2(1H)-Pyrimidinone, 5-[3-[(1S,2S,4R)-bicyclo[2.2.1]hept-2-yloxy]-4-methoxyphenyl]tetrahydro-
M1839.pos	0.29	0.42	0.80	Nodiff	N-(2,5-Dioxo-1-pyrrolidinyl)-4-nitrobenzamide
M184.pos	0.94	0.03	1.84	Nodiff	Maltotriose
M1840.pos	0.47	0.39	0.84	Nodiff	N'-Hydroxyneosaxitoxin
M1841.neg	1.95	0.03	1.90	Nodiff	3',4',5,7-Tetrahydroxy-3,6,8-trimethoxyflavone
M1842.pos	-2.61	0.27	0.99	Nodiff	(R)-4-((1R,3S,5S,7R,8S,9S,10S,12S,13R,14S,17R)-1,3,7,12-tetrahydroxy-10,13-dimethylhexadecahydro-1H-cyclopenta[a]phenanthren-17-yl)pentanoic acid
M1843.pos	1.15	0.17	1.43	Nodiff	(2S)-5,7-Dihydroxy-6-prenylflavanone
M1844.pos	-0.86	0.37	0.79	Nodiff	Canthaxanthin
M1845.pos	1.38	0.25	1.10	Nodiff	(6beta,22E)-6-Hydroxystigmasta-4,22-dien-3-one
M1846.pos	-0.05	0.92	0.15	Nodiff	4-Iodosuberoylanilide hydroxamic acid
M1847.pos	1.05	0.29	1.12	Nodiff	Camylofine
M1848.pos	0.08	0.75	0.23	Nodiff	2,2-Diphenyl-N-(3-phenylpropyl)acetamide
M1849.neg	0.26	0.37	0.94	Nodiff	N-Acetyl-2-carboxybenzenesulfonamide
M185.pos	0.58	0.20	1.27	Nodiff	Isomaltose
M1850.pos	0.35	0.40	0.84	Nodiff	Alloxanthin
M1851.pos	0.06	0.72	0.42	Nodiff	5-(2-Aminoethyl)-1H-imidazol-2-amine
M1852.neg	0.21	0.52	0.67	Nodiff	1H-Indole-5-sulfonamide, N-(3-chlorophenyl)-3-[[3,5-dimethyl-4-[(4-methyl-1-piperazinyl)carbonyl]-1H-pyrrol-2-yl]methylene]-2,3-dihydro-N-methyl-2-oxo-, (3Z)-
M1853.neg	-0.37	0.40	0.77	Nodiff	Erythronolactone
M1854.pos	-0.44	0.68	0.44	Nodiff	N1-Acetylspermine

M1855.pos	0.04	0.76	0.33	Nodiff	Lamotrigine
M1856.pos	0.04	0.77	0.28	Nodiff	Daidzein
M1857.pos	-0.05	0.96	0.08	Nodiff	2-Acetylthiazole
M1858.pos	1.19	0.11	1.46	Nodiff	Glycyl-Tryptophan
M1859.neg	0.25	0.34	0.98	Nodiff	Flavanone base + 5O
M186.pos	-1.07	0.12	1.42	Nodiff	Glutarylcarnitine
M1860.pos	-0.60	0.11	1.50	Nodiff	Piracetam
M1861.pos	0.35	0.95	0.04	Nodiff	9,10,13-Trihydroxystearic_acid
M1862.neg	-0.35	0.97	0.08	Nodiff	CRUSTECDYSONE
M1863.neg	0.30	0.39	0.87	Nodiff	3-(2-(.beta.-D-Glucopyranosyloxy)-4-hydroxy-5-methoxyphenyl)-2-((2-oxo-2H-chromen-7-yl)oxy)propanoic acid
M1864.pos	-0.67	0.92	0.06	Nodiff	Psychosine
M1865.pos	1.03	0.74	0.45	Nodiff	Flavidulol_C
M1866.pos	-0.06	0.83	0.20	Nodiff	Mangiferdesmethylursanone
M1867.pos	0.01	0.44	0.82	Nodiff	16-Dehydropregnenolone acetate
M1868.pos	-0.46	0.55	0.56	Nodiff	Cafestol
M1869.pos	0.37	0.55	0.60	Nodiff	Olmesartan
M187.neg	-1.67	0.16	1.37	Nodiff	N-Acetylaspartic acid
M1870.neg	0.43	0.31	1.10	Nodiff	Astin C
M1871.neg	0.89	0.07	1.67	Nodiff	Coumarin-suberoylanilide hydroxamic acid
M1872.pos	-0.34	0.64	0.53	Nodiff	Monodesethylquinacrine
M1873.pos	-0.08	0.77	0.32	Nodiff	Succinamide
M1874.pos	2.21	0.13	1.51	Nodiff	Acrinathrin
M1875.pos	-0.98	0.49	0.67	Nodiff	1-Ethyl-N-(4-fluorophenyl)-2-oxo-1,2-dihydrobenzo[cd]indole-6-sulfonamide
M1876.pos	0.33	0.32	1.05	Nodiff	Encenicline
M1877.neg	0.28	0.37	0.80	Nodiff	Zinterol
M1878.neg	0.00	0.74	0.34	Nodiff	N-(4-Chlorobenzyl)-9H-purin-6-amine
M1879.neg	-0.37	0.45	0.75	Nodiff	Swertiamarin
M188.pos	0.57	0.32	0.98	Nodiff	Glycerophosphocholine
M1880.neg	-0.09	0.43	0.85	Nodiff	Gaylussacin
M1881.pos	1.04	0.87	0.16	Nodiff	Leukotriene E4
M1882.neg	-0.27	0.31	0.96	Nodiff	Antibiotic FR 901512
M1883.neg	-0.05	0.45	0.75	Nodiff	Ibotenic acid
M1884.pos	-0.01	0.79	0.22	Nodiff	Wistin
M1885.pos	0.23	0.51	0.61	Nodiff	Oleana-1,9(11)-dien-28-oic acid, 2-cyano-3,12-dioxo-, methyl ester
M1886.pos	0.53	0.36	0.79	Nodiff	1-Octadecyl lysophosphatidic acid
M1887.pos	-0.06	0.48	0.81	Nodiff	3-(2-Furanyl)-2-propenal
M1888.neg	1.01	0.27	1.12	Nodiff	Flecainide
M1889.pos	1.07	0.09	1.61	Nodiff	LACTULOSE
M189.pos	-0.69	0.55	0.55	Nodiff	N-Acetyl-arginine
M1890.pos	-0.53	0.18	1.25	Nodiff	Murrayamine_A
M1891.neg	1.29	0.25	1.20	Nodiff	Mongrhoside
M1892.pos	-1.29	0.01	2.07	Nodiff	Pyributicarb
M1893.neg	-0.26	0.53	0.58	Nodiff	N-(2-Phenoxyphenyl)methanesulfonamide
M1895.neg	-0.25	0.51	0.68	Nodiff	(5Z)-5-(4-Hydroxy-3-methoxybenzylidene)-1,3-thiazolidine-2,4-dione
M1896.pos	0.10	0.64	0.52	Nodiff	(2R,3R)-2-(3,4-dihydroxyphenyl)-3,5,7-trihydroxy-2,3-dihydro-4H-chromen-4-one
M1897.neg	0.40	0.36	0.93	Nodiff	3-Oxocyclobutanecarboxylic acid
M1898.pos	0.34	0.59	0.63	Nodiff	MLS002638110-01!PREDNISOLONE50-24-8
M1899.pos	1.76	0.18	1.36	Nodiff	Candidone
M19.neg	0.75	0.34	0.99	Nodiff	Quinic acid
M190.neg	-0.06	0.98	0.06	Nodiff	Azelaic acid
M1900.pos	0.38	0.39	0.91	Nodiff	5-(Furan-3-yl)-2a',5'-dihydroxy-8b'-(hydroxymethyl)-7'-methyldodecahydrospiro[furan-3,6'-naphtho[1,8-bc]furan]-2-one
M1901.pos	0.34	0.33	0.93	Nodiff	Methyl 3-[(2-thienylacetyl)amino]-2-thiophenecarboxylate

M1902.pos	-0.23	0.47	0.70	Nodiff	Cisapride
M1903.pos	-0.10	0.37	0.98	Nodiff	N-Desmethylulipristal acetate
M1904.pos	1.86	0.18	1.31	Nodiff	Antibiotic WB
M1905.neg	0.63	0.20	1.35	Nodiff	(E)-1-Hydroxy-2-methylbut-2-enyl 4-diphosphate
M1906.pos	0.41	0.47	0.75	Nodiff	Putative Phenylalanine conjugated chenodeoxycholic acid
M1907.neg	-1.28	0.18	1.28	Nodiff	4-Methylquinoline-2,7-diol
M1908.pos	-0.48	0.18	1.33	Nodiff	alpha-[3-[(Hydroxymethyl)nitrosoamino]propyl]-3-pyridinemethanol
M1909.neg	-2.09	0.40	0.86	Nodiff	2-(Butan-2-yl)-5a-hydroxy-4-(4-oxoquinazolin-3(4H)-yl)-4,5,5a,9c-tetrahydro-3H-2a,9b-diazacyclopenta[jk]fluorene-1,3(2H)-dione
M191.pos	-0.85	0.23	1.18	Nodiff	Imidazoleacetic acid
M1910.pos	2.37	0.17	1.33	Nodiff	4,8_dimethylnonanoyl_carnitine
M1911.pos	1.12	0.18	1.32	Nodiff	SUXIBUZONE
M1912.pos	0.17	0.43	0.77	Nodiff	4-[2-(2,6-dimethoxy-4-prop-2-enylphenoxy)-1-hydroxypropyl]-2-methoxyphenol
M1913.pos	0.26	0.41	0.87	Nodiff	Limonin
M1914.pos	0.39	0.37	0.87	Nodiff	Neryl_glucoside
M1915.pos	0.31	0.39	0.87	Nodiff	(3Z)-6-Hydroxy-3-(1H-imidazol-5-ylmethylidene)-12-methoxy-7a-(2-methylbut-3-en-2-yl)-7a,12-dihydro-1H,5H-imidazo[1',2':1,2]pyrido[2,3-b]indole-2,5(3H)-dione
M1916.pos	1.16	0.30	1.10	Nodiff	VPGPR_Enterostatin
M1917.pos	-2.52	0.08	1.74	Nodiff	Laurycolactone B
M1918.pos	-0.93	0.06	1.58	Nodiff	6-(alpha-D-Glucosaminyl)-1D-myo-inositol
M1919.neg	0.54	0.56	0.65	Nodiff	Asterric acid
M192.neg	-0.29	0.51	0.68	Nodiff	(S)-2-acetamido-4-amino-4-oxobutanoic acid
M1920.neg	0.64	0.55	0.66	Nodiff	Glu-Lys
M1921.neg	1.22	0.03	1.87	Nodiff	Dopamine 4-.beta.-D-glucuronide
M1922.pos	-0.87	0.14	1.38	Nodiff	(2R)-3-(tert-Butylamino)propane-1,2-diol
M1923.neg	-0.33	0.54	0.57	Nodiff	5-[(4-Chlorophenoxy)methyl]-4-methyl-4H-1,2,4-triazole-3-thiol
M1924.neg	0.49	0.52	0.58	Nodiff	(3.beta.,5.Xi.,8.alpha.,9.beta.,10.alpha.,16.beta.)-16-Hydroxykauran-3-yl .beta.-D-glucopyranoside
M1925.neg	0.38	0.41	0.83	Nodiff	2-Amino-8-(phenacylthio)-3,7-dihydropurin-6-one
M1926.pos	0.56	0.24	1.12	Nodiff	Corchoroside_A
M1927.pos	1.09	0.29	1.10	Nodiff	Capsoside_A
M1928.neg	0.97	0.40	0.85	Nodiff	Nitrofurazone
M1929.pos	0.69	0.29	1.02	Nodiff	(4OH,8Z,t18:1)_sphingosine
M193.neg	0.29	0.42	0.85	Nodiff	N-Acetylmuramic acid
M1930.pos	-0.49	0.20	1.25	Nodiff	1-Methyl-4-[2-(4-piperidinyl)ethyl]piperazine
M1931.pos	0.59	0.31	0.91	Nodiff	Lutein 5,6-epoxide
M1932.neg	-0.73	0.35	0.93	Nodiff	5a-Hydroxy-4-(4-oxoquinazolin-3(4H)-yl)-2-(propan-2-yl)-4,5,5a,9c-tetrahydro-3H-2a,9b-diazacyclopenta[jk]fluorene-1,3(2H)-dione
M1933.pos	1.40	0.26	1.18	Nodiff	Ergostane-3,6-dione
M1934.pos	-0.04	0.75	0.39	Nodiff	N-Methyl-2-piperidin-4-ylacetamide
M1935.pos	-0.43	0.78	0.28	Nodiff	Secobarbital
M1936.neg	0.92	0.16	1.43	Nodiff	Sakyomicin A
M1937.pos	-0.56	0.15	1.33	Nodiff	Flibanserin
M1938.pos	-0.07	0.46	0.64	Nodiff	Sch 210972
M1939.pos	-0.34	0.40	0.94	Nodiff	Piperlongumine
M194.pos	0.18	0.49	0.67	Nodiff	Stachydrine
M1940.neg	1.21	0.06	1.71	Nodiff	N-(2-Methylphenyl)benzenesulfonamide
M1941.pos	1.07	0.18	1.32	Nodiff	Piperochromanoic_acid
M1942.pos	0.13	0.15	1.39	Nodiff	Valganciclovir
M1943.pos	0.10	0.53	0.59	Nodiff	2,2-Dimethylpiperazine-1,4-diol

M1944.pos	0.22	0.45	0.79	Nodiff	N-Demethylmifepristone
M1945.pos	1.80	0.03	1.87	Nodiff	Cyclohexanecarboxylic acid, 1-phenyl-, 2-(4-morpholinyl)ethyl ester
M1946.pos	-0.96	0.33	0.89	Nodiff	4-Imidazolidineheptanoic acid, 3-[(2-cyclohexyl-2-hydroxyethyl)amino]-2,5-dioxo-1-(phenylmethyl)-
M1947.pos	0.36	0.51	0.68	Nodiff	15-formyl-16-hydroxy-1,2,14,17,17-pentamethyl-8-(prop-1-en-2-yl)pentacyclo[11.7.0.0?,??.0?,?.0??,??]icosane-5-carboxylic acid
M1948.pos	-0.38	0.41	0.71	Nodiff	Kukoamine A
M1949.neg	0.58	0.19	1.36	Nodiff	Oenin
M195.neg	0.37	0.28	1.11	Nodiff	Pantothenic acid
M1950.neg	-0.35	0.41	0.82	Nodiff	(1-Acetyloxy-3-hydroxy-6,8a-dimethyl-7-oxo-3-propan-2-yl-2,3a,4,8-tetrahydro-1H-azulen-4-yl) 4-hydroxybenzoate
M1951.pos	2.44	0.16	1.35	Nodiff	Lactariamide_B
M1952.neg	-0.52	0.39	0.82	Nodiff	3-Hydroxy-5-((3-hydroxy-6-methyl-5-(pentofuranosyloxy)octanoyl)oxy)-6-methyloctanoic acid
M1953.pos	-0.02	0.94	0.13	Nodiff	2-Piperazinecarboxamide
M1954.pos	1.74	0.26	1.10	Nodiff	Rhusflavanone
M1955.pos	0.41	0.38	0.85	Nodiff	Ethyl nitroacetate
M1956.neg	0.99	0.32	1.02	Nodiff	1H-Indole-4-carboxamide, N-[(1,2-dihydro-4,6-dimethyl-2-oxo-3-pyridinyl)methyl]-3-methyl-1-[(1S)-1-methylpropyl]-6-[6-(1-piperazinyl)-3-pyridinyl]-
M1957.pos	0.41	0.63	0.44	Nodiff	betulin
M1958.neg	1.00	0.12	1.43	Nodiff	(2S,3R)-2,3,4-Trihydroxybutyl 2,6-di-O-acetyl-3-O-dodecanoyl-.beta.-D-mannopyranoside
M196.pos	0.19	0.58	0.58	Nodiff	Butyrylcarnitine (Car(4:0))
M1960.neg	-0.27	0.55	0.59	Nodiff	Citrusin V
M1961.neg	0.73	0.28	1.08	Nodiff	13-Hydroxy-6-(1-hydroxyethyl)-12,14-dimethyl-3-methylidene-15-nonyl-9-(propan-2-yl)-1-oxa-4,7,10-triazacyclopentadecane-2,5,8,11-tetrone
M1962.pos	0.91	0.37	0.86	Nodiff	(25R)-3alpha,7alpha,12alpha,24S-tetrahydroxy-5beta-cholestan-26-oic_acid
M1963.neg	-0.11	0.93	0.11	Nodiff	3,5,9-Trioxa-4-phosphatetracosan-1-aminium, 7-(acetyloxy)-24-carboxy-4-hydroxy-N,N,N-trimethyl-, inner salt, 4-oxide, (R)-
M1964.pos	-0.50	0.63	0.51	Nodiff	16(17)-EpDPE
M1965.pos	0.01	0.87	0.25	Nodiff	DOCOSANOL
M1966.pos	-0.07	0.47	0.78	Nodiff	2,6-ditert-butyl-4-methylphenol
M1967.pos	5.24	0.27	1.09	Nodiff	Ethyl 3-oxo-3-(2,3,4,5-tetrafluorophenyl)propanoate
M1968.pos	0.55	0.27	1.09	Nodiff	N-[(3s,5s,7s)-Adamantan-1-yl]-1-(4-fluorobenzyl)-1H-indazole-3-carboxamide
M1969.neg	2.11	0.96	0.06	Nodiff	Angoroside A
M197.pos	0.31	0.57	0.50	Nodiff	Cholic acid
M1970.neg	1.84	0.07	1.72	Nodiff	Gly-Phe-.beta.-naphthylamide
M1971.pos	-0.41	0.10	1.61	Nodiff	3,3'-(2,4,6-Trioxo-1,3,5-triazinane-1,3-diyl)dipropanenitrile
M1972.pos	-0.12	0.66	0.55	Nodiff	2-Bromo-6-chlorobenzoic acid
M1973.neg	1.23	0.31	1.03	Nodiff	4-(Methylmercapto)phenol
M1974.neg	0.93	0.49	0.71	Nodiff	9(S)-HOTrE
M1975.pos	-0.29	0.64	0.48	Nodiff	L-Valine, N-[(1-pentyl-1H-indazol-3-yl)carbonyl]-, methyl ester
M1976.pos	0.11	0.96	0.07	Nodiff	N6-Carbamimidoyl-N2-((4E,6E,12E,14E)-3-hydroxy-2-(hydroxymethyl)-8,10,16-trimethyloctadeca-4,6,12,14-tetraenoyl)lysine

M1977.pos	1.54	0.10	1.58	Nodiff	.alpha.-Hexylcinnamaldehyde
M1978.pos	-0.01	0.98	0.08	Nodiff	10'-apo-beta-carotenal
M1979.neg	0.47	0.39	0.90	Nodiff	1-Stearoyl-2-docosahexaenoyl-sn-glycero-3-phospho-(1'-sn-glycerol)
M198.neg	-1.36	0.06	1.83	Nodiff	2-oxindole-3-acetate
M1980.neg	-0.84	0.33	1.11	Nodiff	Scrophularoside A8
M1981.pos	2.16	0.13	1.50	Nodiff	Phellamurin
M1982.neg	-0.44	0.15	1.29	Nodiff	(1aS,1bS,2S,5aR,6S,6aS)-6-((3-O-Acetyl-6-deoxy-2-O-((2E)-3-(4-hydroxyphenyl)prop-2-enoyl)-.alpha.-L-mannopyranosyl)oxy)-1a-(hydroxymethyl)-1a,1b,2,5a,6,6a-hexahydrooxireno[4,5]cyclopenta[1,2-c]pyran-2-yl 2-O-acetyl-.beta.-D-glucopyranoside
M1983.pos	0.85	0.19	1.39	Nodiff	Dienestrol diacetate
M1984.pos	-0.43	0.11	1.53	Nodiff	Zearalenone
M1985.neg	1.34	0.02	2.05	Nodiff	1H-Indole-1-propanoic acid, 3-(1-naphthalenylcarbonyl)-
M1986.pos	-0.89	0.21	1.27	Nodiff	Podophyllotoxin
M1987.pos	-2.41	0.13	1.54	Nodiff	3-(2,6-Dimethyl-4-morpholinyl)-N-phenylpropanamide
M1988.neg	0.02	0.34	0.97	Nodiff	Asp-His
M1989.pos	0.00	0.71	0.38	Nodiff	N2-(1-Oxo-4-phenylbutyl)-L-glutamine
M199.pos	0.91	0.14	1.41	Nodiff	LPC(16:0)
M1990.pos	0.82	0.19	1.38	Nodiff	3-Hydroxy-10'-apo-b,y-carotenal
M1991.pos	0.25	0.42	0.85	Nodiff	Termitomycamide E
M1992.neg	0.11	0.92	0.17	Nodiff	3-Chloro-5-fluoro-4-methoxybenzoic acid
M1993.pos	-1.68	0.78	0.30	Nodiff	2-[4-[3-[3,4-dihydroxy-4-(hydroxymethyl)oxolan-2-yl]oxy-4,5-dihydroxy-6-(hydroxymethyl)oxan-2-yl]oxyphenyl]-7-hydroxy-2,3-dihydrochromen-4-one
M1994.pos	0.67	0.17	1.26	Nodiff	LysoPE(0:0/18:0)
M1995.pos	0.01	0.93	0.11	Nodiff	Minoxidil
M1996.neg	0.83	0.46	0.76	Nodiff	Cys-Gly-Cys
M1997.pos	-0.51	0.08	1.60	Nodiff	3-Methoxy-5-(2-phenylethyl)phenol
M1998.neg	-0.04	0.54	0.64	Nodiff	N1-(2-Hydroxyethyl)flurazepam
M1999.neg	-0.30	0.89	0.13	Nodiff	Acetamide, N-(6-methyl-2-benzothiazolyl)-2-[[3,4,6,7-tetrahydro-3-(2-methoxyphenyl)-4-oxothieno[3,2-d]pyrimidin-2-yl]thio]-
M2.neg	0.21	0.50	0.62	Nodiff	alpha-Muricholic acid
M20.pos	-0.29	0.87	0.18	Nodiff	3'-O-methylcytidine
M200.pos	0.07	0.90	0.20	Nodiff	Cytosine
M2000.neg	2.34	0.15	1.40	Nodiff	Fenoldopam
M2001.neg	-0.54	0.29	0.99	Nodiff	epiyangambin
M2002.pos	-0.17	0.58	0.62	Nodiff	Mintsulfide
M2003.neg	-1.51	0.66	0.53	Nodiff	4-(4-Mercaptophenoxy)phenyl hydrosulfide
M2004.neg	-0.16	0.73	0.28	Nodiff	8-Oxo-9-(3,4,5-trimethoxyphenyl)-5,5a,6,8,8a,9-hexahydrofuro[3',4':6,7]naphtho[2,3-d][1,3]dioxol-5-yl hexopyranoside
M2005.pos	0.88	0.59	0.63	Nodiff	Junipediol A 8-glucoside
M2006.pos	-0.52	0.26	0.98	Nodiff	Benzoic acid, 2-hydroxy-4-[[2-[[(4-methylphenyl)sulfonyl]oxy]acetyl]amino]-
M2007.neg	0.50	0.43	0.89	Nodiff	Vaccinoside
M2008.neg	-0.21	0.52	0.53	Nodiff	[(4-Methylbenzyl)sulfanyl]acetic acid
M2009.pos	0.78	0.13	1.50	Nodiff	N-Desmethylclomipramine
M201.neg	-0.46	0.25	1.13	Nodiff	L-Fucitol
M2010.pos	-0.05	0.71	0.36	Nodiff	Iressa
M2011.pos	0.19	0.43	0.75	Nodiff	Ethyl 2-amino-5-phenyl-3-thiophenecarboxylate
M2012.pos	1.67	0.11	1.49	Nodiff	7,8-Didehydroastaxanthin
M2013.pos	1.23	0.03	1.95	Nodiff	3-(1-(Cyclohexylmethyl)-1H-indazole-3-carboxamido)-2,2-dimethylsuccinic acid

M2014.pos	0.98	0.12	1.44	Nodiff	Umbelliferyl arachidonate
M2015.pos	-0.53	0.12	1.56	Nodiff	Hydrocortamate
M2016.neg	0.22	0.42	0.91	Nodiff	2-Furyl(oxo)acetic acid
M2017.pos	-0.27	0.95	0.08	Nodiff	Zeinoxanthin
M2018.neg	1.20	0.28	1.11	Nodiff	.beta.-D-Glucopyranoside, 4-[(1R,3aR,4S,6aS)-6a-(acetyloxy)tetrahydro-4-(4-hydroxy-3-methoxyphenyl)-1H,3H-furo[3,4-c]furan-1-yl]-2-methoxyphenyl
M2019.pos	-0.45	0.16	1.31	Nodiff	Thiabendazole
M202.neg	0.80	0.18	1.29	Nodiff	Phe-Val
M2021.pos	0.83	0.13	1.39	Nodiff	3-[3-(Dimethylamino)propyl]-4-hydroxy-N-[4-(4-pyridyl)phenyl]benzamide
M2022.pos	-1.18	0.01	1.93	Nodiff	Glycyl-L-4-hydroxyproline
M2023.pos	1.12	0.22	1.24	Nodiff	Betamethasone 17-valerate
M2024.pos	0.79	0.27	1.16	Nodiff	17-Oxodexamethasone
M2025.pos	-0.09	0.66	0.53	Nodiff	5-(10-Nonadecenyl)-1,3-benzenediol
M2026.pos	-0.05	1.00	0.06	Nodiff	PE(20:3(8Z,11Z,14Z)/0:0)
M2027.neg	-0.71	0.39	0.81	Nodiff	(6-Methyl-3-oxo-2,3-dihydro-4H-1,4-benzoxazin-4-yl)acetic acid
M2028.pos	-0.54	0.40	0.84	Nodiff	(3S,3'R,4xi)-beta,beta-Carotene-3,3',4-triol
M2029.pos	-0.46	0.91	0.19	Nodiff	PE(20:1(11Z)/14:1(9Z))
M203.neg	0.80	0.18	1.29	Nodiff	L-Valyl-L-phenylalanine
M2030.neg	0.88	0.15	1.42	Nodiff	apigenin 6,8-digalactoside
M2031.neg	0.21	0.54	0.71	Nodiff	Verbaspinoside
M2032.pos	-0.28	0.82	0.22	Nodiff	Ramipril
M2033.pos	1.35	0.02	1.94	Nodiff	Furaltadone
M2034.neg	-0.12	0.47	0.86	Nodiff	Levofloxacin
M2035.neg	-1.05	0.10	1.54	Nodiff	Decuroside IV
M2036.neg	-1.31	0.17	1.52	Nodiff	5,7-Dihydroxy-1-((2-O-((2E)-3-(4-hydroxyphenyl)prop-2-enoyl)hexopyranosyl)oxy)-7-methyl-1,4a,5,6,7,7a-hexahydrocyclopenta[c]pyran-4-carboxylic acid
M2037.neg	0.91	0.16	1.40	Nodiff	5-Hydroxy-6,8-dimethoxy-2-oxo-2H-chromen-7-yl .beta.-D-glucopyranoside
M2038.neg	-0.02	0.47	0.82	Nodiff	Hydroxytolbutamide
M2039.pos	0.15	0.52	0.65	Nodiff	Ethyl 5-amino-1,2,3-thiadiazole-4-carboxylate
M204.neg	0.61	0.36	0.96	Nodiff	N-Acetylphenylalanine
M2040.pos	-0.23	0.40	0.95	Nodiff	Auroxanthin
M2041.pos	0.91	0.16	1.25	Nodiff	LysoPE(0:0/18:3(9Z,12Z,15Z))
M2042.pos	1.65	0.03	1.82	Nodiff	Carebastine
M2043.pos	1.05	0.14	1.36	Nodiff	Melilotoside_D
M2044.pos	-0.11	1.00	0.05	Nodiff	Clobetasol_propionate
M2045.neg	-0.43	0.12	1.48	Nodiff	4-[(2-Chloroethyl)amino]-1,2,5-thiadiazole-3-carboxylic acid
M2046.pos	1.88	0.09	1.59	Nodiff	Calycanthidine
M2047.pos	-1.19	0.03	1.85	Nodiff	Apo-10'-violaxanthal
M2048.neg	0.06	0.86	0.08	Nodiff	Asperphenalenone E
M2049.pos	0.48	0.30	0.98	Nodiff	Urea, N-[2-methyl-5-(1-piperidinylsulfonyl)-3-furanyl]-N'-phenyl-
M205.neg	-1.18	0.87	0.12	Nodiff	2'-Deoxyuridine
M2050.neg	0.54	0.21	1.25	Nodiff	Phlorobenzophenone
M2051.neg	-0.01	0.83	0.20	Nodiff	2-(4-Nitrobenzoyl)-N-phenylhydrazinecarboxamide
M2052.neg	0.56	0.64	0.43	Nodiff	N-(5-Chloropyridin-2-yl)-5-propylthiophene-3-carboxamide
M2053.pos	1.04	0.06	1.59	Nodiff	Biotin-XX hydrazide
M2054.pos	-0.87	0.42	0.85	Nodiff	PE(22:2(13Z,16Z)/14:1(9Z))
M2056.neg	-0.99	0.18	1.48	Nodiff	trifolirhizin
M2057.neg	-0.79	0.04	1.85	Nodiff	2-Benzoyl-3,5-dihydroxyphenyl .beta.-D-glucopyranoside
M2058.neg	0.55	0.29	1.08	Nodiff	4-(4-Hydroxyphenyl)butan-2-yl 6-O-((4.Xi.)-.alpha.-L-threo-pentofuranosyl)

					-.beta.-D-glucopyranoside
M2059.neg	0.10	0.81	0.25	Nodiff	Methyl 2-(4a,6,10a-trihydroxy-1-methyl-5,10-dioxo-3,4-dihydro-1H-benzo[g]isochromen-3-yl) acetate
M206.pos	0.92	0.87	0.09	Nodiff	5'-Methylthioadenosine
M2060.pos	0.68	0.65	0.54	Nodiff	N2,N4-Bis(2-methoxyethyl)-2,4-pyridinedicarboxamide
M2061.pos	0.37	0.18	1.36	Nodiff	1-Ethyl-5-fluoro-1H-indole-3-carbaldehyde
M2062.pos	1.93	0.06	1.63	Nodiff	Spirolide_E
M2063.neg	1.06	0.18	1.40	Nodiff	Bis(2-hydroxy-1-naphthyl)disulfide
M2064.neg	0.22	0.53	0.68	Nodiff	Alternariol
M2065.neg	0.11	0.44	0.77	Nodiff	3-(Methylsulfanyl)-4-oxo-4,5,6,7-tetrahydro-2-benzothiophene-1-carboxylic acid
M2066.neg	0.75	0.34	1.00	Nodiff	2-Hydroxy-2-[2-[(Z)-2-hydroxy-2,6-dimethyl-8-(7-oxofuro[3,2-g]chromen-4-yl)oxyoct-6-en-3-yl]oxy-2-oxoethyl]butanedioic acid
M2067.pos	0.86	0.21	1.13	Nodiff	5-Bromo-1-butyl-6-fluorobenzotriazole
M2069.neg	0.42	0.35	1.01	Nodiff	Doxycycline
M207.neg	1.11	0.02	1.89	Nodiff	2-Hydroxypalmitic acid
M2070.pos	0.14	0.67	0.37	Nodiff	Dyphylline
M2071.pos	-1.95	0.25	1.19	Nodiff	1-[2-[(4-Chlorophenyl)phenylmethoxy]ethyl]piperidine
M2072.pos	0.19	0.57	0.60	Nodiff	Ramiprilat
M2073.pos	-0.29	0.97	0.02	Nodiff	Geldanamycin, 17-demethoxy-17-[[2-(dimethylamino)ethyl]amino]-
M2074.pos	-0.32	0.44	0.81	Nodiff	2-(Ethylsulfinylmethyl)phenyl_methylcarbamate
M2075.neg	1.10	0.26	1.13	Nodiff	Decoyinine
M2076.pos	-1.23	0.28	0.99	Nodiff	5-(10,13-Nonadecadienyl)-1,3-benzenediol
M2077.neg	-0.32	0.74	0.36	Nodiff	2-[3-[3-Hydroxy-4-(4-hydroxyphenyl)-2,5-dimethoxyphenyl]-5-oxo-2H-furan-2-yl]acetic acid
M2078.neg	0.67	0.22	1.25	Nodiff	2-({[5-(Thiophen-2-yl)-1,2-oxazol-3-yl]methyl}sulfanyl)acetic acid
M2079.neg	-0.35	0.23	1.15	Nodiff	Multifloroside
M208.pos	-2.25	0.24	1.06	Nodiff	2-Piperidone
M2080.pos	-0.61	0.21	1.37	Nodiff	Fridamycin A
M2081.pos	0.48	0.22	1.23	Nodiff	(2E,6E,11E,13E)-18-(2,6-Dioxopiperidin-4-yl)-9-hydroxy-8-methoxy-10,12,14-trimethyl-15-oxooctadeca-2,6,11,13-tetraenoic acid
M2082.pos	-0.27	0.51	0.65	Nodiff	Tiagabine
M2083.pos	1.09	0.07	1.65	Nodiff	1-Piperazinamine, N-[(3,5-dimethyl-1-phenyl-1H-pyrazol-4-yl)methylene]-4-(phenylmethyl)-
M2084.neg	-0.03	0.96	0.08	Nodiff	8-O-4-Hydroxycinnamoylharpagide
M2085.pos	0.25	0.63	0.43	Nodiff	2-(4-Nitrophenyl)acetohydrazide
M2086.pos	-0.98	0.01	1.98	Nodiff	2-Phthalimidoglutaric acid
M2087.pos	-0.10	0.62	0.41	Nodiff	N-Acetylanonaine
M2088.neg	0.12	0.45	0.77	Nodiff	4-((1E)-3-(2,4-Dihydroxyphenyl)-3-oxoprop-1-en-1-yl)phenyl 6-O-((2E)-3-(4-hydroxyphenyl)prop-2-enoyl)-.beta.-D-glucopyranoside
M2089.pos	1.41	0.10	1.49	Nodiff	ACARBOSE
M209.neg	-0.39	0.50	0.78	Nodiff	3-Methoxy-4-hydroxyphenylglycol sulfate
M2090.pos	0.60	0.49	0.69	Nodiff	3-Deacetylsalannin
M2091.pos	-0.26	0.93	0.16	Nodiff	7-Hydroxy-4-(3-trifluoromethylphenyl)coumarin
M2092.pos	-0.46	0.21	1.32	Nodiff	alpha-TOCHOPHERYL_ACETATE
M2093.pos	-0.14	0.73	0.35	Nodiff	N-Arachidonoyldopamine
M2094.neg	-0.59	0.44	0.83	Nodiff	Catechin
M2095.pos	2.25	0.36	0.87	Nodiff	Methyl {4-[(difluoromethyl)sulfonyl]phenyl}carba

					mate
M2096.pos	0.75	0.23	1.20	Nodiff	Galactopinitol_A
M2097.pos	-0.13	0.79	0.31	Nodiff	N-(1H-Tetraazol-5-yl)decanamide
M2098.pos	0.48	0.17	1.29	Nodiff	Isopeonidin_3-rutinoside
M2099.pos	1.41	0.96	0.09	Nodiff	(4S,8R)-8,9-Dihydroxy-p-menth-1(6)-en-2-one
M21.pos	0.49	0.21	1.16	Nodiff	Glycitin
M210.neg	0.07	0.64	0.44	Nodiff	Phytanic acid
M2100.pos	0.27	0.36	0.92	Nodiff	(10E)-6,10-Dimethyl-3-methylidene-2,7-dioxo-2,3,3a,4,5,6,7,11a-octahydro-6,9-epoxycyclodeca[b]furan-4-yl (2E)-4-(acetyloxy)-2-methylbut-2-enoate
M2101.neg	0.91	0.60	0.59	Nodiff	4-Hydroxy-3-oxo-1,3-dihydronaphtho[2,3-c]furan-5-yl .beta.-D-glucopyranoside
M2102.pos	0.04	0.77	0.27	Nodiff	Huperzine A
M2103.pos	-1.47	0.02	1.90	Nodiff	Neomangiferin
M2104.pos	-0.19	0.46	0.70	Nodiff	5,6,2'-Trimethoxyflavone
M2105.pos	0.21	0.55	0.70	Nodiff	Dibenzyl N,N-diethylphosphoramidite
M2106.pos	1.27	0.11	1.54	Nodiff	Oxybutynin N-oxide
M2107.pos	0.79	0.12	1.43	Nodiff	4H-Thieno[3,2-b]pyrrole-5-carboxamide, 2-chloro-N-[2-(3,4-diethoxyphenyl)ethyl]-4-methyl-
M2108.pos	1.55	0.10	1.44	Nodiff	Pyrrocidine B
M2109.pos	0.13	0.64	0.43	Nodiff	N-(4-Methoxyphenyl)ethanethioamide
M211.pos	0.36	0.51	0.68	Nodiff	L-tyrosine-methyl-ester
M2110.pos	-1.23	0.74	0.36	Nodiff	3-[1,1-bis(4-hydroxyphenyl)-3-oxo-3-(2,4,6-trihydroxyphenyl)propan-2-yl]-5,7-dihydroxychromen-4-one
M2111.pos	1.12	0.31	1.05	Nodiff	4-Methoxyphlorizin
M2112.neg	0.16	0.40	0.94	Nodiff	Chamanetin
M2113.neg	0.28	0.55	0.74	Nodiff	Resazurin
M2114.pos	-0.23	0.59	0.43	Nodiff	Communesin-B
M2115.neg	-0.66	0.41	0.75	Nodiff	(4-Bromo-2-tert-butylphenoxy)acetic acid
M2116.pos	-0.13	0.67	0.48	Nodiff	5-(2-Methylbutan-2-yl)-4,5,6,7-tetrahydro-2H-indazole-3-carbohydrazide
M2117.pos	1.40	0.16	1.41	Nodiff	Dimebon
M2118.pos	2.03	0.16	1.38	Nodiff	1-Cyclohexyl-N-(3-methylphenyl)-5-oxo-3-pyrrolidinecarboxamide
M2119.pos	0.09	0.55	0.50	Nodiff	1H-Indazole-3-carboxamide, N-[1-(aminocarbonyl)-2,2-dimethylpropyl]-1-pentyl-
M212.neg	0.99	0.67	0.51	Nodiff	Tauroursodeoxycholic acid
M2120.neg	-1.72	0.00	2.20	Nodiff	N-Benzoylbenzenesulfonamide
M2121.neg	0.12	0.97	0.06	Nodiff	Diethyl 5-methyl-3-phenyl-1H-pyrrole-2,4-dicarboxylate
M2122.pos	-0.29	0.24	1.11	Nodiff	6-Methyl-3-phenylpyrimido[5,4-e][1,2,4]triazine-5,7(6H,8H)-dione
M2123.pos	-0.84	0.17	1.26	Nodiff	Annoglabasin_C
M2124.pos	0.01	0.99	0.01	Nodiff	1-(1-Ethylpropyl)-4-(2,3,4-trimethoxybenzyl)piperazine
M2125.neg	-0.02	0.54	0.61	Nodiff	2-Hydroxy-4-(trifluoromethyl)pyrimidine-5-carboxylic acid
M2126.neg	-0.65	0.39	0.90	Nodiff	2-(Trifluoromethyl)pyrimidine-4-carboxylic acid
M2127.neg	-0.79	0.97	0.03	Nodiff	6-Hydroxy-1-((2-O-((2E)-3-(4-hydroxyphenyl)prop-2-enoyl)hexopyranosyl)oxy)-7-methylidene-1,4a,5,6,7,7a-hexahydrocyclopenta[c]pyran-4-carboxylic acid
M2128.pos	1.32	0.83	0.26	Nodiff	METHYL VANILLATE
M2129.pos	-0.05	0.45	0.84	Nodiff	Caylin-2
M213.neg	-0.41	0.06	1.65	Nodiff	Ribitol
M2130.pos	0.13	0.65	0.47	Nodiff	4-(4-Hydroxy-2,6,6-trimethylcyclohex-1-en-1-yl)butan-2-yl .beta.-D-glucopyranoside

M2131.pos	0.34	0.40	0.90	Nodiff	4-(Acetylamino)-N-(2,6-dimethylphenyl)benzamide
M2132.pos	-0.10	0.99	0.04	Nodiff	5,7-DIHYDROXYISOFLAVONE
M2133.pos	1.00	0.12	1.52	Nodiff	Pracinostat
M2134.pos	0.79	0.38	0.92	Nodiff	7-O-Methyllicoricidin
M2135.neg	-0.23	0.80	0.25	Nodiff	4-Phenyl-2-sulfanyl-3,4-dihydro-5H-indeno[1,2-d]pyrimidin-5-one
M2136.neg	-0.61	0.35	0.90	Nodiff	3-[[4-(4-Chlorophenyl)piperazino]methyl]-1H-pyrrolo[2,3-b]pyridine
M2137.pos	-0.46	0.06	1.73	Nodiff	4,5-dihydroxy-9,10-dioxo-9,10-dihydroanthracene-2-carboxylic acid
M2138.neg	-0.44	0.75	0.30	Nodiff	4-(Thiophen-2-yl)-6-(trifluoromethyl)pyrimidin-2-ol
M2139.neg	-1.05	0.21	1.16	Nodiff	3-[5-(Trifluoromethyl)-1,2,4-oxadiazol-3-yl]benzoic acid
M214.neg	-0.41	0.06	1.65	Nodiff	Xylitol
M2140.pos	0.37	0.95	0.20	Nodiff	MUPIROCIN
M2141.neg	-0.92	0.23	1.16	Nodiff	Glycine, N-[2-(3,5-difluorophenyl)acetyl]-L-alanyl-2-phenyl-, 1,1-dimethylethyl ester, (2S)-
M2142.neg	2.62	0.13	1.54	Nodiff	Tetracycline
M2143.neg	0.19	0.51	0.78	Nodiff	6a-Hydroxy-9,10a-dimethoxy-2-(prop-1-en-2-yl)-1,2,12,12a-tetrahydrochromeno[3,4-b]furo[2,3-h]chromene-6,8(6aH,10aH)-dione
M2144.pos	-0.56	0.44	0.86	Nodiff	Frovatriptan
M2145.neg	0.90	0.07	1.67	Nodiff	Marbofloxacin
M2146.neg	0.46	0.34	0.99	Nodiff	2-Acetyl-7-hydroxy-1-(4-hydroxybenzyl)-1,2,3,4-tetrahydroisoquinolin-8-yl 6-O-(3,4-dihydroxy-4-(hydroxymethyl)tetrahydrofuran-2-yl)hexopyranoside
M2147.neg	0.62	0.47	0.76	Nodiff	Oxymorphone 3-.beta.-D-glucuronide
M2148.neg	0.66	0.55	0.66	Nodiff	2-Hydroxy-4-(prop-2-en-1-yl)phenyl 6-O-.beta.-D-glucopyranosyl-.beta.-D-glucopyranoside
M2149.neg	-0.42	0.31	0.88	Nodiff	4-((3R,4S)-4-Hydroxy-4-(4-hydroxy-3-methoxybenzyl)-3-(hydroxymethyl)tetrahydrofuran-2-yl)-2-methoxyphenyl .beta.-D-glucopyranoside
M215.pos	0.07	0.88	0.21	Nodiff	beta-Guanidinopropionic acid
M2150.pos	0.07	0.51	0.63	Nodiff	Anacine
M2151.pos	1.39	0.08	1.59	Nodiff	Cafestol acetate
M2152.pos	-0.14	0.70	0.47	Nodiff	7-Hydroxy-3-phenyl-2-(trifluoromethyl)-4H-chromen-4-one
M2153.neg	0.04	0.44	0.86	Nodiff	Norfludiazepam
M2154.neg	-0.70	0.05	1.66	Nodiff	2-Methoxy-5-(methylsulfonyl)benzoic acid
M2155.pos	0.22	0.33	1.01	Nodiff	7-[2,6-dimethyl-8-(2-methylbutanoyloxy)-1,2,6,7,8,8a-hexahydronaphthalen-1-yl]-3,5-dihydroxyheptanoic acid
M2156.pos	0.29	0.40	0.81	Nodiff	12-oxo-LTB4
M2157.neg	0.57	0.69	0.47	Nodiff	Cyclo(phenylalanyltryptophyl)
M2158.pos	0.64	0.24	1.11	Nodiff	Phaseollidin_hydrate
M2159.pos	1.84	0.10	1.49	Nodiff	Primaquine
M216.pos	-0.07	0.99	0.03	Nodiff	MeAIB
M2160.pos	-0.56	0.05	1.70	Nodiff	Tripelenamine
M2161.pos	0.95	0.33	0.94	Nodiff	Methanone, 1,1'-[2-(phenylamino)-1,4-phenylene]bis[1-[4-(1-pyrrolidinyl)-1-piperidinyl]-
M2162.pos	-1.29	0.53	0.68	Nodiff	Hericenone_B
M2163.pos	-2.73	0.13	1.47	Nodiff	Eduline
M2164.pos	-0.06	0.88	0.18	Nodiff	Tebupirimfos
M2165.pos	0.23	0.50	0.75	Nodiff	Etoposide
M2166.pos	0.38	0.38	0.89	Nodiff	Carbamic acid, N-10-undecyn-1-yl-, 3'-(aminocarbonyl)[1,1'-biphenyl]-3-yl ester
M2167.pos	-0.17	0.49	0.65	Nodiff	Dehydronifedipine

M2168.neg	0.30	0.41	0.85	Nodiff	1-O-trans-cinnamoyl-beta-D-glucopyranose
M2169.neg	-1.10	0.16	1.34	Nodiff	4-(7-Hydroxy-3-(hydroxymethyl)-5-(3-hydroxypropyl)-2,3-dihydro-1-benzofuran-2-yl)-2-methoxyphenyl 6-deoxy-.alpha.-D-mannopyranoside
M217.pos	-0.36	0.69	0.45	Nodiff	Ornithine
M2170.pos	0.01	0.88	0.23	Nodiff	3-Hexen-1-ol O-.beta.-D-glucopyranoside
M2171.pos	0.03	0.47	0.79	Nodiff	Necatorine
M2172.pos	-0.35	0.29	0.97	Nodiff	Kipukasin D
M2173.neg	-0.21	0.55	0.52	Nodiff	Propylpyrazoletriol
M2174.pos	0.67	0.62	0.56	Nodiff	1-(5,10-Dioxo-2,3,5a,6,7,8-hexahydro-1H-dipyrrolo[1,2-d:1',2'-f]pyrazin-10a-yl)propan-2-yl carbamate
M2175.pos	0.44	0.17	1.30	Nodiff	12-Deoxywithastramonolide
M2176.neg	0.41	0.49	0.67	Nodiff	4-[[5-Amino-1-(2,6-difluorobenzoyl)-1,2,4-triazol-3-yl]amino]benzenesulfonamide
M2177.neg	-0.60	0.46	0.68	Nodiff	2-(4,5-Dihydroxy-2-methylphenyl)-4-hydroxy-6-methoxybenzoic acid
M2178.pos	0.66	0.17	1.32	Nodiff	1H-Indazole-3-carboxamide, N-[(1S)-1-(aminocarbonyl)-2-methylpropyl]-1-pentyl-
M2179.pos	1.75	0.10	1.65	Nodiff	4-Piperidinecarboxylic acid, 1-[[4-(acetylamino)phenyl]methyl]-4-(2-phenylethyl)-, ethyl ester
M218.pos	-0.07	0.95	0.07	Nodiff	Glu-Arg
M2180.neg	1.14	0.05	1.78	Nodiff	Secologanoside
M2181.pos	0.34	0.66	0.50	Nodiff	(7R*,8R*)-3-Methoxy-3',4,7,9,9'-pentahydroxy-8,4'-oxyneolignan_4-xyloside
M2182.pos	-0.03	0.92	0.12	Nodiff	7-(Trifluoromethyl)-1H-indole-2,3-dione
M2183.neg	-0.63	0.35	0.99	Nodiff	1-(4-Chlorobenzyl)-5-oxo-pyrrolidine-3-carboxylic acid
M2184.pos	-0.23	0.17	1.34	Nodiff	N,N-Dimethyl-2,2-diphenylethanamine
M2185.pos	1.10	0.10	1.59	Nodiff	Corticosterone 21-acetate
M2186.neg	0.19	0.43	0.80	Nodiff	(2E)-1,3-Bis(3-hydroxyphenyl)-2-propen-1-one
M2187.neg	-0.36	0.35	0.91	Nodiff	5,6-Difluoro-1H-indazole-3-carboxylic acid
M2188.pos	-0.85	0.10	1.48	Nodiff	5.alpha.-Dihydrocortisol
M2189.pos	1.78	0.01	1.94	Nodiff	1-Piperazinecarboxamide, N-phenyl-4-(2-quinolinylmethyl)-
M219.pos	-0.93	0.06	1.62	Nodiff	Saccharopine
M2190.pos	-0.35	0.43	0.85	Nodiff	Baclofen
M2191.pos	1.29	0.08	1.62	Nodiff	Neuroprotectin_D1
M2192.neg	1.15	0.14	1.50	Nodiff	1,8-Dihydroxy-9-oxo-2,3-dihydro-1H-cyclopenta[b]chromene-6-carboxylic acid
M2193.pos	0.03	0.82	0.19	Nodiff	2-Methylthiophene-3-carbaldehyde
M2194.neg	0.17	0.48	0.71	Nodiff	2-[(6-Oxido-6H-dibenzo[c,e][1,2]oxaphosphinin-6-yl)methyl]succinic acid
M2195.pos	0.65	0.08	1.52	Nodiff	biochanin A
M2196.neg	-1.39	0.92	0.06	Nodiff	3,4-Dihydroxy-4-(4-methoxyphenyl)-1,3-dihydroquinolin-2-one
M2197.neg	1.66	0.06	1.83	Nodiff	2-[4-(Trifluoromethoxy)phenyl]cyclopropanecarboxylic acid
M2198.pos	-1.01	0.14	1.37	Nodiff	1-(2-Cyanophenyl)piperazine
M2199.neg	0.65	0.13	1.54	Nodiff	Amidepsine F
M22.neg	-0.46	0.29	0.97	Nodiff	Pyruvate
M220.pos	0.08	0.89	0.12	Nodiff	Carnosine
M2200.pos	-0.02	0.58	0.60	Nodiff	Isoxaben
M2201.pos	-0.56	0.39	0.93	Nodiff	Semagacestat
M2202.pos	-0.17	0.49	0.66	Nodiff	4-(3-Nitrophenoxy)benzoic acid
M2203.neg	-0.44	0.21	1.13	Nodiff	[(6R,7R)-7-Hydroxy-7-methyl-8-oxo-3-[(E)-prop-1-enyl]-5,6-dihydro-1H-isochromen-6-yl] 3,6-dihydroxy-4-methoxy-2-methylbenzoat

					e
M2204.neg	-0.76	0.12	1.46	Nodiff	Methyl 4-(benzyloxy)-1H-indole-2-carboxylate
M2205.neg	0.42	0.31	1.06	Nodiff	15-Oxoete
M2206.pos	0.08	0.44	0.83	Nodiff	Ethyl 4-[(4-chlorobutanoyl)amino]benzoate
M2207.pos	-0.44	0.17	1.29	Nodiff	2,6-Diamino-1H-s-triazine-4-thione
M2208.pos	2.25	0.11	1.57	Nodiff	N-[2-(4-Prenyloxyphenyl)ethyl]tiglamide
M2209.neg	-0.16	0.94	0.05	Nodiff	2-(3-Chlorophenyl)-4-quinolinecarboxylic acid
M221.pos	0.22	0.81	0.22	Nodiff	Anserine
M2210.neg	-0.03	0.45	0.75	Nodiff	Tafluprost (free acid)
M2211.neg	1.13	0.29	1.05	Nodiff	Isradipine
M2212.pos	1.11	0.81	0.18	Nodiff	Okaramine R
M2213.pos	0.08	0.86	0.29	Nodiff	(E,E)-Futoamide
M2214.neg	0.68	0.17	1.30	Nodiff	3'-Hydroxyflavanone
M2215.pos	-0.44	0.38	0.87	Nodiff	2-Propenethioamide, 2-cyano-3-(3,4-dihydroxyphenyl)-, (2E)-
M2216.neg	-0.32	0.17	1.30	Nodiff	3-Hydroxy-5-methoxy-3-(methoxycarbonyl)-5-oxopentanoic acid
M2217.pos	1.13	0.27	1.09	Nodiff	Adiphenine
M2218.pos	0.33	0.57	0.66	Nodiff	4H-Naphtho[1,2-b]pyran-4-one, 2-(4-morpholinyl)-
M2219.pos	0.25	0.46	0.78	Nodiff	Caryophyllene_epoxide
M222.pos	-1.84	0.03	1.89	Nodiff	N2-Acetyllysine
M2220.neg	-0.42	0.14	1.31	Nodiff	2'-Hydroxy-3-methoxychalcone
M2221.pos	0.45	0.39	0.85	Nodiff	Crotamiton
M2222.neg	0.01	0.43	0.89	Nodiff	4-(Difluoromethoxy)-5-methoxy-2-nitrobenzoic acid
M2223.pos	-0.18	0.73	0.39	Nodiff	5-Amino-1,4-diphenyl-1,2,3-triazole
M2224.neg	-0.62	0.69	0.37	Nodiff	5-Methyl-2-(methylsulfanyl)[1,2,4]triazolo[1,5-a]pyrimidin-7-ol
M2225.pos	0.07	0.77	0.29	Nodiff	4-Methylthioamphetamine
M2226.neg	-1.11	0.14	1.34	Nodiff	5-[(Benzylsulfanyl)methyl]-2-furoic acid
M2227.neg	0.01	0.46	0.84	Nodiff	7-(4-Fluorophenyl)-5-hydroxy-1,3-benzoxathiol-2-one
M2228.neg	-0.73	0.45	0.81	Nodiff	3-Sulfamoylbenzoic acid
M2229.pos	0.96	0.14	1.38	Nodiff	(2S)-3-(1-Benzyl-1H-imidazol-4-yl)-2-{[(tert-butoxy)carbonyl]amino}propanoic acid
M223.pos	0.87	0.37	0.87	Nodiff	4-Guanidinobutyric acid
M2230.neg	-0.78	0.46	0.70	Nodiff	Cyclopenol
M2231.pos	0.91	0.18	1.36	Nodiff	4-(1H-Indol-3-yl)-1-(morpholin-4-yl)butan-1-one
M2232.neg	-0.78	0.38	1.00	Nodiff	N-(Phenoxyacetyl)phenylalanine
M2233.neg	0.41	0.28	1.12	Nodiff	Cys-His
M2234.neg	-0.93	0.12	1.40	Nodiff	3-Dehydroshikimic acid
M2236.neg	0.50	0.37	0.98	Nodiff	2'-Hydroxy-2,4,4'-trimethoxychalcone
M2237.neg	-0.12	0.47	0.79	Nodiff	2'-Cyano[1,1'-biphenyl]-2-carboxylic acid
M2238.pos	-0.11	0.46	0.67	Nodiff	N-(2,6-Dimethylphenyl)-1-methyl-2-piperidinecarboxamide
M2239.neg	-0.01	1.00	0.02	Nodiff	Melilotoside
M224.pos	-0.11	0.87	0.16	Nodiff	Carnitine
M2240.neg	-0.08	0.88	0.08	Nodiff	2,4'-Dimethoxy-2'-hydroxychalcone
M2241.neg	0.27	0.50	0.68	Nodiff	2-Hydroxy-6-(trifluoromethyl)nicotinonitrile
M2242.pos	-0.56	0.91	0.13	Nodiff	5-Chloro-2-[(phenoxyacetyl)amino]benzoic acid
M2243.neg	-0.01	0.72	0.33	Nodiff	4-Chloro-3-{5-[(E)-(4-hydroxy-2,6-dioxo-1,6-dihydro-5(2H)-pyrimidinylidene)methyl]-2-furyl}benzoic acid
M2244.neg	0.82	0.15	1.44	Nodiff	Nordentatin
M2245.pos	-1.41	0.54	0.66	Nodiff	1,3-Benzenediol, 4-[4-(2,3-dihydro-1,4-benzodioxin-6-yl)-5-methyl-1H-pyrazol-3-yl]-6-ethyl-
M2246.pos	0.59	0.22	1.26	Nodiff	Catharanthine

M2247.neg	0.06	0.43	0.89	Nodiff	5-(2-Hydroxyphenyl)-1,3,4-oxadiazole-2(3H)-thione
M2248.pos	1.02	0.51	0.74	Nodiff	2-Chloro-5-fluorobenzoic acid
M2249.neg	-0.71	0.42	0.77	Nodiff	N-Phenyl-N'-(3-pyridinylmethyl)thiourea
M225.neg	-0.81	0.36	0.85	Nodiff	N-Acetylhistidine
M2250.neg	-0.75	0.28	1.05	Nodiff	(+)-Afzelechin
M2251.neg	2.31	0.21	1.24	Nodiff	Isomucronulatol
M2252.neg	2.79	0.20	1.22	Nodiff	Dibenzyl hydrogen phosphate
M2253.pos	-0.07	0.69	0.46	Nodiff	2-(2-Thienyl)acetamide
M2254.neg	-1.44	0.07	1.60	Nodiff	2,4-Dinitro-6-(1H-tetrazol-1-yl)phenol
M2255.pos	0.49	0.92	0.14	Nodiff	Putrescine
M2258.neg	-1.13	0.04	1.71	Nodiff	N-Acetyl-L-glutamate
M2259.pos	-0.28	0.94	0.00	Nodiff	5-Aminopentanamide
M226.neg	-0.09	0.48	0.76	Nodiff	Gly-Gly-Leu
M2262.pos	-1.16	0.16	1.41	Nodiff	Chitobiose
M2263.pos	-0.30	0.88	0.18	Nodiff	5-Guanidino-2-oxopentanoate
M2264.pos	-0.33	0.13	1.47	Nodiff	N6-Methyl-L-lysine
M2265.pos	-0.11	0.72	0.46	Nodiff	Apigenin 7-(6''-malonylglucoside)
M2267.pos	0.66	0.35	0.91	Nodiff	1-(5'-Phosphoribosyl)-5-formamido-4-imidazolecarboxamide
M2268.neg	0.79	0.35	0.98	Nodiff	S-(Phenylacetothiohydroximoyl)-L-cysteine
M2269.neg	-0.38	0.58	0.54	Nodiff	3-Oxopropanoate
M227.pos	0.46	0.39	0.91	Nodiff	Betaine aldehyde
M2270.neg	-0.01	0.98	0.01	Nodiff	(Z)-4-(2-Hydroxy-5-sulfonatophenyl)-2-oxo-3-butenoate
M2278.pos	0.32	0.61	0.45	Nodiff	gamma-Glutamyl-gamma-aminobutyraldehyde
M228.neg	0.64	0.48	0.75	Nodiff	5-Hydroxytryptophan
M2282.neg	0.37	0.40	0.88	Nodiff	(R)-S-Lactoylglutathione
M2283.pos	-0.57	0.37	0.98	Nodiff	2-(2'-Methylthio)ethylmalic acid
M2284.pos	0.53	0.21	1.27	Nodiff	4-Imidazolone-5-propanoate
M2285.pos	0.89	0.34	0.95	Nodiff	4-Guanidinobutanamide
M2286.neg	0.04	0.46	0.65	Nodiff	Coniferyl acetate
M2289.neg	0.84	0.13	1.36	Nodiff	L-Arogenate
M229.neg	1.36	0.06	1.84	Nodiff	Gly-Phe
M2290.neg	-0.59	0.58	0.54	Nodiff	Acetylcysteine
M2291.pos	1.11	0.19	1.33	Nodiff	gamma-Glutamyltyramine
M2293.pos	0.14	0.45	0.76	Nodiff	D-Glutamine
M2296.neg	-0.61	0.08	1.61	Nodiff	Dehydroascorbate
M2297.neg	0.22	0.65	0.41	Nodiff	6,8a-Seco-6,8a-deoxy-5-oxoavermectin ''1b'' aglycone
M2299.neg	-1.06	0.03	1.86	Nodiff	5'-Phosphoribosylglycinamide
M23.neg	-0.99	0.88	0.19	Nodiff	2-Ketobutyric acid
M230.pos	0.24	0.45	0.79	Nodiff	Ethambutol (dihydrochloride)
M2300.neg	-0.29	0.42	0.76	Nodiff	Tauropine
M2301.neg	-0.16	0.81	0.17	Nodiff	dAMP
M2302.pos	0.80	0.15	1.41	Nodiff	N5-Phenyl-L-glutamine
M2303.neg	-0.15	0.98	0.13	Nodiff	2-Propynal
M2304.neg	-0.15	0.63	0.37	Nodiff	S-Formylmycothiol
M2305.neg	-1.57	0.26	1.22	Nodiff	Purine deoxyribonucleoside
M2308.pos	0.19	0.43	0.76	Nodiff	3-Hydroxy-N6,N6,N6-trimethyl-L-lysine
M2309.pos	-0.70	0.36	0.83	Nodiff	Diethanolamine
M231.neg	-0.76	0.85	0.15	Nodiff	Orotic acid
M2310.neg	-0.36	0.21	1.20	Nodiff	2-Succinyl-5-enolpyruvyl-6-hydroxy-3-cyclohexene-1-carboxylate
M2311.pos	0.83	0.63	0.36	Nodiff	N-Succinyl-L-citrulline
M2315.pos	-0.62	0.31	1.04	Nodiff	D-erythro-3-Methylmalate
M2316.pos	-1.03	0.27	1.19	Nodiff	N-Formylmethionine
M2318.neg	-0.40	0.99	0.04	Nodiff	1,3,7-Trimethyluric acid
M2319.neg	0.28	0.39	0.87	Nodiff	Cellobiono-1,5-lactone
M232.pos	-1.45	0.85	0.13	Nodiff	Tyramine
M2323.neg	-1.04	0.24	1.13	Nodiff	2,3-Bis(4-hydroxyphenyl)-1,2-propanediol
M2326.neg	-1.51	0.28	1.17	Nodiff	Harmaline

M2328.neg	0.53	0.39	0.96	Nodiff	Tetrahydrobiopterin
M233.neg	1.87	0.06	1.71	Nodiff	Val-Val
M2330.pos	1.02	0.32	0.97	Nodiff	Glutathionylspermine
M2331.neg	0.88	0.11	1.58	Nodiff	N-(L-Arginino)succinate
M2332.pos	0.36	0.51	0.74	Nodiff	3-Ketolactose
M2333.neg	-0.49	0.25	1.04	Nodiff	Cephalosporin C
M2334.neg	0.81	0.27	1.12	Nodiff	S-(Hydroxyphenylacetothiohydroximoyl)-L-cysteine
M2336.pos	0.34	0.42	0.86	Nodiff	N-Acetyl-D-glucosaminate
M2339.pos	-0.35	0.98	0.05	Nodiff	L-Asparagine
M234.neg	0.24	0.56	0.72	Nodiff	Thr-Phe
M2341.neg	1.02	0.70	0.52	Nodiff	N2-Succinyl-L-arginine
M2344.neg	-0.19	0.57	0.63	Nodiff	Mycothiol
M2345.neg	0.16	0.99	0.07	Nodiff	Pyridoxal phosphate
M2346.pos	-0.73	0.53	0.64	Nodiff	Pantothenol
M2347.neg	-0.22	0.45	0.76	Nodiff	(7R)-7-(5-Carboxy-5-oxopentanoyl)aminoce phalosporinate
M2349.neg	-0.78	0.81	0.20	Nodiff	N-Acetylbialaphos
M235.neg	0.86	0.20	1.31	Nodiff	N-Acetylvaline
M2350.neg	-0.19	0.67	0.38	Nodiff	6-Hydroxykynurenate
M2351.neg	-0.12	0.52	0.78	Nodiff	D-Allose
M2353.neg	0.42	0.39	0.95	Nodiff	Glycerol
M2354.neg	-0.64	0.44	0.76	Nodiff	6-Acetyl-D-glucose
M2357.pos	-0.38	0.29	0.97	Nodiff	N-Formyl-L-aspartate
M236.pos	0.93	0.26	1.11	Nodiff	LPC(18:2/0:0)
M2360.pos	0.57	0.23	1.20	Nodiff	gamma-Glutamyl-beta-cyanoalanine
M2361.neg	0.45	0.39	0.86	Nodiff	1,3,7-Trimethyl-5-hydroxyisourate
M2362.pos	1.38	0.02	1.85	Nodiff	Iminoglycine
M2363.neg	0.18	0.49	0.78	Nodiff	L-2-Amino-6-oxoheptanedioate
M2364.neg	-0.57	0.03	1.90	Nodiff	Uracil 5-carbaldehyde
M2366.pos	0.00	0.97	0.07	Nodiff	(E)-4-(Trimethylammonio)but-2-enoate
M237.neg	0.60	0.36	0.94	Nodiff	N-Lactoylphenylalanine
M2370.neg	0.03	0.80	0.25	Nodiff	Oxaloglutarate
M2371.neg	-1.03	0.04	1.68	Nodiff	cis-(Homo)2-aconitate
M2372.neg	-0.32	0.40	0.73	Nodiff	Urocanate
M2373.neg	1.26	0.01	1.95	Nodiff	O-Carbamoyl-L-serine
M2374.neg	0.00	0.83	0.23	Nodiff	N-Methylhydantoin
M2375.pos	-0.59	0.65	0.49	Nodiff	9-OxoODE
M2376.pos	1.03	0.18	1.35	Nodiff	Cellohexaose
M2377.neg	0.54	0.37	0.82	Nodiff	L-2-Aminoadipate adenylate
M2378.pos	-1.52	0.30	1.11	Nodiff	2-Hydroxy-6-oxo-6-(2-hydroxyphenyl)-hex a-2,4-dienoate
M2379.neg	-0.59	0.16	1.33	Nodiff	2',3'-Cyclic UMP
M238.pos	-0.39	0.43	0.88	Nodiff	Adenine
M2380.pos	-0.44	0.15	1.44	Nodiff	N-Acetyl-beta-D-glucosaminylamine
M2381.neg	2.46	0.08	1.71	Nodiff	1-O-[2-(L-Cysteinamido)-2-deoxy-alpha-D-glucopyranosyl]-1D-myo-inositol
M239.pos	-0.38	0.23	1.21	Nodiff	Daltogen
M24.neg	-0.34	0.40	0.81	Nodiff	3alpha-Hydroxy-12-oxo-5beta-cholan-24-oi c acid
M240.pos	1.13	0.24	1.06	Nodiff	Sphingosine
M241.neg	0.26	0.97	0.09	Nodiff	alpha-Ketoisovaleric acid
M242.pos	0.25	0.69	0.32	Nodiff	Tetraethylene glycol
M243.neg	0.48	0.96	0.11	Nodiff	2-Ketocaproic acid
M244.neg	0.48	0.96	0.11	Nodiff	3-Methyl-2-oxovaleric acid
M245.neg	0.48	0.96	0.11	Nodiff	Ketoleucine
M246.pos	-0.70	0.37	0.80	Nodiff	NAE(18:3(9Z,12Z,15Z))
M247.neg	-0.05	0.99	0.02	Nodiff	Emodin
M248.pos	0.49	0.88	0.16	Nodiff	4-hydroxy-1H-indole-3-carbaldehyde
M249.neg	0.17	0.46	0.72	Nodiff	Deoxycholic acid
M25.pos	-0.29	0.87	0.18	Nodiff	2'-O-methylcytidine
M250.neg	-0.41	0.24	1.10	Nodiff	Glucitol
M251.neg	0.51	0.09	1.59	Nodiff	cis-鈥?,鈥?0-鈥婂 poxystearic acid
M252.neg	0.00	0.80	0.29	Nodiff	trans-Vaccenic acid

M253.pos	0.15	0.58	0.61	Nodiff	Isoleucine
M254.neg	-0.03	0.98	0.09	Nodiff	2-Hydroxystearic acid
M255.neg	0.05	0.49	0.73	Nodiff	4-Hydroxycinnamic acid
M256.pos	0.19	0.58	0.58	Nodiff	Isobutyrylcarnitine (Car(4:0))
M257.pos	0.36	0.51	0.68	Nodiff	N-acetyldopamine
M258.pos	-0.39	0.43	0.88	Nodiff	2-Aminopurine
M259.pos	-0.70	0.37	0.80	Nodiff	NAE(18:3(6Z,9Z,12Z))
M26.neg	0.07	0.86	0.16	Nodiff	Phosphate
M260.pos	0.20	0.44	0.76	Nodiff	Stachyose
M261.pos	-1.86	0.89	0.05	Nodiff	Cystine
M262.neg	-0.24	0.30	0.98	Nodiff	Citric acid
M263.pos	-0.13	0.80	0.21	Nodiff	Glutamate
M264.pos	-0.25	0.40	0.90	Nodiff	Maltose
M265.neg	-0.46	0.12	1.39	Nodiff	Glucuronic acid
M266.neg	-0.91	0.07	1.60	Nodiff	gamma-Glutamylvaline
M267.pos	-0.25	0.93	0.11	Nodiff	Glucosamine
M268.neg	1.16	0.10	1.63	Nodiff	Glycylleucine
M269.neg	-0.18	0.85	0.21	Nodiff	Pseudouridine
M27.neg	0.54	0.22	1.29	Nodiff	Glutamine
M270.pos	0.20	0.59	0.51	Nodiff	Pro-Phe
M271.pos	-0.04	0.79	0.21	Nodiff	Isonicotinic acid
M272.pos	-0.08	0.96	0.07	Nodiff	Riboflavin
M273.neg	0.94	0.20	1.30	Nodiff	Val-Met
M274.pos	0.74	0.29	1.00	Nodiff	LPC(20:0)
M275.pos	2.68	0.16	1.33	Nodiff	1-Methylguanosine
M276.pos	2.68	0.16	1.33	Nodiff	2'-O-Methylguanosine
M277.pos	0.76	0.13	1.48	Nodiff	Palmitoyl sphingomyelin
M278.neg	0.03	0.48	0.72	Nodiff	4-Hydroxybenzoic acid
M279.neg	0.26	0.40	0.83	Nodiff	Allantoin
M28.neg	-0.24	0.95	0.14	Nodiff	Pro-Gly
M280.neg	0.97	0.08	1.54	Nodiff	Apocholic acid
M281.neg	-0.15	0.64	0.51	Nodiff	Ferulate
M282.pos	0.72	0.09	1.57	Nodiff	Betaxolol
M283.neg	-0.76	0.47	0.79	Nodiff	Allolithocholic acid
M284.neg	0.37	0.28	0.98	Nodiff	Heptadecanoic acid
M285.neg	0.11	0.66	0.52	Nodiff	Sarcosine
M286.neg	0.17	0.46	0.72	Nodiff	Hyodeoxycholic acid
M287.neg	-0.41	0.24	1.10	Nodiff	Iditol
M288.neg	1.11	0.02	1.89	Nodiff	16-Hydroxypalmitic acid
M289.neg	1.16	0.03	1.90	Nodiff	Raffinose
M29.neg	0.11	0.66	0.52	Nodiff	Alanine
M290.pos	-0.54	0.50	0.72	Nodiff	Prolylhydroxyproline
M291.neg	-0.35	0.21	1.42	Nodiff	Turanose
M292.pos	0.14	0.71	0.47	Nodiff	N5-acetyl-l-ornithine
M293.pos	-1.01	0.43	0.82	Nodiff	Histidinol
M294.pos	-0.42	0.49	0.73	Nodiff	N-Acetylputrescine
M295.pos	-0.04	0.83	0.30	Nodiff	Acetylcarnitine (Car(2:0))
M296.pos	0.53	0.48	0.70	Nodiff	His-Phe
M297.neg	0.14	0.52	0.67	Nodiff	Xanthine
M299.pos	-0.28	0.55	0.61	Nodiff	5'-Deoxyadenosine
M3.neg	-0.27	0.65	0.45	Nodiff	N-Acetylneuraminic acid
M30.neg	-0.36	0.53	0.61	Nodiff	5-Oxo-L-prolyl-L-proline
M300.neg	-0.22	0.48	0.76	Nodiff	Bisindolylmaleimide VIII (acetate)
M301.pos	-0.46	0.47	0.77	Nodiff	Pyridoxine
M302.neg	1.92	0.30	1.06	Nodiff	Monomethyl phthalate
M303.neg	1.78	0.18	1.31	Nodiff	3-Hydroxydecanoic acid
M304.neg	-0.37	0.09	1.45	Nodiff	3-Hydroxypyruvic acid
M305.neg	0.60	0.36	0.94	Nodiff	N-Lactoyl-Phenylalanine
M306.pos	-0.82	0.03	1.82	Nodiff	Gly-Asp
M307.pos	0.13	0.60	0.50	Nodiff	Arg-Ala
M308.pos	0.52	0.30	1.04	Nodiff	Sorbose
M309.pos	0.43	0.41	0.79	Nodiff	Propionylcarnitine (Car(3:0))
M31.pos	-0.25	0.58	0.57	Nodiff	Nortropine
M310.pos	0.25	0.77	0.34	Nodiff	5-Methyl DL-glutamate

M311.neg	-0.65	0.17	1.33	Nodiff	2,4-Dihydroxybutanoic acid
M312.neg	0.78	0.36	0.94	Nodiff	N-Acetylalanine
M313.neg	-0.42	0.54	0.70	Nodiff	Homovanillic acid sulfate
M314.neg	-0.54	0.36	0.88	Nodiff	Glycolithocholic acid 3-sulfate
M315.pos	-0.02	0.93	0.10	Nodiff	5-Methylcytosine
M316.neg	-0.07	0.91	0.13	Nodiff	Vanillic acid
M317.neg	-1.00	0.55	0.67	Nodiff	Threitol
M318.neg	-1.10	0.17	1.36	Nodiff	Zoxazolamine
M319.neg	-0.22	0.72	0.29	Nodiff	9-Oxooctadecanoic acid
M32.neg	-0.59	0.05	1.78	Nodiff	Cholic acid 7-sulfate
M320.neg	0.21	0.50	0.62	Nodiff	omega-Muricholic acid
M321.pos	0.35	0.48	0.77	Nodiff	3-Pyridylacetic acid
M322.neg	0.05	0.49	0.73	Nodiff	2-Hydroxycinnamic acid
M323.pos	-0.25	0.40	0.90	Nodiff	Melibiose
M324.neg	-0.76	0.47	0.79	Nodiff	Isolithocholic acid
M325.pos	-0.28	0.55	0.61	Nodiff	Deoxyadenosine
M326.pos	-0.28	0.55	0.61	Nodiff	Cordycepin
M327.pos	-0.55	0.38	0.79	Nodiff	Phosphorylcholine
M328.pos	0.27	0.23	1.18	Nodiff	Histamine
M329.pos	-0.66	0.06	1.62	Nodiff	Thiamine
M33.pos	0.53	0.43	0.77	Nodiff	PC(P-16:0/0:0)
M330.pos	0.55	0.88	0.15	Nodiff	4-(dimethylamino)butanoate
M331.neg	1.02	0.61	0.66	Nodiff	Glycocholic acid
M332.pos	0.23	0.60	0.52	Nodiff	N-Acetylgalactosamine
M333.neg	0.63	0.35	0.97	Nodiff	Acetylleucine
M334.neg	-0.53	0.39	0.78	Nodiff	Formylmethionine
M335.pos	-0.55	0.02	1.86	Nodiff	Pyridoxal (Vitamin B6)
M336.neg	1.28	0.31	1.04	Nodiff	13(S)-HpOTrE
M337.neg	1.10	0.33	0.99	Nodiff	lactitol
M338.neg	0.16	0.49	0.76	Nodiff	Ser-Ala
M339.neg	-0.96	0.04	1.69	Nodiff	O-Acetylserine
M34.neg	0.86	0.16	1.44	Nodiff	Leu-Leu
M340.neg	-0.61	0.37	0.85	Nodiff	Mannose
M341.pos	-0.41	0.13	1.50	Nodiff	2-Aminoheptanoic acid
M342.pos	1.34	0.17	1.26	Nodiff	Car(18:1)
M343.pos	0.18	0.66	0.43	Nodiff	1-Methylguanine
M344.pos	-0.25	0.98	0.04	Nodiff	Alfacalcidol
M345.neg	-0.29	0.41	0.97	Nodiff	11-Dehydrocorticosterone
M346.neg	-0.29	0.41	0.97	Nodiff	Docebenone
M347.neg	0.03	0.48	0.72	Nodiff	3-Hydroxybenzoic acid
M348.neg	-0.76	0.47	0.79	Nodiff	Lithocholic acid
M349.neg	1.02	0.61	0.66	Nodiff	Glyco-gamma-muricholic acid
M35.neg	0.45	0.22	1.27	Nodiff	7-Methylguanine
M350.pos	-0.15	0.84	0.22	Nodiff	N,N-Dimethylarginine (ADMA)
M351.pos	0.09	0.62	0.53	Nodiff	3-Phosphoserine
M352.pos	0.67	0.37	0.88	Nodiff	3-Methylhistidine
M353.neg	0.96	0.30	1.05	Nodiff	(2R)-6-Oxo-2-piperidinecarboxylic acid
M354.pos	0.01	0.78	0.29	Nodiff	Guanine
M355.neg	1.35	0.11	1.51	Nodiff	LPE(18:1(9Z)/0:0)
M356.neg	-0.64	0.27	1.09	Nodiff	Hydroxyphenyllactic acid
M357.neg	1.25	0.40	0.92	Nodiff	Taurodeoxycholic acid
M358.neg	0.01	0.46	0.68	Nodiff	Taurodehydrocholic acid
M359.neg	-0.02	0.46	0.76	Nodiff	4-Ethoxybenzoic acid
M36.neg	-0.67	0.48	0.73	Nodiff	2-Hydroxy-3-methylbutyric acid
M360.pos	0.34	0.92	0.18	Nodiff	2'-Aminoacetophenone
M361.neg	0.12	0.66	0.42	Nodiff	Embelin
M362.pos	-0.61	0.20	1.38	Nodiff	Daminozide
M363.pos	0.23	0.60	0.52	Nodiff	N-Acetylmannosamine
M364.neg	1.26	0.09	1.57	Nodiff	LPC(13:0)
M365.pos	-0.08	0.95	0.13	Nodiff	Apiin
M366.neg	-1.77	0.27	1.04	Nodiff	LPG(18:1(9Z))
M367.neg	-1.78	0.16	1.39	Nodiff	Sulfachloropyridazine
M368.pos	0.87	0.56	0.69	Nodiff	Glycodeoxycholic acid
M369.neg	-1.36	0.06	1.83	Nodiff	5-Hydroxyindole-3-acetic acid

M37.neg	0.99	0.67	0.51	Nodiff	Taurochenodeoxycholic acid
M370.pos	0.52	0.30	1.04	Nodiff	Fructose
M371.pos	-0.39	0.23	1.19	Nodiff	N6,N6,N6-Trimethyllysine
M372.neg	0.59	0.37	1.00	Nodiff	Fructo-鈥婂 ligosaccharide DP7/GF6
M373.neg	-1.13	0.10	1.59	Nodiff	Histidine
M374.neg	1.92	0.16	1.42	Nodiff	Gly-His
M375.pos	-0.18	0.49	0.70	Nodiff	7,12-Dioxolithocholic acid
M376.pos	-0.66	0.38	0.80	Nodiff	4',5-Dihydroxyflavone
M377.pos	-0.40	0.91	0.17	Nodiff	5-Acetyluracil
M378.neg	-1.41	0.06	1.82	Nodiff	Dimethylglycine
M379.neg	0.36	0.44	0.74	Nodiff	Glucono-1,5-lactone
M38.neg	2.51	0.21	1.26	Nodiff	Thymidine
M380.pos	-0.55	0.14	1.37	Nodiff	Betazole
M381.pos	1.21	0.20	1.15	Nodiff	Palmitoylcarnitine (Car(16:0))
M382.pos	0.87	0.56	0.69	Nodiff	Glycochenodeoxycholic acid
M383.pos	0.58	0.20	1.27	Nodiff	Cellobiose
M384.neg	-0.24	0.30	0.98	Nodiff	Isocitric acid
M385.neg	0.80	0.06	1.69	Nodiff	gamma-Glutamylglutamic acid
M386.pos	0.98	0.13	1.51	Nodiff	H-Val-Ala-OH
M387.pos	-0.60	0.45	0.75	Nodiff	Wogonoside
M388.neg	1.78	0.18	1.31	Nodiff	10-Hydroxydecanoic acid
M389.neg	1.02	0.61	0.66	Nodiff	Glyco-beta-muricholic acid
M39.pos	0.49	0.88	0.16	Nodiff	1,5-Isoquinolinediol
M390.pos	0.12	0.45	0.81	Nodiff	2-Aminoadipic acid
M391.pos	1.35	0.03	1.96	Nodiff	Met-Gly
M392.neg	-1.02	0.33	0.93	Nodiff	7-Ketodeoxycholic acid
M393.pos	-0.09	0.54	0.63	Nodiff	Allopurinol-1-ribonucleoside
M394.pos	0.84	0.24	1.13	Nodiff	PC(16:0/16:0)
M395.neg	-0.21	0.84	0.14	Nodiff	Chenodeoxycholic acid
M396.pos	1.22	0.05	1.67	Nodiff	Sph(d18:0)
M397.neg	1.25	0.83	0.19	Nodiff	Xipamide
M398.pos	0.34	0.92	0.18	Nodiff	N-Phenylacetamide
M399.neg	0.15	0.60	0.54	Nodiff	O-Phosphoethanolamine
M4.neg	0.94	0.33	1.00	Nodiff	Glycine
M40.pos	0.49	0.88	0.16	Nodiff	Jineol
M400.pos	-1.83	0.59	0.60	Nodiff	Arg-Phe
M401.pos	0.34	0.42	0.86	Nodiff	Tyrosylalanine
M402.pos	1.48	0.29	1.05	Nodiff	Sph(t18:0)
M403.pos	0.73	0.14	1.41	Nodiff	Apigenin
M404.neg	0.08	0.97	0.05	Nodiff	Quercetin
M406.neg	0.40	0.43	0.77	Nodiff	3,6-Dioxo-5alpha-cholan-24-oic acid
M407.neg	-0.24	0.95	0.14	Nodiff	Glycylproline
M408.neg	0.86	0.20	1.31	Nodiff	2-Methylbutyrylglycine
M409.neg	-0.02	0.46	0.76	Nodiff	Ethyl 4-hydroxybenzoate
M41.neg	-0.16	0.99	0.07	Nodiff	cis-11.14-Eicosadienoic acid
M410.neg	-0.02	0.46	0.76	Nodiff	Ethyl 3-hydroxybenzoate
M411.neg	-1.37	0.18	1.33	Nodiff	Cauloside A
M412.pos	0.22	0.49	0.65	Nodiff	Lauramine oxide
M413.pos	0.21	0.54	0.57	Nodiff	7-Ketocholesterol
M414.neg	-0.02	0.46	0.76	Nodiff	2-Phenyllactic acid
M415.pos	0.12	0.45	0.81	Nodiff	N-Methylglutamic acid
M416.pos	0.21	0.54	0.57	Nodiff	25-Hydroxyvitamin D3
M417.neg	-0.40	0.16	1.30	Nodiff	Biopterin
M418.pos	0.53	0.27	1.07	Nodiff	LPC(18:0/0:0)
M419.neg	-1.17	0.37	0.85	Nodiff	4-Hydroxyphenylglycolic acid
M42.neg	0.20	0.63	0.46	Nodiff	cis-11-Eicosenoic acid
M420.neg	0.14	0.52	0.67	Nodiff	Oxypurinol
M421.neg	0.48	0.88	0.22	Nodiff	5-hydroxymethyl-2'-deoxycytidine
M422.pos	0.68	0.31	1.00	Nodiff	Linoleamide
M423.neg	-0.36	0.44	0.93	Nodiff	Phenylpyruvic acid
M424.neg	0.70	0.19	1.37	Nodiff	Phloroglucinaldehyde
M425.neg	0.27	0.41	0.85	Nodiff	Allopurinol
M426.pos	2.68	0.16	1.33	Nodiff	2-Methylguanosine
M427.neg	-1.02	0.33	0.93	Nodiff	3-Oxocholic acid

M428.pos	0.84	0.24	1.13	Nodiff	PC(18:0/14:0)
M429.pos	0.63	0.12	1.41	Nodiff	S-Adenosylmethionine
M43.pos	0.24	0.43	0.71	Nodiff	Tris(2,4-di-tert-butylphenyl)phosphate
M430.neg	0.47	0.38	0.98	Nodiff	Propionic acid
M431.neg	0.26	0.26	1.16	Nodiff	Octadecanedioic acid
M432.pos	0.52	0.30	1.04	Nodiff	Tagatose
M433.neg	0.00	0.47	0.73	Nodiff	Glucosamine 1-phosphate
M434.pos	-0.46	0.73	0.41	Nodiff	PC(16:0/20:4)
M435.neg	-0.01	0.97	0.02	Nodiff	Saccharin
M436.neg	0.78	0.36	0.94	Nodiff	4-Hydroxyproline
M437.pos	0.01	0.78	0.29	Nodiff	2-Hydroxyadenine
M438.neg	-0.02	0.46	0.76	Nodiff	Desaminotyrosine
M439.neg	0.70	0.19	1.37	Nodiff	2,6-Dihydroxybenzoic acid
M44.neg	0.12	0.66	0.42	Nodiff	Tetradecyl sulfate (sodium)
M440.neg	0.70	0.19	1.37	Nodiff	2,3-Dihydroxybenzoic acid (Pyrocatechuic acid)
M441.neg	0.14	0.43	0.83	Nodiff	Gluconic acid
M442.pos	-0.24	0.40	0.81	Nodiff	4-(4'-Methyl-[2,2'-bipyridin]-4-yl)butanoic acid
M443.pos	-0.26	0.77	0.25	Nodiff	3-Hydroxy-4-aminopyridine
M444.pos	-0.01	0.98	0.01	Nodiff	gamma-Linolenic acid
M445.pos	-0.53	0.39	0.95	Nodiff	PC(18:1(9Z)/18:1(9Z))
M446.pos	-0.53	0.39	0.95	Nodiff	PC(18:1(6Z)/18:1(6Z))
M447.pos	-0.53	0.39	0.95	Nodiff	PC(18:1(9E)/18:1(9E))
M448.neg	-0.15	0.64	0.51	Nodiff	Isoferulic acid
M449.neg	0.77	0.36	0.95	Nodiff	5-Aminopentanoic acid
M45.neg	0.86	0.16	1.44	Nodiff	Ile-Leu
M450.pos	0.75	0.36	0.98	Nodiff	Cytidine
M451.neg	0.11	0.66	0.51	Nodiff	Prunin
M452.neg	-0.01	0.79	0.29	Nodiff	Bendamustine
M453.pos	0.35	0.38	0.83	Nodiff	DHA ethyl ester
M454.pos	0.22	0.40	0.82	Nodiff	3-Methyl-1H-pyrazole-4-carbaldehyde
M455.pos	0.73	0.42	0.77	Nodiff	Isoquinoline
M456.pos	-0.22	0.40	0.94	Nodiff	PE(18:0/18:2)
M457.neg	1.53	0.02	1.96	Nodiff	Galactinol
M458.neg	0.11	0.66	0.51	Nodiff	Choerospondin
M459.pos	0.09	0.42	0.77	Nodiff	1-Methylhistidine
M46.neg	-0.67	0.48	0.73	Nodiff	2-Hydroxy-2-methylbutyric acid
M460.neg	-0.11	0.65	0.48	Nodiff	Homocarnosine
M461.pos	0.27	0.64	0.52	Nodiff	Choline
M462.neg	0.93	0.07	1.65	Nodiff	2-Hydroxyhexanedioic acid
M463.pos	0.26	0.48	0.60	Nodiff	Guvacoline (hydrochloride)
M464.pos	0.44	0.32	0.95	Nodiff	Isatin
M465.pos	0.40	0.46	0.69	Nodiff	HQNO
M466.pos	-0.07	0.83	0.20	Nodiff	Indoleacetic acid
M467.pos	0.17	0.38	0.87	Nodiff	Normetanephrine
M468.pos	0.40	0.50	0.76	Nodiff	gamma-Glutamylleucine
M469.pos	0.53	0.16	1.43	Nodiff	3-Methyladenine
M47.pos	0.49	0.88	0.16	Nodiff	8-Hydroxycarbostyril
M470.neg	-1.14	0.28	1.21	Nodiff	Salicyluric acid
M471.neg	0.26	0.42	0.87	Nodiff	Monomethyl fumarate
M472.neg	0.26	0.43	0.75	Nodiff	2-(2-Butoxyethoxy)acetic acid
M473.neg	-0.61	0.23	1.19	Nodiff	gamma-Glutamyllysine
M474.pos	0.87	0.19	1.26	Nodiff	Paracetamol
M475.neg	0.25	0.78	0.28	Nodiff	Bifonazole
M476.neg	1.16	0.10	1.63	Nodiff	N6-Acetyllysine
M477.pos	-0.38	0.30	0.95	Nodiff	N2-Acetylornithine
M478.neg	0.48	0.96	0.11	Nodiff	4-Oxohexanoic acid
M479.neg	0.79	0.11	1.58	Nodiff	Emodin-8-glucoside
M48.pos	0.49	0.88	0.16	Nodiff	5-Phenylisoxazol-3-ol
M480.pos	1.76	0.20	1.26	Nodiff	2,2,6,6-Tetramethyl-4-piperidinyl 2-methylacrylate
M481.neg	1.31	0.23	1.24	Nodiff	3-Hydroxymandelic acid
M482.neg	0.10	0.42	0.83	Nodiff	Ganoderic acid A

M483.neg	0.10	0.42	0.83	Nodiff	Ganoderic acid B
M484.pos	-0.87	0.02	1.93	Nodiff	Miglitol
M485.pos	0.89	0.06	1.65	Nodiff	o-Tyrosine
M486.pos	0.89	0.22	1.21	Nodiff	Sultopride hydrochloride
M487.pos	-0.59	0.95	0.08	Nodiff	Nikethamide
M488.neg	0.70	0.19	1.37	Nodiff	Gentisic acid
M489.neg	-0.37	0.15	1.34	Nodiff	2-Oxoadipic acid
M49.neg	1.53	0.02	1.96	Nodiff	Trehalose
M490.pos	1.74	0.11	1.46	Nodiff	S-Methylmethionine
M491.pos	0.44	0.40	0.85	Nodiff	5-Hydroxymethylcytidine
M492.neg	2.51	0.21	1.26	Nodiff	Lapachol
M493.neg	-0.16	0.65	0.47	Nodiff	Aurantiamide acetate
M494.pos	0.87	0.21	1.09	Nodiff	2,6-Dimethoxybenzoic acid
M495.pos	0.01	0.96	0.01	Nodiff	AG 1295
M496.pos	0.16	0.43	0.84	Nodiff	Caffeic acid
M497.neg	-0.49	0.16	1.31	Nodiff	2-Furoylglycine
M498.pos	1.94	0.15	1.48	Nodiff	Benazepril
M499.neg	0.57	0.09	1.51	Nodiff	8-Oxoadenine
M5.neg	-1.41	0.06	1.82	Nodiff	2-Aminoisobutyric acid
M50.neg	-0.37	0.09	1.45	Nodiff	Malonic acid
M500.neg	0.57	0.19	1.35	Nodiff	Apigenin-7-O-glucoside
M501.neg	-0.49	0.97	0.03	Nodiff	2,3-Dinor-11b-PGF2a
M502.neg	0.01	0.98	0.01	Nodiff	9-Hydroxyrisperidone
M503.neg	0.72	0.48	0.64	Nodiff	Lys-Pro
M504.neg	1.67	0.17	1.39	Nodiff	b-Ala-Lys
M505.pos	0.96	0.13	1.46	Nodiff	ellipticine
M506.neg	-0.14	0.60	0.41	Nodiff	D-Ribose
M507.neg	-1.78	0.93	0.05	Nodiff	Sunitinib
M508.neg	0.15	0.44	0.82	Nodiff	Tyr-Lys
M509.neg	0.67	0.60	0.68	Nodiff	3-(2-Chlorophenyl)-1H-pyrazol-5-amine
M51.neg	0.01	0.47	0.87	Nodiff	Cysteic acid
M510.neg	0.08	0.44	0.86	Nodiff	Kaempferol-7-O-glucoside
M511.neg	-0.77	0.33	0.92	Nodiff	5-Chloro-4(3H)-quinazolinone
M512.neg	1.15	0.29	1.10	Nodiff	Dimethylmalonic_acid
M513.pos	0.33	0.40	0.81	Nodiff	Nandrolone
M514.neg	1.22	0.31	1.05	Nodiff	8-Oxononanoic acid
M515.neg	-0.06	0.66	0.37	Nodiff	3-Chloro-6-nitro-9H-carbazole
M516.neg	0.34	0.36	0.94	Nodiff	alpha-Keto-gamma-(methylthio)butyric_acid
M517.neg	-0.61	0.58	0.57	Nodiff	5-(3-Chlorophenyl)-5-methylimidazolidine-2,4-dione
M519.pos	0.46	0.49	0.66	Nodiff	LPC(18:1)
M52.neg	-0.62	0.16	1.25	Nodiff	Suberic acid
M520.pos	-0.75	0.02	1.86	Nodiff	1H-Indole-1-acetic acid, 5-fluoro-2-methyl-3-(2-quinolinylmethyl)-
M521.neg	3.11	0.08	1.69	Nodiff	Methanone, [1-(5-fluoropentyl)-2-hydroxy-1H-indol-3-yl]-1-naphthalenyl-
M522.neg	0.11	0.59	0.59	Nodiff	N-Nitroso-N-methyl-3-aminopropionic acid
M523.pos	0.54	0.22	1.21	Nodiff	5,7-dimethoxy-2-phenyl-4H-chromen-4-one
M524.neg	-0.49	0.08	1.51	Nodiff	4-(Hexopyranosyloxy)-3-methoxybenzoic acid
M525.neg	-0.12	0.59	0.61	Nodiff	Soyasaponin II
M526.pos	0.65	0.18	1.33	Nodiff	.alpha.-Pyrrolidinobutiothiophenone
M527.neg	-0.05	0.86	0.25	Nodiff	Hederoside F
M528.neg	0.20	0.42	0.90	Nodiff	Echinocystic acid 3-glucoside
M529.neg	-0.32	0.42	0.71	Nodiff	10-Hydroxy-2,4b,8-trimethyl-2-(2-((3,4,5-trihydroxy-6-(hydroxymethyl)tetrahydro-2H-pyran-2-yl)oxy)ethyl)dodecahydro-4a,10a-epoxyphenanthrene-8-carboxylic acid
M53.neg	1.20	0.30	1.08	Nodiff	Uric acid
M531.neg	0.37	0.41	0.89	Nodiff	(Z)-6-Octadecenoic acid

M532.pos	0.23	0.42	0.86	Nodiff	4-Chloro-5,6,7,8-tetrahydroquinazolin-2-amine
M533.pos	0.71	0.58	0.45	Nodiff	Glycerophospho-N-palmitoylethanolamine
M534.neg	-0.50	0.24	1.18	Nodiff	(2E)-2-(Propan-2-yl)but-2-enedioic acid
M535.pos	-0.46	0.13	1.39	Nodiff	1H-Pyrazolo[3,4-d]pyrimidine-3,4-diamine, N3-(4-fluorophenyl)-
M536.pos	-0.42	0.45	0.78	Nodiff	1-Palmitoyl-2-linoleoyl-sn-glycero-3-phosphocholine
M537.neg	-0.27	0.96	0.02	Nodiff	3-Hydroxy-2-methylbenzoic acid
M538.neg	-0.17	0.79	0.24	Nodiff	4,6-Dihydroxypyrimidine
M539.neg	0.29	0.38	0.90	Nodiff	Succinic_anhydride
M54.pos	-0.50	0.54	0.66	Nodiff	Trimethylamine N-oxide
M540.neg	-0.57	0.21	1.20	Nodiff	Phomalone
M541.neg	-0.17	0.84	0.22	Nodiff	8,11-Eicosadiynoic acid
M542.neg	-0.08	0.48	0.57	Nodiff	Trifluoroacetic acid
M543.neg	-0.22	0.55	0.70	Nodiff	Triglochinic acid
M544.neg	0.43	0.37	0.94	Nodiff	Maltobionate
M545.pos	-0.14	0.96	0.06	Nodiff	N-Acetylglucosaminylasparagine
M546.neg	-0.37	0.62	0.47	Nodiff	3-Aminobutanoic acid
M547.pos	0.25	0.71	0.35	Nodiff	2-(3,5-Difluorophenyl)benzonitrile
M548.neg	2.05	0.17	1.34	Nodiff	Ile-Lys
M549.neg	-0.24	0.54	0.55	Nodiff	D-SEDOHEPTULOSE
M55.neg	-1.10	0.22	1.15	Nodiff	N-Acetylglutamine
M550.neg	0.07	0.87	0.29	Nodiff	Pro-Thr
M551.pos	0.95	0.27	1.04	Nodiff	His-Ile
M552.neg	1.65	0.02	2.05	Nodiff	Thr-Thr
M553.pos	1.43	0.03	1.79	Nodiff	Ile-Asn
M554.pos	0.50	0.24	1.17	Nodiff	5-(2-Furyl)-4H-1,2,4-triazol-3-amine
M555.neg	-0.06	0.92	0.11	Nodiff	Cylindrospermopsin
M556.pos	-0.07	0.81	0.15	Nodiff	7H-[1,2,4]Triazolo[4,3-b][1,2,4]triazole-3,7-diamine
M557.neg	0.37	0.97	0.05	Nodiff	3-Heptanone, 1,7-bis(3,4-dihydroxyphenyl)-6-methoxy-
M558.neg	1.22	0.23	1.08	Nodiff	Pro-Leu
M559.pos	0.41	0.37	0.91	Nodiff	Atrazine desethyl
M56.neg	0.29	0.41	0.86	Nodiff	Threonic acid
M560.neg	-0.70	0.58	0.58	Nodiff	3,4-Dihydro-3-oxo-2H-(1,4)-benzoxazin-2-ylacetic acid
M561.neg	-0.42	0.48	0.73	Nodiff	DL-2-Methylglutamic acid
M562.neg	0.37	0.31	1.05	Nodiff	1-(4-Hydroxyphenyl)-2-methylaminoethanone
M563.pos	0.36	0.40	0.83	Nodiff	Cinncassiol_E
M564.neg	0.46	0.35	0.96	Nodiff	2-Amino-3-[4-(carboxymethyl)phenyl]propanoic acid
M565.neg	-3.68	0.08	1.66	Nodiff	(.alpha.S,1S,2R)-.alpha.-Amino-2-carboxycyclopropaneacetic acid
M566.neg	-0.12	0.51	0.54	Nodiff	CROTONIC ACID
M567.neg	0.49	0.38	0.91	Nodiff	Succinic_acid_semialdehyde
M568.pos	1.32	0.28	1.07	Nodiff	HEPES
M569.neg	-0.56	0.19	1.23	Nodiff	N-Methyl-N-(methylsulfonyl)glycine
M57.neg	0.74	0.16	1.26	Nodiff	Gly-Val
M570.neg	-0.19	0.62	0.44	Nodiff	3,4-Diamino-1-benzenesulfonic acid
M571.neg	-0.14	0.98	0.10	Nodiff	(12Z)-9,10,11-Trihydroxyoctadec-12-enoic acid
M573.neg	1.23	0.38	0.89	Nodiff	Methyl 2-hydroxy-4-methoxybenzoate
M574.neg	-0.75	0.27	1.04	Nodiff	3-(3-Chlorophenyl)-2,4-imidazolidinedione
M575.neg	0.44	0.95	0.13	Nodiff	13-OxoODE
M576.neg	0.69	0.09	1.62	Nodiff	Pinolenic acid
M577.neg	-0.71	0.34	0.92	Nodiff	7-Bromo-1H-pyrrolo[3,2-d]pyrimidin-4(5H)-one
M578.neg	-0.27	0.36	0.83	Nodiff	Lactaldehyde
M579.neg	-0.55	0.27	1.02	Nodiff	4-Hydroxyglutamic acid
M58.neg	0.62	0.00	2.13	Nodiff	Cysteine S-sulfate
M580.pos	0.69	0.21	1.17	Nodiff	LPE(16:0)

M581.neg	0.05	0.80	0.20	Nodiff	4-Hydroxy-3',6-dimethoxy-5'-methylspiro[1-benzofuran-2,4'-cyclohex-2-ene]-1',3-dione
M582.pos	0.02	0.70	0.41	Nodiff	1-Phenyl-3-(trifluoromethyl)-1H-pyrazol-5-ol
M583.neg	1.30	0.26	1.17	Nodiff	TRICARBALLYLIC ACID
M584.pos	-0.17	0.50	0.64	Nodiff	Triphenyl(propyl)phosphonium cation
M585.pos	0.95	0.11	1.47	Nodiff	Pro-Glu
M586.pos	0.14	0.56	0.59	Nodiff	Methanone, [1-(5-fluoropentyl)-1H-indazol-3-yl]-1-naphthalenyl-
M587.pos	-0.17	0.46	0.70	Nodiff	Glabrone
M588.pos	1.26	0.72	0.44	Nodiff	4-[5-(Benzylsulfanyl)-4-methyl-4H-1,2,4-triazol-3-yl]aniline
M589.pos	0.56	0.39	0.86	Nodiff	3-Cyano-7-hydroxycoumarin
M59.neg	0.43	0.19	1.28	Nodiff	Taurine
M590.pos	0.41	0.34	0.97	Nodiff	mollugin
M591.neg	-0.46	0.19	1.20	Nodiff	(2E,4E)-5-(3-(Hexopyranosyloxy)-8-hydroxy-1,5-dimethyl-6-oxabicyclo[3.2.1]octan-8-yl)-3-methylpenta-2,4-dienoic acid
M592.neg	-0.14	0.98	0.03	Nodiff	Schaftoside
M593.pos	-0.28	0.48	0.77	Nodiff	3,3-Dimethylpyrrolidin-2-one
M594.neg	-0.38	0.21	1.12	Nodiff	RHAMNOSE
M595.neg	0.39	0.40	0.85	Nodiff	Sedoheptulosan
M596.pos	0.49	0.38	0.86	Nodiff	Diisopropyl_sulfide
M597.neg	3.00	0.06	1.84	Nodiff	Gly-Met
M598.pos	0.45	0.24	1.14	Nodiff	2-Hydroxy-6-methylquinoline-3-carbaldehyde
M599.pos	-0.15	0.49	0.73	Nodiff	5-Amino-2-methoxyphenol
M6.neg	-0.34	0.40	0.81	Nodiff	7-Ketolithocholic acid
M60.neg	-0.41	0.06	1.65	Nodiff	Arabitol
M601.neg	0.02	0.78	0.25	Nodiff	5'-Fluoro-2'-hydroxy-4-methylchalcone
M602.neg	0.31	0.37	0.94	Nodiff	(R)-3-Amino-4-methylpentanoic acid
M603.neg	0.44	0.26	1.13	Nodiff	1-Isopropyl-1H-benzimidazole-5-carboxylic acid
M604.pos	0.29	0.41	0.81	Nodiff	1,5-Naphthalenediamine
M605.pos	-0.54	0.11	1.43	Nodiff	Licoagroside B
M606.neg	1.29	0.10	1.56	Nodiff	Ala-Phe
M607.pos	-0.59	0.56	0.60	Nodiff	Apigenin 7-glucuronide
M608.pos	-0.43	0.09	1.54	Nodiff	2-(2,6-Dimethylmorpholin-4-yl)ethanol
M609.neg	-0.49	0.28	1.11	Nodiff	Dihydrophaseic acid
M61.pos	0.70	0.35	0.91	Nodiff	Deoxyguanosine
M611.pos	-2.26	0.14	1.44	Nodiff	Dihydroxyacetone_Phosphate_Acyl_Ester
M613.neg	-0.84	0.03	1.80	Nodiff	4,5-Dimethoxy-2,3-dihydro-1H-isoindole-1,3-dione
M614.neg	-1.24	0.84	0.31	Nodiff	N-Acetyl-D-galactosamine 4-sulfate
M615.pos	0.14	0.48	0.76	Nodiff	Ile-Met
M616.neg	-0.30	0.21	1.14	Nodiff	2,3-Dihydroxy-3-methylbutyric acid
M617.pos	0.44	0.39	0.92	Nodiff	Bentazon
M618.pos	0.87	0.61	0.42	Nodiff	Dodemorph
M619.neg	-0.19	0.98	0.06	Nodiff	6-Thioinosine
M62.neg	0.87	0.56	0.74	Nodiff	Glycohyodeoxycholic acid
M620.neg	0.32	0.40	0.87	Nodiff	(10E,15Z)-9,12,13-Trihydroxyoctadeca-10,15-dienoic acid
M621.neg	1.38	0.04	1.71	Nodiff	1-Palmitoyl-2-hydroxy-sn-glycero-3-phospho-(1'-rac-glycerol)
M622.neg	-1.51	0.11	1.61	Nodiff	3,4-Dihydroxycinnamic acid (L-alanine methyl ester) amide
M623.neg	-0.24	0.53	0.75	Nodiff	DEOXYRIBOSE
M624.pos	-0.47	0.71	0.37	Nodiff	1-(1Z-Octadecenyl)-sn-glycero-3-phosphocholine
M625.pos	-0.53	0.56	0.59	Nodiff	4-Methylumbelliferyl .beta.-D-glucopyranoside
M626.neg	1.33	0.31	1.04	Nodiff	9-HPODE

M627.pos	-0.32	0.38	0.78	Nodiff	D-erythro-Sphingosine C-20
M628.pos	0.65	0.54	0.70	Nodiff	N-(3-Piperidinyl)propanamide
M629.neg	-1.40	0.03	1.74	Nodiff	4-Amino-2-(5-chloro-1,3-benzoxazol-2-yl)phenol
M63.neg	-1.13	0.05	1.67	Nodiff	2-Hydroxybutyric acid
M630.pos	0.00	1.00	0.02	Nodiff	Tripropylene glycol
M631.pos	1.28	0.18	1.20	Nodiff	1-[1,4-Dihydro-4-nonyl-5-(1-oxodecyl)-3-pyridinyl]-1-dodecanone
M632.pos	0.58	0.37	0.94	Nodiff	Oxopalmitoylcarnitine
M633.pos	-0.58	0.08	1.57	Nodiff	2-((4-Methylpiperazin-1-yl)methyl)-5,6,7,8-tetrahydrobenzo[4,5]thieno[2,3-d]pyrimidin-4-ol
M634.pos	1.14	0.26	1.11	Nodiff	Methanone, (3-methoxyphenyl)(1-pentyl-1H-indol-3-yl)-
M635.pos	0.80	0.01	1.95	Nodiff	Kukoamine B
M636.pos	1.39	0.28	1.07	Nodiff	Pro-Asp
M637.neg	0.51	0.38	0.92	Nodiff	4H-1,2,4-Triazole-3-thiol, 4-phenyl-5-(4-pyridyl)-
M638.neg	1.15	0.22	1.20	Nodiff	Val-Lys
M639.neg	0.84	0.38	0.88	Nodiff	4-Acetyl-3-ethyl-5-methyl-N-{2-methyl-5-[(methylamino)sulfonyl]phenyl}-1H-pyrrole-2-carboxamide
M64.neg	1.09	0.09	1.70	Nodiff	Methyl beta-D-Galactopyranoside
M640.neg	-0.19	0.98	0.01	Nodiff	(S)-5-Methylhydantoin
M641.pos	1.29	0.26	1.12	Nodiff	His-Thr
M642.neg	1.37	0.04	1.81	Nodiff	Leu-Glu
M643.pos	-0.46	0.17	1.35	Nodiff	4-Aminobutanamide
M644.pos	0.21	0.53	0.65	Nodiff	Pimonidazole
M645.neg	0.26	0.42	0.82	Nodiff	1-O-(3-Hydroxy-4,5-dimethoxybenzoyl)hexopyranose
M646.pos	-0.33	0.30	1.01	Nodiff	Betonicine
M647.neg	1.67	0.04	1.77	Nodiff	Thr-Ala
M648.pos	-0.50	0.36	0.80	Nodiff	N-Methylglycine N,N-dimethylamide
M649.pos	2.01	0.38	0.89	Nodiff	Val-Gly
M65.neg	-0.20	0.54	0.63	Nodiff	Kynurenic acid
M650.pos	-0.75	0.01	1.97	Nodiff	Dibenz(a,j)acridine
M651.pos	-0.47	0.53	0.66	Nodiff	1-(2-Methoxyethyl)piperazine
M652.pos	0.60	0.22	1.21	Nodiff	8-OH-dG
M653.pos	0.17	0.74	0.31	Nodiff	(R)-Aminocarnitine
M654.pos	-0.03	0.62	0.43	Nodiff	4-(Dimethylamino)phenylalanine
M655.neg	12.57	0.06	1.89	Nodiff	Val-Ser
M656.pos	0.45	0.34	0.95	Nodiff	N-Methyl-N-[2-oxo-2-(1-pyrrolidinyl)ethyl]amine
M657.pos	0.56	0.25	1.15	Nodiff	Leucylglycine
M658.pos	0.69	0.57	0.63	Nodiff	Piperazine-N,N'-bis(2-hydroxypropanesulfonic acid)
M659.pos	2.12	0.60	0.44	Nodiff	Pro-Met
M66.pos	0.52	0.55	0.52	Nodiff	Adenosine
M660.pos	0.42	0.45	0.77	Nodiff	1H-Indole-4-carboxaldehyde
M661.neg	-1.00	0.18	1.40	Nodiff	4,5-DIDEMETHYLSIMMONDSIN
M662.pos	-0.36	0.57	0.54	Nodiff	Cotinine
M663.pos	0.74	0.34	0.99	Nodiff	Thr-Met
M664.pos	-0.51	0.46	0.81	Nodiff	Trp-Thr
M665.neg	1.10	0.13	1.40	Nodiff	Tyr-Leu
M666.neg	-0.18	0.80	0.31	Nodiff	5-Methyl-1H-pyrazole-3-carbaldehyde
M667.neg	0.42	0.50	0.72	Nodiff	5-Methyl-2-oxo-1,3-oxazolidine-4-carboxylic acid
M668.neg	0.72	0.22	1.25	Nodiff	D-Saccharic acid 1,4-lactone
M669.pos	0.22	0.41	0.87	Nodiff	8-Methylcaffeine
M67.neg	0.17	0.46	0.72	Nodiff	Ursodeoxycholic acid
M671.pos	0.26	0.41	0.84	Nodiff	Met-Met
M672.pos	-0.83	0.65	0.41	Nodiff	1,3-Dimethyl-8-(1-piperidinyl)-3,7-dihydro-1H-purine-2,6-dione

M673.pos	0.04	0.43	0.77	Nodiff	Protoporphyrin IX
M674.pos	1.76	0.02	1.81	Nodiff	(2E,4E,10Z)-N-(2-Methylpropyl)hexadeca-2,4,10-trienamide
M675.neg	0.41	0.27	1.17	Nodiff	[(2-Aminoethyl)sulfanyl]sulfonic acid
M676.pos	0.22	0.23	1.21	Nodiff	Laccarin
M677.pos	-0.28	0.18	1.28	Nodiff	6-Amino-1,3-dimethyl-5-nitroso-2,4(1H,3H)-pyrimidinedione
M678.pos	-0.18	0.43	0.80	Nodiff	Neoandrographolide
M679.neg	-0.10	0.78	0.32	Nodiff	2-HYDROXY-4-(METHYLTHIO)BUTYRIC ACID
M68.pos	-0.26	0.75	0.31	Nodiff	N-Methylnicotinamide
M680.neg	-0.82	0.34	1.10	Nodiff	Zalcitabine
M681.pos	-0.56	0.08	1.70	Nodiff	Dulcin
M682.pos	1.13	0.02	1.80	Nodiff	5-(Ethoxymethyl)-2-methyl-4-pyrimidinamine
M683.neg	-0.10	0.48	0.71	Nodiff	Atropic acid
M684.neg	0.46	0.32	1.00	Nodiff	Probucol
M685.pos	1.14	0.19	1.22	Nodiff	3-(4-Hydroxyanilino)-5,5-dimethyl-2-cyclohexen-1-one
M686.pos	0.56	0.33	0.84	Nodiff	3-Hydroxy-b,e-caroten-3'-one
M687.neg	-0.37	0.40	0.81	Nodiff	2-[(2R,4aS,8S,8aS)-8-[2-[(4aS,7R,8aR)-7-(1-Carboxyethenyl)-1-hydroxy-4a-methyl-2-oxo-6,7,8,8a-tetrahydro-5H-naphthalen-1-yl]ethyl]-4a-methyl-7-oxo-1,2,3,4,8,8a-hexahydronaphthalen-2-yl]prop-2-enoic acid
M688.pos	2.18	0.20	1.24	Nodiff	Montecristin
M689.pos	0.40	0.68	0.46	Nodiff	LPI(18:0)
M69.neg	0.32	0.48	0.74	Nodiff	cis-9-Palmitoleic acid
M690.pos	1.68	0.22	1.22	Nodiff	LPC(16:1)
M691.neg	0.59	0.30	0.99	Nodiff	17-ODYA
M692.neg	0.17	0.37	1.01	Nodiff	D-Pinitol
M693.pos	-0.58	0.38	0.99	Nodiff	3-Hydroxyacetaminophen
M694.pos	-0.24	0.48	0.67	Nodiff	Asp-Arg
M695.pos	-0.47	0.25	1.20	Nodiff	Lys-Glu
M696.neg	0.21	0.42	0.81	Nodiff	Arg-Glu
M697.neg	-0.19	0.54	0.69	Nodiff	3'-Sialyllactose
M698.pos	1.45	0.04	1.77	Nodiff	Thr-Glu
M699.pos	0.13	0.51	0.63	Nodiff	Cyclocytidine
M7.neg	1.47	0.25	1.18	Nodiff	2'-O-Methyluridine
M70.neg	0.51	0.44	0.78	Nodiff	3-hydroxybenzaldehyde
M700.pos	0.34	0.25	1.13	Nodiff	2-Aminopyridine
M701.pos	0.34	0.25	1.21	Nodiff	N-Nitrosopyrrolidine
M702.neg	0.41	0.39	0.89	Nodiff	2-Keto-3-deoxyoctonic acid
M703.pos	-0.74	0.15	1.41	Nodiff	Piperazine-2-carboxylic acid
M704.pos	0.13	0.42	0.79	Nodiff	1,3-Dimethyl-6-(propylamino)-2,4(1H,3H)-pyrimidinedione
M705.neg	-0.14	0.85	0.10	Nodiff	2-Amino-5,5,5-trifluoropentanoic acid
M706.pos	0.30	0.50	0.74	Nodiff	Imazalil
M707.neg	-0.01	0.46	0.76	Nodiff	PERSEITOL
M708.pos	-0.67	0.58	0.55	Nodiff	2-Amino-6-(methylsulfanyl)-3,5-pyridinedicarbonitrile
M709.pos	-0.13	0.62	0.46	Nodiff	2-Methyl-4'-(methylthio)-2-morpholinopropiophenone
M71.neg	0.51	0.44	0.78	Nodiff	4-Hydroxybenzaldehyde
M710.pos	-0.33	0.16	1.31	Nodiff	N-Methylcalystegine_C1
M711.pos	1.35	0.18	1.31	Nodiff	[(7-Oxo-7H-benzo[de]anthracen-3-yl)sulfanyl]acetic acid
M712.pos	-0.20	0.33	1.00	Nodiff	8-Bromo-2,5-dihydro-3H-[1,2,4]triazino[5,6-b]indole-3-thione
M713.pos	0.84	0.33	0.95	Nodiff	Imazapyr
M714.pos	0.11	0.70	0.44	Nodiff	Piperidine
M715.neg	-0.42	0.15	1.29	Nodiff	METHYL GALACTOSIDE
M716.pos	-0.20	0.50	0.65	Nodiff	Dehydroabietic acid
M717.neg	0.34	0.40	0.94	Nodiff	N-Acetylgalactosamine_6-sulfate

M718.pos	0.50	0.21	1.11	Nodiff	2-(3,4,5-Trimethoxyphenyl)-1H-benzimidazole
M719.neg	-0.12	0.64	0.54	Nodiff	Cauloside C
M72.neg	0.17	0.46	0.72	Nodiff	Isodeoxycholic acid
M720.neg	-1.60	0.21	1.20	Nodiff	PyroGlu-Val
M721.pos	0.67	0.13	1.48	Nodiff	Tyr-Val
M722.pos	-2.29	0.45	0.73	Nodiff	2,3,4,5-Tetrahydro-6-(5-methyl-2-furanyl)pyridine
M723.pos	-0.87	0.61	0.57	Nodiff	2,2-Bis(hydroxymethyl)-3-quinuclidinone
M724.neg	-1.32	0.30	0.97	Nodiff	Ile-Glu
M725.neg	-1.69	0.34	1.05	Nodiff	6-Nitro-1,3-benzothiazol-2(3H)-one
M726.pos	-0.79	0.12	1.42	Nodiff	2-Cyclohexen-1-one, 4-[(1E)-3-(.beta.-D-glucopyranosyloxy)-1-buten-1-yl]-4-hydroxy-3,5,5-trimethyl-, (4S)-
M727.pos	-0.43	0.39	0.97	Nodiff	4-Amino-6-methoxy-3-quinolinecarboxylic acid
M73.pos	-0.26	0.75	0.31	Nodiff	6-Methylnicotinamide
M730.pos	-0.48	0.10	1.47	Nodiff	4-(N-Methylacetamido)benzoic acid
M731.pos	0.03	0.83	0.12	Nodiff	4-Oxo-1,4-dihydro-3-quinolinecarboxylic acid
M732.pos	2.80	0.11	1.53	Nodiff	Phe-Met
M733.neg	2.85	0.09	1.67	Nodiff	2-Hydroxy-1,3-dimethyl-9H-thioxanthen-9-one
M734.pos	-0.30	0.51	0.72	Nodiff	2-(4-Chlorophenyl)-4,5-dihydro-1H-imidazole
M735.neg	0.50	0.40	0.87	Nodiff	4-Imidazolidineheptanoic acid, 3-[(3R)-3-cyclohexyl-3-hydroxypropyl]-2,5-dioxo-, (4R)-rel-
M736.pos	-0.27	0.25	1.16	Nodiff	5-(4-Methyl-1-piperazinyl)-2-nitroaniline
M737.pos	2.34	0.15	1.42	Nodiff	Tetraethylammonium cation
M738.pos	0.48	0.37	0.88	Nodiff	3-Aminoquinoline
M739.pos	-0.05	0.57	0.54	Nodiff	Cirsimarin
M74.pos	-0.26	0.75	0.31	Nodiff	2-Methylnicotinamide
M740.pos	0.70	0.11	1.52	Nodiff	Daidzin
M741.neg	0.30	0.67	0.57	Nodiff	3-(2,5-Dioxo-1-pyrrolidinyl)benzoic acid
M742.pos	1.21	0.30	1.02	Nodiff	Octyl-methoxycinnamate
M743.pos	0.38	0.30	1.03	Nodiff	6-Dimethylaminopurine
M744.pos	0.70	0.17	1.36	Nodiff	4-(1-Pyrazolyl)benzaldehyde
M745.pos	-0.56	0.37	0.96	Nodiff	2,2-Diallylpiperazine
M746.pos	-0.67	0.37	0.80	Nodiff	5,6-Dihydroxy-7-methoxyflavone
M747.neg	-0.35	0.87	0.16	Nodiff	cis-4,10,13,16-Docosatetraenoic acid
M748.pos	0.28	0.50	0.63	Nodiff	3-(Benzylamino)cyclohex-2-en-1-one
M749.pos	-1.22	0.37	0.88	Nodiff	(+|-)-Currayangine
M75.pos	1.25	0.24	1.17	Nodiff	His-Gly
M750.pos	0.06	0.84	0.09	Nodiff	(E)-N-[2-(3-Indolyl)ethyl]-3-(4-hydroxy-3-methoxyphenyl)acrylamide
M751.neg	-0.15	0.67	0.46	Nodiff	Wogonin
M752.neg	2.18	0.20	1.31	Nodiff	Manninotriose
M753.pos	-0.19	0.47	0.83	Nodiff	Soyasaponin I
M754.pos	1.21	0.19	1.26	Nodiff	Palmitoleylcarnitine
M755.pos	0.68	0.17	1.37	Nodiff	SM(d34:1)
M756.pos	0.76	0.13	1.43	Nodiff	1,3,3-Trimethyl-2-[(E)-2-(2-methyl-1H-indol-3-yl)ethenyl]-3H-indolium cation
M757.neg	0.98	0.28	1.07	Nodiff	Asn-Lys
M758.pos	-0.26	0.68	0.41	Nodiff	Lys-Asn
M759.pos	0.04	0.55	0.58	Nodiff	Pro-Arg
M76.neg	-0.18	0.63	0.45	Nodiff	alpha-Ketoglutaric acid (alpha-KG)
M760.pos	-0.66	0.17	1.24	Nodiff	Lys-Asp
M761.pos	0.76	0.17	1.33	Nodiff	Methanone, (4-ethyl-1-naphthalenyl)(5-hydroxy-1-pentyl-1H-indol-3-yl)-
M762.pos	-2.29	0.86	0.22	Nodiff	Arg-Asn
M763.pos	-0.12	0.94	0.09	Nodiff	4-Hydroxy-N-desmethyltamoxifen
M764.pos	0.87	0.24	1.15	Nodiff	His-Glu

M765.pos	-0.07	0.81	0.25	Nodiff	Norflurazon
M766.pos	-0.25	0.30	1.10	Nodiff	Bortezomib__
M767.neg	0.56	0.24	1.22	Nodiff	Gln-Asp
M768.neg	1.44	0.05	1.80	Nodiff	Gln-Glu
M769.pos	-0.43	0.19	1.20	Nodiff	Malonyl-L-carnitine
M77.neg	0.03	0.71	0.44	Nodiff	Homoserine
M770.pos	0.53	0.42	0.77	Nodiff	Thr-Arg
M771.neg	-0.22	0.43	0.93	Nodiff	Bosutinib
M772.pos	-1.67	0.42	0.88	Nodiff	His-Asn
M773.pos	0.27	0.40	0.86	Nodiff	Val-Asp
M774.pos	1.25	0.06	1.69	Nodiff	Val-Glu
M775.pos	0.62	0.28	1.04	Nodiff	Pro-His
M776.pos	0.94	0.56	0.67	Nodiff	Gln-Gln
M777.pos	1.05	0.30	0.98	Nodiff	Pro-Gln
M778.pos	0.18	0.66	0.44	Nodiff	His-Ala
M779.pos	0.20	0.43	0.77	Nodiff	[(4,6-Dimethyl-2-pyrimidinyl)amino]acetic acid
M78.pos	0.07	0.88	0.21	Nodiff	Creatine
M780.pos	1.03	0.32	1.05	Nodiff	Pro-Ser
M781.pos	-0.46	0.20	1.29	Nodiff	2-Pyrrolidinone
M782.neg	1.55	0.78	0.40	Nodiff	Gln-Thr
M783.pos	-0.01	0.84	0.15	Nodiff	Methyl 4-(trifluoromethyl)benzoylacetate
M784.pos	-0.58	0.29	1.04	Nodiff	Carbidopa
M785.pos	0.27	0.41	0.80	Nodiff	1,2-Diaminocyclohexane
M786.neg	-0.54	0.31	0.95	Nodiff	(E)-3,10-Dihydroxy-4,9-dimethyldodec-6-enedioic acid
M787.pos	0.27	0.51	0.61	Nodiff	Val-Asn
M788.pos	0.95	0.06	1.68	Nodiff	Chromone
M789.pos	0.40	0.96	0.03	Nodiff	5-Nitro-2-(1-pyrrolidinyl)benzoic acid
M79.pos	-0.61	0.20	1.38	Nodiff	Ectoine
M790.pos	0.07	0.92	0.05	Nodiff	Pro-Tyr
M791.pos	-0.01	0.47	0.77	Nodiff	2-(1-Piperidyl)propan-2-ol
M792.pos	-0.33	0.37	0.94	Nodiff	(2R,3R)-5,7-dihydroxy-2-(3,4,5-trihydroxyphenyl)-3,4-dihydro-2H-chromen-3-yl 3,4,5-trihydroxybenzoate
M793.pos	0.34	0.47	0.67	Nodiff	4-Deoxypyridoxine
M794.pos	1.25	0.40	0.78	Nodiff	Rilmenidine
M795.neg	0.94	0.15	1.46	Nodiff	Leu-Gly-Leu
M796.neg	1.63	0.04	1.81	Nodiff	Thr-Val-Leu
M797.pos	-0.23	0.96	0.15	Nodiff	4-Heptenoic acid, 7-[(1R,2R,5S)-5-([1,1'-biphenyl]-4-ylmethoxy)-2-(4-morpholinyl)-3-oxocyclopentyl]-, (4Z)-rel-
M798.neg	-1.58	0.12	1.46	Nodiff	(24E)-12,15-Dihydroxy-3-(pentopyranosyloxy)-9,19-cyclolanost-24-en-26-oic acid
M799.pos	0.29	0.43	0.78	Nodiff	rac-trans-4-Cotininecarboxylic acid
M8.neg	-0.14	0.85	0.17	Nodiff	Thymine
M80.pos	0.40	0.56	0.54	Nodiff	3-Hydroxybutyrylcarnitine (Car(4:0-O))
M800.neg	-0.51	0.54	0.46	Nodiff	9-(2,3-Dihydroxypropoxy)-9-oxononanoic acid
M801.pos	-0.36	0.31	1.01	Nodiff	Methyldopa
M802.pos	-0.33	0.93	0.01	Nodiff	N-Methylanabasine
M803.neg	-0.46	0.50	0.78	Nodiff	Dorzolamide
M804.pos	-0.91	0.05	1.61	Nodiff	1,1-Dimethyl-4-phenylpiperazin-1-ium cation
M805.pos	0.58	0.29	1.06	Nodiff	Dianthoside
M806.pos	0.44	0.96	0.13	Nodiff	3-Piperidone
M807.pos	3.16	0.11	1.51	Nodiff	9-(5-O-Methylpentofuranosyl)-1,9-dihydro-6H-purin-6-one
M808.neg	-0.66	0.32	1.02	Nodiff	Garcinone C
M809.pos	0.67	0.12	1.47	Nodiff	5,7-Dihydroxy-4-phenyl-2H-chromen-2-one
M81.neg	0.22	0.40	0.92	Nodiff	Proline
M810.pos	-0.57	0.36	0.84	Nodiff	Hexadecyltrimethylammonium cation

M811.pos	-0.64	0.56	0.61	Nodiff	1-Stearoyl-2-arachidonyl-sn-glycero-3-phosphocholine
M812.pos	-0.22	0.32	1.00	Nodiff	3,4,5-Trimethoxy-N-(3-pyridinyl)benzamide
M813.pos	0.23	0.59	0.62	Nodiff	2-[(1-Methyl-1H-pyrazolo[3,4-d]pyrimidin-4-yl)amino]ethanol
M814.pos	-2.14	0.21	1.25	Nodiff	4-(4-Methyl-1-piperazinyl)butanoic acid
M815.pos	-0.10	0.48	0.77	Nodiff	DL-Norvaline
M816.pos	1.25	0.70	0.40	Nodiff	Ile-Ala
M817.pos	-0.51	0.38	0.79	Nodiff	1-Tetrahydro-2H-pyran-4-yl-4-piperidinamine
M818.pos	0.22	0.38	0.92	Nodiff	Rhamnazin
M819.pos	0.25	0.51	0.61	Nodiff	(2E)-3-Phenyl-1-(piperidin-1-yl)prop-2-en-1-one
M82.neg	0.21	0.97	0.00	Nodiff	Glyceric acid
M820.pos	2.32	0.19	1.27	Nodiff	Cohibin_D
M821.pos	-0.13	0.49	0.80	Nodiff	5,7,2'-Trihydroxyflavone
M822.pos	-0.02	1.00	0.03	Nodiff	Lauric diethanolamide
M823.neg	0.11	0.65	0.54	Nodiff	(S)-3,4-Dihydroxybutyric acid (lithium hydrate)
M824.pos	0.95	0.33	0.95	Nodiff	LPS(18:0)
M825.pos	-0.03	0.46	0.79	Nodiff	Hexadecenedioylcarnitine
M826.pos	0.63	0.37	0.95	Nodiff	Oxohexadecadienoylcarnitine
M827.pos	-0.33	0.73	0.41	Nodiff	PC(36:3)
M828.pos	-0.08	1.00	0.04	Nodiff	2-Phenylpiperazine
M829.pos	0.11	0.59	0.60	Nodiff	.beta.-Homoproline
M83.pos	0.89	0.06	1.65	Nodiff	Tyrosine
M830.pos	-0.27	0.51	0.62	Nodiff	Asp-Lys
M831.pos	-0.18	0.96	0.10	Nodiff	Arg-Asp
M832.pos	-0.37	0.28	1.13	Nodiff	Gln-Arg
M833.pos	-1.04	0.01	1.99	Nodiff	Ser-Asp
M834.neg	0.24	0.43	0.81	Nodiff	Glu-Asp
M835.pos	-1.19	0.11	1.42	Nodiff	1-(4-Aminobutyl)urea
M836.pos	1.35	0.30	1.03	Nodiff	Lys-Ile
M837.neg	0.65	0.15	1.48	Nodiff	Gln-Asn
M838.pos	1.40	0.28	1.05	Nodiff	Pro-Asn
M839.pos	0.25	0.66	0.50	Nodiff	5,5-Dimethylimidazolidine-2,4-dione
M84.neg	-0.41	0.24	1.10	Nodiff	Galactitol
M840.pos	0.62	0.33	1.04	Nodiff	Thr-Gly
M841.pos	1.69	0.04	1.80	Nodiff	Ala-Thr
M842.pos	-0.13	0.67	0.48	Nodiff	Statine
M843.pos	0.21	0.51	0.72	Nodiff	Leu-His
M844.pos	0.14	0.45	0.81	Nodiff	N-(4-Methyl-1,3-thiazol-2-yl)-N-(1-naphthyl)amine
M845.neg	-0.44	0.40	0.91	Nodiff	Tenofovir
M846.pos	0.52	0.28	1.06	Nodiff	Phe-Asn
M847.pos	-0.91	0.08	1.54	Nodiff	Phenacetin
M848.pos	-0.58	0.01	2.09	Nodiff	Cotinine_N-oxide
M849.pos	-0.59	0.45	0.75	Nodiff	.beta.-D-Glucopyranosiduronic acid, 4-(7-hydroxy-4-oxo-4H-1-benzopyran-3-yl)phenyl
M85.neg	-0.29	0.43	0.82	Nodiff	Acetylglycine
M850.neg	1.58	0.08	1.72	Nodiff	Diprotin B
M851.pos	1.48	0.07	1.65	Nodiff	Ile-Thr
M852.pos	-0.12	0.85	0.25	Nodiff	5-(2-Hydroxyethyl)-6-methyl-2-phenyl-4(3H)-pyrimidinone
M853.pos	1.21	0.76	0.22	Nodiff	Val-Gly-Val
M854.neg	-0.09	0.89	0.13	Nodiff	Citrinin
M855.pos	0.00	0.94	0.09	Nodiff	2-Methylbutylamine
M856.pos	1.66	0.07	1.65	Nodiff	Trp-Ala
M857.pos	-1.44	0.86	0.12	Nodiff	Acetophenone
M858.neg	0.39	0.40	0.85	Nodiff	1-(Propan-2-yl)-1H-benzimidazole-2-sulfonic acid
M859.pos	-0.57	0.92	0.09	Nodiff	2-Amino-4-methyl-6-(methylsulfanyl)-3,5-p

					yridinedicarbonitrile
M86.pos	0.25	0.42	0.83	Nodiff	Phenylalanine
M860.pos	1.23	0.13	1.42	Nodiff	Ile-Phe
M861.pos	1.67	0.03	1.92	Nodiff	2-Dimethylamino-6-hydroxypurine
M862.pos	0.85	0.26	1.13	Nodiff	3-Hydroxy-2-((9Z,12Z)-octadeca-9,12-dienoyloxy)propyl 2-(trimethylazaniumyl)ethyl phosphate
M863.pos	-3.33	0.87	0.08	Nodiff	N-Methyl-L-proline
M864.neg	-0.70	0.17	1.22	Nodiff	Phosphinodithioic acid, (4-methoxyphenyl)-4-morpholinyl-
M865.pos	0.27	0.40	0.85	Nodiff	Chrysogine
M866.pos	-0.54	0.12	1.46	Nodiff	1-Butyl-3-methyl-1H-imidazol-3-ium cation
M867.pos	-0.03	0.52	0.60	Nodiff	2-Methylbenzamide oxime
M868.pos	1.09	0.27	1.09	Nodiff	Panaquinquecol_1
M869.neg	-0.98	0.09	1.57	Nodiff	Isocalolongic acid
M87.pos	-1.07	0.30	0.96	Nodiff	LPS(18:1)
M870.neg	-0.25	0.56	0.58	Nodiff	1-(2-Chlorobenzyl)-5-oxo-3-pyrrolidinecarboxylic acid
M871.neg	0.28	0.47	0.83	Nodiff	Xylotetraose
M872.pos	0.34	0.21	1.29	Nodiff	LPC(19:0)
M873.pos	0.24	0.41	0.80	Nodiff	2-Heptylbenzothiazole
M874.pos	0.14	0.54	0.66	Nodiff	Mildronate
M875.neg	0.25	0.37	0.94	Nodiff	Carmoxirole
M876.pos	0.05	1.00	0.07	Nodiff	Pyridine-2,4-diamine
M877.pos	0.85	0.14	1.32	Nodiff	Gly-Arg
M878.neg	0.30	0.43	0.80	Nodiff	Lys-Thr
M879.pos	1.44	0.28	1.07	Nodiff	Lys-Ala
M88.pos	0.87	0.56	0.69	Nodiff	Glycoursodeoxycholic acid
M880.neg	-0.95	0.08	1.48	Nodiff	4-Benzyl-7-hydroxy-3-phenylcoumarin
M881.neg	1.44	0.04	1.83	Nodiff	Thr-Asp
M882.neg	0.08	0.66	0.53	Nodiff	8-Chloro-N-[3-(morpholin-4-yl)propyl]-5H-pyrimido[5,4-b]indol-4-amine
M883.pos	-0.26	0.12	1.53	Nodiff	Resorcinolnaphthalein
M884.pos	-0.37	0.22	1.21	Nodiff	4-Methyl-1H-pyrazole
M885.pos	0.09	0.57	0.62	Nodiff	His-Gln
M886.pos	-0.81	0.32	1.04	Nodiff	His-Ser
M887.neg	-0.46	0.27	0.99	Nodiff	DL-Isocitric acid lactone
M888.neg	-0.49	0.28	0.97	Nodiff	N-(tert-Butyl)-4-fluorobenzenesulfonamide
M889.pos	0.15	0.42	0.78	Nodiff	1,3,4,6,7,8-Hexahydro-1-methyl-2H-pyrimido[1,2-a]pyrimidine
M89.neg	1.06	0.11	1.56	Nodiff	Val-Ile
M890.pos	-0.69	0.28	1.13	Nodiff	N-Acetylvanilalanine
M891.pos	-0.48	0.10	1.48	Nodiff	Pergolide
M892.neg	0.23	0.43	0.88	Nodiff	Aurantiamine
M893.pos	-0.52	0.09	1.58	Nodiff	2-(4-Nitrophenoxy)ethanol
M894.pos	-0.43	0.24	1.27	Nodiff	N5-(1-Iminoethyl)-L-ornithine
M895.pos	0.31	0.41	0.79	Nodiff	Glutamylalanine
M896.pos	-0.87	0.05	1.76	Nodiff	Octopamine, N-feruloyl-
M897.pos	-0.20	0.22	1.25	Nodiff	Sulfamerazine
M898.neg	-0.34	0.42	0.78	Nodiff	4-Chloro-N-ethylbenzenesulfonamide
M899.neg	-0.45	0.38	0.84	Nodiff	Hemiphloin
M9.neg	1.09	0.18	1.34	Nodiff	Menadione bisulfite (sodium)
M90.neg	3.28	0.11	1.51	Nodiff	3'-O-Methylguanosine
M900.pos	-0.05	0.75	0.29	Nodiff	Phe-Gln
M901.neg	-0.19	0.98	0.00	Nodiff	Rabelomycin
M902.pos	-0.13	0.45	0.86	Nodiff	Iromycin A
M903.pos	0.21	0.37	0.92	Nodiff	Ile-Pro
M904.pos	-0.27	0.48	0.71	Nodiff	Alitame
M905.pos	0.42	0.39	0.92	Nodiff	2,4-Diamino-6-nitrotoluene
M906.neg	0.09	0.96	0.06	Nodiff	(2S)-2-[(2R,3S,7R,8R,8aS)-2,3,4'-Trihydroxy-4,4,7,8a-tetramethyl-6'-oxospiro[2,3,4a,5,6,7-hexahydro-1H-naphthalene-8,2'-3,8-dihydrofuro[2,3-e]isoindole]-7'-yl]-3-methylbuta

					noic acid
M907.pos	0.13	0.58	0.45	Nodiff	Glycerophospho-N-oleoylethanolamine
M908.pos	-0.36	0.18	1.27	Nodiff	(+)-Muscarine cation
M909.pos	0.79	0.48	0.68	Nodiff	Linoleoylcarnitine
M91.neg	0.27	0.41	0.85	Nodiff	Hypoxanthine
M910.pos	-0.57	0.63	0.45	Nodiff	Ethyl 4,5,6,7-tetrahydro-1H-indazole-5-carboxylate
M911.pos	0.78	0.28	1.07	Nodiff	Nicotinamide N-oxide
M912.pos	0.39	0.35	0.91	Nodiff	D8'-Merulinic_acid_C
M913.pos	-0.26	0.91	0.16	Nodiff	Methyl morpholine-3-carboxylate
M914.neg	0.54	0.54	0.55	Nodiff	2-(Hexopyranosyloxy)-3-hydroxypropyl (7Z,10Z,13Z)-hexadeca-7,10,13-trienoate
M915.pos	-0.11	0.44	0.88	Nodiff	5-Methylisoxazol-3-amine
M916.neg	-1.30	0.12	1.45	Nodiff	2-(4-Hydroxybenzylidene)-1H-indene-1,3(2H)-dione
M917.pos	0.83	0.30	1.02	Nodiff	Benzidine
M918.pos	0.32	0.50	0.60	Nodiff	AVOCADYNE
M919.pos	-0.35	0.40	0.86	Nodiff	Luteolin_4'-sulfate
M92.neg	-1.37	0.24	1.14	Nodiff	Hydroxyisocaproic acid
M920.pos	-0.34	0.52	0.69	Nodiff	Hexadecadienedioylcarnitine
M921.pos	-0.71	0.38	0.85	Nodiff	Oxododecanoylcarnitine
M922.pos	0.72	0.19	1.23	Nodiff	Sph(d18:2)
M923.neg	0.28	0.43	0.79	Nodiff	6-(4-Methylpiperazin-1-yl)-9H-purin-2-amine
M924.neg	-0.42	0.65	0.38	Nodiff	Asn-Asp
M925.pos	-0.20	0.39	0.88	Nodiff	Iloperidone
M926.pos	0.27	0.38	0.93	Nodiff	4-(3,4-Difluorophenyl)-6,7-dimethyl-3,4-dihydro-2(1H)-quinolinone
M927.pos	1.38	0.12	1.56	Nodiff	Desmethylolanzapine
M928.pos	0.67	0.22	1.18	Nodiff	Pro-Pro
M929.pos	-0.03	0.96	0.11	Nodiff	N,N-Dimethyl-L-valine
M93.neg	-1.37	0.24	1.14	Nodiff	2-Ethyl-2-hydroxybutyric acid
M930.pos	-0.32	0.24	1.19	Nodiff	3',4'-Dihydroxy-.beta.-naphthoflavone
M931.pos	0.02	0.47	0.76	Nodiff	Val-Arg
M932.pos	-0.27	0.51	0.63	Nodiff	Lys-Phe
M933.neg	-0.08	0.77	0.31	Nodiff	Tolfenamic acid
M934.neg	1.53	0.03	1.87	Nodiff	Thr-Ser
M935.pos	-0.71	0.02	1.88	Nodiff	2-(4-Amino-2-nitroanilino)ethanol
M936.pos	0.18	0.49	0.76	Nodiff	2-(1H-Indol-3-ylmethylene)malononitrile
M937.pos	-0.22	0.49	0.59	Nodiff	Pro-Val
M938.pos	0.51	0.28	1.06	Nodiff	Mardepodect
M939.pos	1.12	0.20	1.15	Nodiff	Pro-Ile
M94.neg	0.20	0.49	0.71	Nodiff	Benzoic acid
M940.pos	0.47	0.33	0.89	Nodiff	Tsugaric acid A
M941.pos	0.91	0.15	1.39	Nodiff	Buprenorphine
M942.pos	-0.10	0.45	0.78	Nodiff	2-Ethylbutan-1-amine
M943.pos	-0.02	0.99	0.06	Nodiff	Pilocarpine
M944.neg	-0.30	0.99	0.00	Nodiff	Thiolutin
M945.neg	-0.54	0.03	1.74	Nodiff	8-Prenylluteone
M946.pos	0.58	0.38	0.93	Nodiff	L-Serine methyl ester
M947.neg	0.75	0.14	1.38	Nodiff	2-[(2R,3S,7R,8R,8aS)-2,3,4'-Trihydroxy-4,4,7,8a-tetramethyl-6'-oxospiro[2,3,4a,5,6,7-hexahydro-1H-naphthalene-8,2'-3,8-dihydrofuro[2,3-e]isoindole]-7'-yl]-3-methylpentanoic acid
M948.neg	0.20	0.40	0.84	Nodiff	Methyl 3-(2,4-dichlorophenyl)-1H-pyrazole-5-carboxylate
M949.pos	-0.04	0.98	0.09	Nodiff	2-Imino-4-methylpiperidine
M95.neg	1.06	0.29	1.09	Nodiff	Valeric acid
M950.neg	-0.12	0.79	0.17	Nodiff	N-(1H-Benzimidazol-2-yl)-4-fluorobenzamide
M951.pos	-0.21	0.89	0.04	Nodiff	Soyacerebroside II

M952.pos	-0.04	0.90	0.05	Nodiff	Norharmane
M953.neg	0.30	0.72	0.28	Nodiff	Sulfoacetic acid
M954.pos	0.51	0.20	1.14	Nodiff	2-Methylpropanamine
M955.pos	1.81	0.44	0.82	Nodiff	Cadaverine
M956.pos	-0.42	0.40	0.85	Nodiff	Oxopalmitoleylcarnitine
M957.pos	1.39	0.06	1.67	Nodiff	Marimastat
M958.pos	0.67	0.34	0.88	Nodiff	LPE(P-16:0)
M959.pos	1.11	0.28	1.13	Nodiff	Oxotetradecadienoylcarnitine
M96.neg	0.13	0.69	0.40	Nodiff	12-Methyltridecanoic acid
M960.pos	-1.05	0.23	1.21	Nodiff	Dodecenoylcarnitine
M961.pos	-0.27	0.41	0.92	Nodiff	Benzene, 1-fluoro-2-[(1E)-2-(4-methoxyphenyl)ethenyl]-
M962.pos	0.44	0.06	1.62	Nodiff	Occidentoside
M963.pos	0.53	0.16	1.35	Nodiff	Yangonin
M964.neg	0.20	0.46	0.83	Nodiff	Methanedisulfonic acid
M965.pos	-0.12	0.51	0.73	Nodiff	1',3',3'-Trimethyl-6,8-dinitrospiro[chromene-2,2'-indoline]
M966.neg	0.03	0.94	0.18	Nodiff	Methyl 4-hydroxy-3,5-dinitrobenzoate
M967.neg	1.00	0.70	0.52	Nodiff	Isoquinoline, 5-[[(2S)-hexahydro-2-methyl-1H-1,4-diazepin-1-yl]sulfonyl]-4-methyl-
M968.neg	-0.76	0.97	0.07	Nodiff	Mevalonic acid 5-pyrophosphate
M969.pos	0.13	0.83	0.18	Nodiff	Argininic_acid
M97.neg	0.05	0.98	0.01	Nodiff	7,8-Dihydroxyflavone
M970.neg	-0.73	0.09	1.51	Nodiff	5-(4-Aminophenyl)-4-phenyl-2,4-dihydro-3H-1,2,4-triazole-3-thione
M971.neg	-0.80	0.23	1.12	Nodiff	N-Acryloyl-DL-aspartic acid
M972.pos	0.34	0.40	0.82	Nodiff	1-Chloro-3-(triphenylphosphoranylidene)acetone
M973.pos	0.71	0.95	0.11	Nodiff	1,2-Diethylhydrazine
M974.neg	0.20	0.41	0.85	Nodiff	3-[(Ethylanilino)methyl]benzenesulfonic acid
M975.pos	0.71	0.58	0.63	Nodiff	Dihydrosanguinarine
M976.neg	-0.31	0.81	0.19	Nodiff	Phenol, 2,6-difluoro-4-[4-[4-(4-methyl-1-piperazinyl)phenyl]-3-pyridinyl]-
M977.pos	1.12	0.07	1.69	Nodiff	Threoninyl-Serine
M978.pos	-0.44	0.25	1.10	Nodiff	Creatine, ethyl ester
M979.pos	-0.10	0.78	0.36	Nodiff	4H-1-Benzopyran-4-one, 6-arabinopyranosyl-8-.beta.-D-glucopyranosyl-5,7-dihydroxy-2-(4-hydroxyphenyl)-
M98.neg	0.51	0.09	1.59	Nodiff	Linoleic acid
M980.pos	0.71	0.61	0.61	Nodiff	N-Pentyl-5,6,7,8-tetrahydro[1]benzothieno[2,3-d]pyrimidin-4-amine
M981.pos	0.90	0.57	0.66	Nodiff	Gln-Leu
M982.neg	-0.32	0.40	0.86	Nodiff	risedronate
M983.pos	-0.76	0.35	0.96	Nodiff	L-Arginine, methyl ester
M984.neg	2.02	0.58	0.47	Nodiff	5,7-Dichloro-6-methyl-1,3-benzoxazole-2-thiol
M985.pos	0.16	0.58	0.52	Nodiff	5-Amino-1-pentanol
M986.pos	0.37	0.33	0.98	Nodiff	Val-Pro
M987.pos	0.11	0.43	0.84	Nodiff	Alanyl-Proline
M988.pos	0.35	0.87	0.19	Nodiff	Ser-Phe
M989.neg	-1.17	0.74	0.37	Nodiff	1-Benzyl-3-(1,3-thiazol-2-yl)thiourea
M99.neg	0.00	0.80	0.29	Nodiff	Oleic acid
M990.pos	-0.55	0.49	0.74	Nodiff	N,N-Dimethyl-4-(6-methylbenzo[d]thiazol-2-yl)aniline
M991.neg	0.27	0.48	0.82	Nodiff	Benzenesulfonic acid
M992.neg	-1.00	0.36	0.91	Nodiff	Tyr-Gly-Tyr
M993.pos	0.42	0.42	0.81	Nodiff	Diferuloylputrescine
M994.pos	1.18	0.32	0.96	Nodiff	cis-Parinaric acid methyl ester
M995.neg	-0.13	0.53	0.69	Nodiff	Daidzein 4'-sulfate
M996.neg	-0.63	0.36	0.89	Nodiff	5-Fluoro-1,3-dihydro-2H-benzimidazole-2-t

					hione
M997.pos	0.50	0.26	1.08	Nodiff	1,2,3,6-Tetrahydropyridine-4-carboxylic acid
M998.pos	0.71	0.33	0.97	Nodiff	3-Piperidinecarboxamide
M999.pos	0.77	0.61	0.57	Nodiff	Arg-Pro-Pro
